# An Injectable Meta‐Biomaterial: From Design and Simulation to In Vivo Shaping and Tissue Induction

**DOI:** 10.1002/adma.202102350

**Published:** 2021-08-27

**Authors:** Amélie Béduer, Fabien Bonini, Connor A. Verheyen, Martina Genta, Mariana Martins, Joé Brefie‐Guth, Josefine Tratwal, Aleksandra Filippova, Patrick Burch, Olaia Naveiras, Thomas Braschler

**Affiliations:** ^1^ Department of Pathology and Immunology Faculty of Medicine University of Geneva Rue Michel‐Servet 1 Geneva CH‐1211 Switzerland; ^2^ School of Engineering École Polytechnique Fédérale de Lausanne (EPFL) LMIS4. BM, Station 17 Lausanne CH‐1015 Switzerland; ^3^ Volumina‐Medical SA Route de la Corniche 5 Epalinges CH‐1066 Switzerland; ^4^ Department of Biomedical Sciences Laboratory of Regenerative Hematopoiesis University of Lausanne Rue du Bugnon 27 Lausanne CH‐1011 Switzerland; ^5^ CHUV Hematology Service Department of Oncology Rue du Bugnon 46 Lausanne CH‐1011 Switzerland

**Keywords:** elastic softening, injectable metamaterials, shaping, tissue reconstruction, vascularization

## Abstract

A novel type of injectable biomaterial with an elastic softening transition is described. The material enables in vivo shaping, followed by induction of 3D stable vascularized tissue. The synthesis of the injectable meta‐biomaterial is instructed by extensive numerical simulation as a suspension of irregularly fragmented, highly porous sponge‐like microgels. The irregular particle shape dramatically enhances yield strain for in vivo stability against deformation. Porosity of the particles, along with friction between internal surfaces, provides the elastic softening transition. This emergent metamaterial property enables the material to reversibly change stiffness during deformation, allowing native tissue properties to be matched over a wide range of deformation amplitudes. After subcutaneous injection in mice, predetermined shapes can be sculpted manually. The 3D shape is maintained during excellent host tissue integration, with induction of vascular connective tissue that persists to the end of one‐year follow‐up. The geometrical design is compatible with many hydrogel materials, including cell‐adhesion motives for cell transplantation. The injectable meta‐biomaterial therefore provides new perspectives in soft tissue engineering and regenerative medicine.

## Graphical abstract

Metamaterials gain unusual properties through their microstructure rather than specific chemistry. An elastic, porous, and injectable (EPI) microscaffold metamaterial is simulated and designed. The EPI provides experimental proof to the conjecture of elastic strain softening in microgel suspensions. Initially shapeable, it dynamically matches the local tissue mechanics, and templates long‐term soft tissue colonization and vascularization.

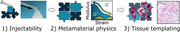

## 1 Introduction

Metamaterials display unusual physical properties rooted in their microstructure.^[^ ^1^, ^2^ ^]^ Some well‐known examples include materials with negative optical^[^ ^3^ ^]^ or acoustical index of refraction,^[^ ^4^ ^]^ negative Poisson‐coefficient materials^[^ ^5^ ^]^ as well as nearly arbitrarily shear‐deformable penta‐mode^[^ ^6^ ^]^ mechanical metamaterials. Although typical metamaterials are produced in crystal‐like repetitive structures,^[^ ^3^, ^4^, ^5^, ^6^ ^]^ this is not an absolute requirement, as for instance acoustical negative index materials have been realized as soft metafluids.^[^ ^7^ ^]^

Here, we develop an injectable metamaterial specifically designed to provide dynamic tissue mechanical matching. Indeed, tissues have strongly non‐linear elastic responses,^[^ ^8^ ^]^ yet it remains a challenge to match more than a given single mechanical parameter.^[^ ^9^ ^]^ We use here metamaterial design to provide, for the first time, dynamic matching of effective shear modulus over a wide range of deformation amplitudes in a biocompatible injectable. Our aim here is to apply the metamaterial development to soft tissue reconstruction in nearly arbitrary shapes, yet naturally following local tissue movement and mechanics.

Disease, trauma, surgery, and aging can indeed result in loss of soft tissue, producing major medical demand for tissue reconstruction.^[^ ^10^, ^11^ ^]^ Reconstructive procedures should be minimally invasive to decrease patient burden and surgical complications.^[^ ^12^ ^]^ Ideally, this is addressed by injectability through thin needles.^[^ ^10^ ^]^ To match patient‐specific defect geometries, surgeons desire in situ shapeability,^[^ ^13^ ^]^ and most importantly, shape‐ and volume‐stability following the procedure. Therefore, an ideal material‐based therapy would be 1) injectable, 2) shapeable, but then 3) volume‐ and shape‐stable under physiological conditions. Tissue‐matching mechanical properties,^[^ ^14^ ^]^ as well as high biocompatibility should allow it to ultimately give rise to native soft tissue.^[^ ^15^ ^]^

Mechanical tissue matching and injectability are inherently difficult to combine. The fluid‐like behavior required for injection limits stiffness; so many injectable agents are too soft^[^ ^16^ ^]^ to fully match local tissue.^[^ ^17^, ^18^ ^]^ Conversely, the solid‐like behavior required for volume‐stability or shape‐stability makes it difficult to deliver and shape large preformed scaffolds. This unmet need has spurred efforts to engineer injectable, shape‐fixable materials via in situ cross‐linking. Cross‐linking of both molecular hydrogel precursor solutions^[^ ^19^, ^20^ ^]^ and preformed microgel suspensions^[^ ^21^, ^22^ ^]^ has been proposed. A variety of cross‐linkers, but also peptide, DNA, polyelectrolyte complexes, or even cells impart self‐healing ability.^[^ ^21^, ^23^, ^24^ ^]^ Though such approaches improve mechanical properties, they also impose chemical constraints and raise concerns regarding biocompatibility and tissue integration.^[^ ^19^, ^25^ ^]^ To alleviate these constraints, chemically inert microgel suspensions can be injected instead; the challenge then becomes to achieve sufficient mechanical properties without excessive injection forces.^[^ ^26^ ^]^

Here, we reconcile minimally invasive delivery, shapeability, and long‐term stability through our novel paradigm: the injectable bio‐metamaterial.^[^ ^2^ ^]^ We show that physical interlocking of geometrically designed particles restores desirable implant mechanics even after liquefaction for injection. In particular, we engineer a novel reversible elastic softening transition^[^ ^27^ ^]^ into the injectable biomaterial. The phenomenon of elastic softening with increasing deformation, yet without the large‐scale plastic deformation characteristic for strain‐softening in alloys and plastics,^[^ ^28^ ^]^ has so far been described in selected actin and cellulose hydrogels.^[^ ^27^ ^]^ It has also been conjectured on theoretical grounds in microgel suspensions.^[^ ^29^, ^30^ ^]^ Here, we provide robust engineering principles, and use elastic softening to match endogenous rheological strain softening, found among others in adipose tissue.^[^ ^17^, ^18^ ^]^ This provides the desired dynamic mechanical matching. It also removes the size and shape constraints encountered with preshaped injectable scaffolds,^[^ ^31^, ^32^ ^]^ as the material offers a time‐window for shaping and sculpting after facile injection. The metamaterial exhibits excellent biocompatibility, gets colonized by vascularized host tissue in the desired shape, and can easily be adapted to extended applications such as cell delivery without losing its favorable properties.

Taken together, we successfully integrate the design criteria informed by clinical needs to engineer a tri‐state meta‐biomaterial that combines injectability, solid‐state softening/healing, and tissue‐mimicking volume‐stability.^[^ ^17^, ^18^ ^]^ With a design driven by in silico metamaterial simulation, this unique combination enables a minimally invasive, personalized treatment in soft tissue engineering and beyond.

## 2 Results and Discussion

### 2.1 Elastic Porous Injectable Meta‐Biomaterial

**Figure 1:**
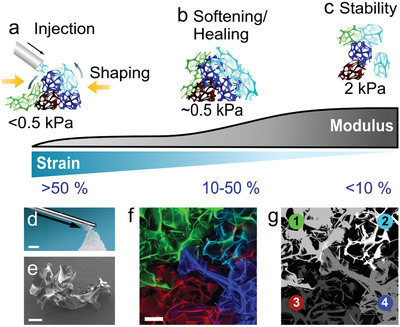
Elastic, porous, and injectable (EPI) meta‐biomaterial. a) Injectability: particle liquefaction and mobility under high strain enables minimally invasive delivery and respectively shapeability near the yield point. b) Reversible elastic softening: dynamic matching of local tissue mechanics: strain‐dependent stiffness at intermediate, physiological strains. c) Shape and volume stability: fully interlocked particle state enables long‐term 3D stability under low strains. d) Macroscopic demonstration of the material properties, depicting fluid‐like ejection of particles through a cannula and immediate solid‐like 3D shape stability. Scale bar: 4 mm e) Scanning electron microscopy picture of a single porous particle (brightness proportionally enhanced). f) Confocal image showing four interlocking porous particles (maximal intensity z projection). g) Particle identity in (f), determined by thresholding the individual color channels, and for the doubly labeled particle, colocalization of blue and green after correction for chromatic aberration. Scale bars: 200 µm for (e) and (f).

The concept of our elastic, porous, and injectable (EPI) meta‐biomaterial is illustrated in **Figure** **1**. Under high strain (imposed by injection), the material fluidizes and behaves as a liquid (Figure 1a). At the limit of yielding, smooth shaping at modest forces is possible. Under intermediate, physiological strains^[^ ^33^ ^]^ the EPI biomaterial is engineered to display an elastic softening transition, with dynamic metamaterial softening and self‐healing allowing matching of local tissue mechanics (Figure 1b). At rest, the self‐healing process is complete and the EPI biomaterial behaves as a soft solid with shear moduli matching the static native tissue properties (Figure 1c).^[^ ^17^, ^18^ ^]^

The EPI biomaterial consists of an interlocking suspension of highly irregular, sponge‐like microparticles. Its unique material design enables both fluidic injection through a cannula and shapeable 3D stability. A macroscopic demonstration of the behavior is provided in Figure 1d, while Figure 1e shows the irregular, porous particle morphology. Figure 1f,g demonstrates microscopic particle interlocking.

### 2.2 In Silico Design

To design the EPI biomaterial, we first performed a discrete element simulation which translated clinical requirements into design rules to guide our fabrication strategy. To reiterate, an ideal tissue‐filling biomaterial should display injectability, shapeability, volume‐stability, biocompatibility, and dynamic, strain‐dependent tissue‐matching mechanical properties.

**Figure 2:**
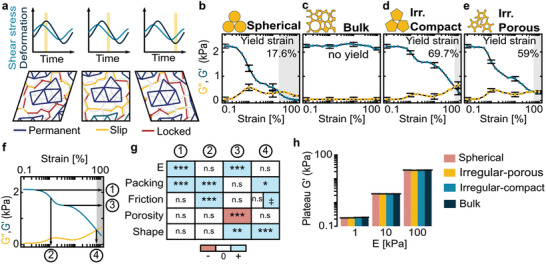
In silico simulation and design of the EPI meta‐biomaterial. a) Graphical overview of the simulation. b–e) Elastic storage and viscous loss modulus (*G*′ and *G*″) for: b) a dense suspension of frictional spheres, c) a bulk material formed by full cross‐linking of every neighboring sphere, d) a dense suspension of discrete, compact, irregular particles formed from neighboring spheres, and e) a dense suspension of discrete, irregular particles with a low cross‐link density and free contact interfaces. f) Characteristic rheological response with accompanying storage modulus and strain values. ① = low‐deformation limit *G*_0_′, ② = softening transition, ③ = soft plateau stress, ④ = yield strain. g) Overview of the influence of the model parameters on the characteristic values defined in (f). ‡ The friction coefficient has a magnitude‐dependent effect, see Figure S3, Supporting Information. h) Influence of the Young modulus of the constituent material on the low‐strain limit storage modulus for the different particle geometries. Error bars = one standard deviation. n.s. = not significant. Sample size and statistical testing information in Table S7, items 1–26, Supporting Information.

A full description of our simulation is provided at CodeOcean:^[^ ^34^ ^]^ ready‐to‐use source code, examples, installation guide, manual including developments over prior literature,^[^ ^29^, ^35^ ^]^ and API documentation. The simulation models the interaction between spherical particles by central and frictional forces,^[^ ^29^ ^]^ optionally with permanent cross‐links to constitute both irregular and porous microparticles (**Figure** **2**a). Analogous to the empirical characterization of soft tissue fillers,^[^ ^13^ ^]^ we performed in silico oscillatory shear rheometry by applying time‐varying strain (Figure 2a) and extrapolating the material's stiffness (elastic storage modulus, *G*′) and deformability (viscous loss modulus, *G*″).^[^ ^29^, ^36^ ^]^

We first simulated four prototypical scenarios: a simple suspension of frictional spherical microgels (Figure 2b and Video S1, Supporting Information), a fully cross‐linked bulk material (Figure 2c and Video S2, Supporting Information), densely cross‐linked irregular particles (Figure 2d and Video S3, Supporting Information) and loosely cross‐linked particles with decreased internal cross‐linking density (Figure 2e and Video S4, Supporting Information). An elastic softening transition was found for the frictional spherical microgels, as expected (Figure 2b, absent in non‐frictional control, Figure S1, Supporting Information).^[^ ^29^ ^]^ The yield strain was however substantially below the 50% required for a material^[^ ^13^ ^]^ to withstand physiological movement.^[^ ^33^ ^]^ Bulk cross‐linking (Figure 2c) abolished yielding altogether, thereby preventing scaffold injectability. Irregular microparticles (Figure 2d) finally exhibited yield strain well above 50%, successfully combining injectability and in vivo stability. This established our first design rule: the material should be a suspension of irregular rather than spherical particles.

Quantitatively, the elastic softening transition was better preserved in loosely (versus densely) cross‐linked particles (Figure 2e). Softening is critical for both in situ shapeability and mechanical matching of local adipose tissue (which also demonstrates strain softening).^[^ ^17^, ^18^ ^]^ We thus obtained our second design rule: the particles should have a low density of internal cross‐links with frictional intraparticle porosity.

With our first two design rules established, we generalized the desired rheological behavior (Figure 2f) and performed systematic analysis of the model's parameters (Figure 2g and Figure S2, Supporting Information). The analysis confirmed that geometric parameters (particle shape, porosity) dominated the high‐strain behaviors^[^ ^37^ ^]^ of yielding (④ in Figure 2f,g) and the soft plateau (③). The mechanical parameters (friction, packing, Young's modulus) instead dominated the low‐strain behaviors of softening (②) and low‐strain plateau shear modulus (①).

As a starting point for elastic softening at higher deformation, our material still needs to match the static elastic properties of native soft tissue at low deformation (Young's and shear moduli in lower kPa range).^[^ ^17^, ^18^ ^]^ Figure 2h indicates that the low‐strain storage modulus was proportional to the Young's modulus of the constituent material (linear regression, *P* = 8 × 10^−88^), but independent of particle geometry (*P* = 0.50) or cross‐linking density (*P* = 0.29) (Supporting Information, Table S7, item 26). Thus, we obtained our third design rule: the bulk precursor from which we derive our microparticles should have a storage modulus close to the low‐strain limit of adipose tissue.

The first three design criteria produce optimal rheology for injectability, shapeability, and tissue‐matching stability. Our fourth and final design rule stems from known pore size requirements to ensure vascularization and colonization: the microparticles should have a mean pore size of at least 50 μm.^[^ ^38^, ^39^ ^]^

### 2.3 Synthesis and Mechanical Characterization

Our in silico analysis revealed that an injectable, shapeable, and volume‐stable material with elastic strain softening could be achieved with a densely packed suspension of elastic, frictional microparticles with irregular, porous geometry (Figure 2). We base our synthesis on the cryogelation^[^ ^40^ ^]^ of carboxymethylcellulose (CMC).^[^ ^31^, ^38^ ^]^ This choice is motivated by biocompatibility,^[^ ^38^ ^]^ simplicity, and scalability. We prepared CMC reaction mixtures (cross‐linking by adipic acid dihydrazide^[^ ^41^ ^]^) and transferred them to −20 °C freezers to obtain bulk cryogels. Polymer content was adjusted to obtain porous scaffolds with storage moduli (*G*′) of 2.4 ± 0.9 kPa (Figure S4, Supporting Information), thereby satisfying design rule #3 (the bulk precursor *G*′ should match the low‐strain limit of adipose tissue).^[^ ^17^, ^18^ ^]^

**Figure 3:**
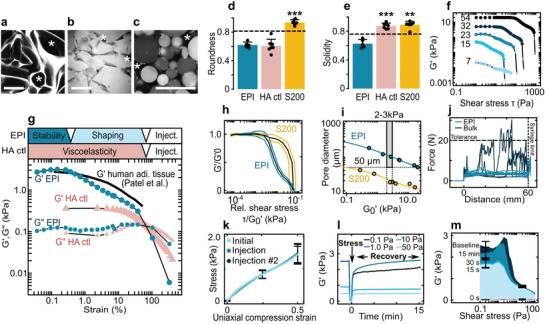
In vitro characterization of the EPI meta‐biomaterial. a–c) Confocal images of the EPI biomaterial (stained with rhodamine 6G) (a), HA ctl (hyaluronic acid control):commercial hyaluronic acid filler (stained with rhodamine 6G) (b), and Sephacryl S200 (autofluorescence) (c). The stars denote pore space. Scale bars = 200 µm. d) Roundness and e) solidity of EPI biomaterial, HA ctl, and Sephacryl S200. Dashed lines represent the threshold for which particles are: d) irregular (below) and spherical (above) and e) porous (below) and non‐porous above (thresholds from Figure S6, Supporting Information, in Supplementary 1). f) *G*′ of the EPI biomaterial as a function of applied oscillatory stress for various EPI biomaterial polymer concentrations (mg mL^−1^). Large symbols (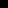): solid‐like behavior (*G*′ > *G*″); small symbols (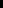): liquid‐like behavior (*G*″ < *G*′). Each curve represents a single measurement. g) Comparison of the EPI biomaterial (26 mg mL^−1^) to HA ctl (single samples) and replotted literature *G* data on human adipose tissue.^[^ ^17^ ^]^ h) Normalized master curves for EPI and Sephacryl 200, error lines ± one standard deviation. i) Pore size versus low‐stress limit *G'* *(G*_0_′)values. Gray box (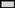): 50 µm minimal pore size and a *G*_0_′ value between 2 and 3 kPa required for matching adipose tissue.^[^ ^17^, ^18^, ^38^, ^39^ ^]^ j) Force required to eject bulk carboxymethylcellulose cryogel and EPI biomaterial through a 20‐gauge needle. k) Uniaxial compression analysis of a sample before passage through the cannula, after the first passage, and after two consecutive passages. l) Recovery of the *G*′ values after a period of liquefying shear (“Stress”). m) Level of *G*′ recovered after different time‐points as a function of continuous oscillatory shear stress (same data set as for [l]). Error bars = one standard deviation. Sample sizes and statistical testing details in Table S7, items 28–32, 35–38, 74, 75, 92, and 93, Supporting Information.

Through forceful extrusion (10–15 bar, 2–3 mL s^−1^, 22‐gauge catheter) we fragmented the suspended porous precursor scaffold^[^ ^31^ ^]^ into irregular porous microparticles with a diameter of 805 ± 363 μm (**Figure** **3**a,d,e and Figure S5, Supporting Information). The resulting EPI biomaterial displayed an intricate and widely connected pore morphology (Figure 3a,e). Using distinctly fluorescently labeled particles, the intraparticle pore space accounts for about 40% of the porosity and the remainder arising between the tightly interlocking irregular particles (Figures S8 and S9, Supporting Information). This unique pore structure contrasted starkly with traditional microgel suspensions as found in commercial hyaluronic acid controls (HA ctl) or microbead suspension Sephacryl S200,^[^ ^42^ ^]^ where porosity is limited to the small interstices between particles (Figure 3b,c,e, more details given in Figure S6, Supporting Information). The abundance of large frictional pore spaces fulfilled design rules #2 and #4, while the exceptional particle irregularity fulfilled #1. Thus, we obtained a novel biomaterial that satisfied all of the simulation‐defined design criteria.

With simulation and fabrication complete, we then performed extensive physical characterization. First, we investigated the presence of an elastic softening transition with high yield strain, as predicted by our model (Figure 2). When subjecting the EPI biomaterial to increasing oscillatory shear (Figure 3f and Figure S11, Supporting Information) we indeed observed a stable elastic plateau, a unique softening transition, and yielding at high strain (62 ± 5%) over a range of polymer concentrations (Figure S12, Supporting Information). Normalization to the low‐strain plateau value (*G*_0_′)^[^ ^43^ ^]^ confirmed that these features were conserved across all tested EPI concentrations, but absent in the spherical control suspension (Figure 3h). Further, in agreement with our simulation, the yield strain was significantly lower for spherical particles than for irregular particles (EPI) (24 ± 6%, *P* = 1.4 × 10^−5^, Figure S12, and Table S7, item 27, Supporting Information). We therefore succeeded in engineering a novel meta‐biomaterial that displayed not only elastic behavior with high‐strain yielding, but also a new softening transition to enable both shaping and tissue matching.

Our next aim was to match static (low‐strain) tissue mechanical properties,^[^ ^17^, ^18^ ^]^ while conserving a pore size greater than 50 µm for vascularization.^[^ ^38^, ^39^ ^]^ Figure 3i demonstrates an inverse relation between material stiffness (*G*_0_′) and pore diameter. Fluid removal indeed increases stiffness (Figure S10, Supporting Information) at the expense of pore fraction and size (Figure S7, Supporting Information). The EPI biomaterial could match adipose tissue stiffness (*G*_0_′ = 2–3 kPa)^[^ ^17^, ^18^ ^]^ while still maintaining adequate pore size (100–120 μm, Figure 3i). In spherical Sephacryl S200, sufficient stiffness could only be achieved at insufficient pore size (and vice versa). The EPI design thus specifically enables the joint fulfillment of both mechanical and geometric requirements.

We assessed dynamic tissue‐matching by rheological comparison of the EPI biomaterial to published human adipose tissue behavior.^[^ ^17^ ^]^ We find that the EPI biomaterial mimicked the tissue response over a wide range of deformations as desired (Figure 3g; comparison to further literature data^[^ ^18^ ^]^ and measurements on mouse adipose tissue in Figure S17, Supporting Information). In addition, the EPI biomaterial showed yielding similar to a commercial hyaluronic acid filler control (Figure 3g, HA ctl), which consists of irregularly shaped microparticles (Figure 3b,d),^[^ ^44^ ^]^ confirming the predicted importance of this feature for high yield‐strain (57 ± 7% for HA ctl, *P* = 1.0 vs EPI, Figure S12 and Table S7, item 27, Supporting Information). Thus, the EPI biomaterial displays optimal yielding while also achieving unprecedented dynamic tissue‐matching through the engineered strain‐softening.

For minimally invasive delivery, the EPI biomaterial must be injectable. Figure 3j confirms smooth injection at forces well below the clinical upper tolerance of 20 N^[^ ^24^ ^]^ (Table S7, item 75, Supporting Information). Fragmentation and particle compressibility are both essential, as comparison to unfragmented bulk material (Figure 3j) and literature on stiffer (≈30 kPa) porous silk particles shows.^[^ ^26^ ^]^ The EPI biomaterial can be injected through conduits up to 22 gauge without change in particle size.^[^ ^45^ ^]^ At very low injection rates, a challenge can be the separation of pore fluid and solid particle content (Figure S14 and S15 Supporting Information), but particle size and pore fluid viscosity adaptation can further extend EPI delivery, including through smaller conduits (up to 27 gauge).^[^ ^45^ ^]^ The uniaxial compression response was identical pre‐ and post‐injection, confirming conservation of EPI material properties (Figure 3k). The time‐course of physical self‐healing finally is shown in Figure 3l,m. Low but stable *G*′ values are rapidly restored, while full recovery required more than 10 min under low shear (Figure S16, Supporting Information). Rapid minimal self‐healing provides initial implant stability and most likely prevents undesirable spreading after implant injection, while subsequent full recovery achieves matching of local tissue mechanics.

In summary, we have engineered an EPI metamaterial (Figure 1) according to the design specifications derived from clinical criteria and in silico simulation (Figure 2). Physical characterization demonstrates the material's unique tri‐phasic rheology enabling facile injection, progressive self‐healing, and exquisite mimicry of tissue mechanics through elastic strain softening while maintaining adequate porosity for tissue ingrowth (Figure 3).

### 2.4 In Vivo

With physical characterization complete, we next investigated the in vivo performance of the EPI meta‐biomaterial. Specifically, we examined minimally invasive delivery, in situ shapeability, long‐term maintenance, as well as biocompatibility and tissue integration.

**Figure 4:**
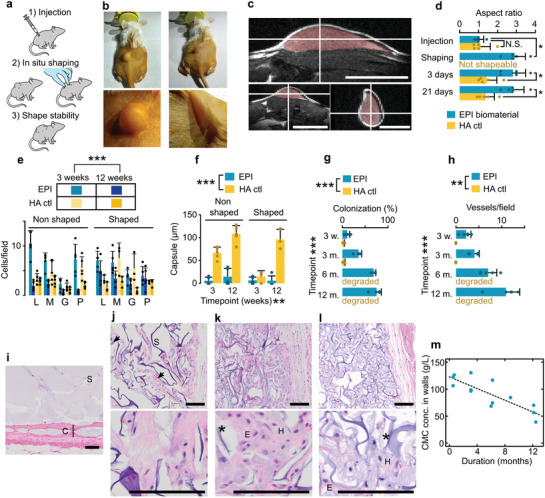
In vivo application, biocompatibility, and long‐term assessment of the EPI meta‐biomaterial. a) Experimental workflow. b) A 400 µL bolus of the EPI biomaterial after minimally invasive injection and 3D in situ shaping by application of moderate external forces. c) Magnetic resonance imaging of the EPI implantation and EPI labeled in false color (center of injected material at hairline crossing). Scale bars = 1 mm. d) Quantification of the maintenance of the shape by the aspect ratio of length along the injection direction to width of the implant for EPI and HA ctl. e) Quantification of inflammatory cells in the tissues surrounding EPI biomaterial and HA control material, per high‐power field of view (0.36 mm^2^). L = Lymphocytes, M = Macrophages, G = Giant foreign body cells, P = Polymorphonuclear cells. f) Quantification of capsule formation at 3 weeks and 3 months. g) Quantification of cellular colonization by area fraction occupied by cellular ingrowth. h) Vascularization by quantification of vascular ingrowth per field of view. i) Histology of HA control material at 3 months. Scale bar = 500 µm. Vasculature outlined by arrows, scaffold, in purple, by the letter “S,” and capsule by “C” with bar indicating width. j–l) Histology of the EPI biomaterial at 6 (j), 9 (k), and 12 (l) months, showing increasing scaffold degradation. Degrading scaffold denoted by stars, E = example of eosinophilic region and H = example of region stained by hematoxylin. Error bars = one standard deviation. Scale bars = 100 µm. m) Degradation kinetics assessed by the local carboxymethylcellulose (CMC) concentration in the remaining fragments. Statistical testing details and sample size in Table S7, items 43–54 and 63, Supporting Information.

The EPI biomaterial was manually injected into the subcutaneous space of CD1 mice and gently shaped by external force (**Figure** **4**a,b). Shear‐yielding enabled facile delivery through a 20‐gauge catheter, and magnetic resonance imaging (MRI) confirmed that the material behaved as a well‐defined, cohesive implant in the dermal space (Figure 4c). During a post‐injection shaping window (≈20 min), the material could be manipulated in situ to produce a new shape, which was retained spontaneously (Figure 4b–d, Video S5, Supporting Information). For comparison, we injected a bolus of a hyaluronic acid dermal filler (HA control) but could not permanently shape it. Instead, this material slid away from the applied force or spread into the surrounding tissues (at high force). After the shaping window, we assessed long‐term stability by gently attempting to reshape the existing volume, but found the shape remained remarkably stable over time despite repeated mechanical challenge (Figure 4d). Therefore, we successfully engineered a shapeable material with substantial lifting capacity for 3D tissue reconstruction.

Next, we quantitatively assessed the immune cell populations near the tissue interface of the implanted materials (Figure 4e). Summarizing, both materials exhibit similar low to moderate inflammatory response decreasing over time, for the HA control material in accordance with its known favorable clinical profile.^[^ ^46^ ^]^ Following an acute tissue response, inflammation indeed decreased significantly by 3 months for both materials (*t*‐test, *P* = 3 × 10^−8^, Table S7, item 49, Supporting Information), with similar overall levels of inflammation between the materials (*t*‐test, *P* = 0.33, Table S7, item 47). The HA control shows a somewhat stronger capsule formation (Figure 4i) than the EPI scaffold (Figure 4f,j,k,l; *P* = 9.6 × 10^−10^, Table S7, item 51), presumably in line with desired collagen deposition.^[^ ^46^, ^47^ ^]^ The shaping process itself had no significant effect on either inflammation (*P* = 0.74, Table S7, item 48) or encapsulation (*P* = 0.11, Table S7, item 51). This points toward at most minor effects of shaping on biocompatibility, and also excludes breach of sterility during the procedure. Regarding possible systemic effects, we routinely monitor survival and weight gain, and found no significant differences between the products, and also found no particularities on occasional liver histologies (data not shown).

Finally, we investigated the long‐term outcomes of EPI implantation. Colonization and vascularization increased over time (Figure 4g,h) and at 6 months (Figure 4j) the pore space was mostly colonized by fibrovascular tissue. Progressing to 9 months and 1 year (Figure 4k,l), scaffold degradation proceeds, with concomitant uptake of hematoxylin‐stained material by cells. Despite substantial biodegradation at 6–12 months (Figure 4m, *P* = 3.5 × 10^−4^ for linear regression, Table S7, item 63, about 50% degradation at 1 year as judged by the decrease in local CMC concentration), the fibrovascular meshwork persisted to the end of follow‐up. Injection of an EPI version with lower polymer content with more advanced biodegradation at 1 year suggested potential connective tissue recovery after scaffold degradation (Figure S19, Supporting Information). Taken together, our in vivo results confirmed that the unique rheological and morphological properties of the EPI biomaterial enabled the novel capacity to inject, shape, stabilize, and induce customized, vascularized 3D tissues.

### 2.5 Metamaterial Generalization

**Figure 5:**
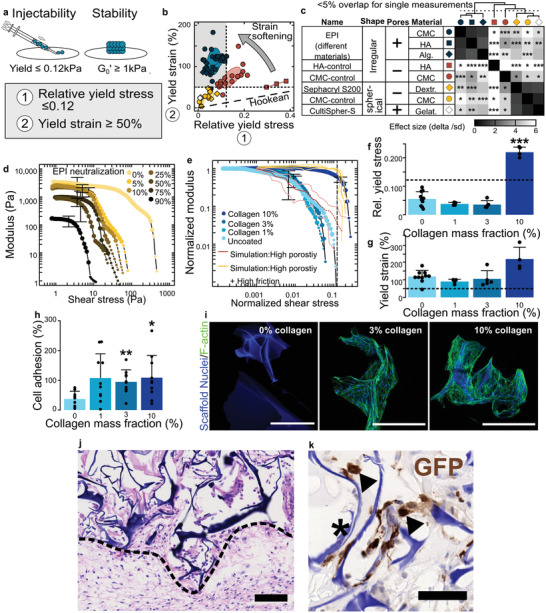
Generalization of the EPI metamaterial concept. a) Main parameters for high‐strength, high‐yield injectables: the relative yield stress describes the balance between injectability and stiffness; yield strain the resilience to physiological deformation. b) Classification of eight different particle suspension materials according to their relative yield stress and yield strain. Legend in common with (c). Gray target area (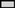): maximum relative yield stress 0.12, 50% yield strain or more for physiological deformation (c.f. [a]). Theory line: Hookean (linear) behavior with 100% yield strain equal to a relative yield stress of 1.0. 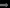 Departure above the Hookean line indicates the strength of elastic strain softening. c) Multivariate pairwise comparison and unsupervised clustering of the eight particle suspension materials. CMC = Carboxymethylcellulose; HA = Hyaluronic acid; Alg = Alginate; Dextr. = Dextrane; Gelat. = Gelatine. d) Influence of backbone charge on the rheological stress sweep, by acid–base titration. 0% neutralization designates the reference EPI material, 100% neutralization corresponds to full protonation of the carboxylate groups. e) Rheological properties (master curves) for collagen‐coated EPI material, with increasing mass fraction of collagen. Comparison with simulation with non‐linear compression law to quantitatively emulate porous structure, with low (*µ* = 0.01) and high (*µ* = 0.8) friction coefficient *µ*. f) Relative yield stress and g) yield strain of collagen‐coated EPI. h) Cell‐adhesion (OP‐9 cells) to uncoated and variously collagen‐coated EPI, at 1 h. i) Confocal image of collagen‐coated EPI with OP‐9 after 5 days in culture, stained for 4′,6‐diamidino‐2‐phenylindole (DAPI) (nuclei) and F‐actin (cell microfilament).    Scale bars = 500 µm. j) Hematoxylin and eosin (H&E) histology of GFP^+^‐OP‐9‐loaded EPI at 3 weeks. The dashed line outlines the boundary of the implant. Scale bar =1 00 µm. k) 3,3′‐Diaminobenzidine (DAB) (brown) staining showing the presence of GFP^+^‐OP‐9 (black arrows) in the EPI scaffold (star) at 3 weeks. Scale bar = 1000 µm. Statistical testing details and sample size inTable S7, items 59–62 and 64–66, Supporting Information.

Having established the material design rules and tissue induction capacity of our particular CMC‐based formulation, our next aim was to define the general scope of the EPI metamaterial concept. We capture the balance between injectability and in situ stiffness by the relative yield stress (**Figure** **5**a). This ratio should be lower than 0.12, enabling a maximum yield stress of 0.12 kPa as measured on HA control at a minimal stiffness *G*_0_′ of 1 kPa (separating the firmer subcutaneous adipose tissue from soft visceral fat or HA control, Figure S17, Supporting Information). As before, we also require a yield strain larger than 50% to withstand physiological deformation during movement.^[^ ^13^, ^33^ ^]^ Figure 5b shows the yielding characteristics of various microhydrogel suspensions; Figure 5c provides clustering analysis of this dataset (the full dataset including oscillatory sweep curves can be found in Figure S13, Supporting Information). We find that particle geometry, rather than material composition, determines the key rheological properties. Suspensions with EPI particle geometry indeed clustered tightly together despite their differing constituent materials (CMC, hyaluronic acid, and alginate, Figure 5c). In comparison, spherical particles lack high yield strain. Particle porosity provided elastic strain softening, enabling injectability at low yield stress in high‐stiffness, high‐yield biomaterials.

We finally develop a methodology to modify the EPI meta‐biomaterial for delivery of adherent cells. CMC being strongly cell‐repulsive,^[^ ^48^ ^]^ covalent modification^[^ ^38^, ^49^ ^]^ or polyelectrolyte coating^[^ ^31^ ^]^ can provide cell adhesion motives. Here, we prefer covalent modification, since acid‐based neutralization of the carboxyl groups in the EPI biomaterial indicates a requirement for about 25% of the original negative charge density to maintain a softening transition (Figure 5d). We observe little change in EPI rheology for a mass fraction of 1% or 3% of immobilized collagen I,^[^ ^38^, ^49^ ^]^ although complete loss of the strain softening transition was observed at 10% collagen content (Figure 5e,f). This finding likely reflects saturation of the collagen adsorption capacity of CMC cryogels,^[^ ^38^ ^]^ strongly increasing the friction coefficient (simulation in Figure 5f, and Figure S18, Supporting Information). Since cell adhesion requires less than maximal collagen modification (Figure 5h,i) we obtain a path to EPI meta‐biomaterial functionalization for cell therapy without major change in physical properties. This is illustrated in Figure 5j,k, where OP‐9 cells expressing green fluorescent protein (GFP) were cultured for 24 h on collagen‐modified EPI meta‐biomaterials (3% collagen by weight) prior to injection in subcutaneous space. Albeit preliminary, we noticed a relatively high cell density at 3 weeks in the cell‐loaded EPI meta‐biomaterials (Figure 5j, similar to the 6‐month unseeded homolog shown in Figure 4j). We find the scaffolds to be colonized by both endogenous and GFP‐stained transplanted cells (Figure 5k), indicating at least partial cell survival and a capacity to rapidly recruit native stroma.

## 3 Conclusion

We present the design, synthesis, and testing of an in vivo 3D tissue engineering material based on metamaterial physics.^[^ ^1^, ^2^ ^]^ Numerical simulations translated clinical requirements^[^ ^13^, ^16^, ^19^ ^]^ into a set of engineerable mechanical and geometric parameters. With our EPI biomaterial, we achieved the desired shape‐stable, softening, and yielding behaviors, along with substantial porosity and tissue‐matching mechanics. Given the range of conditions driving pathological soft and functional tissue loss, we anticipate a major impact of our novel metamaterial approach on 3D tissue reconstruction and cell therapy.

The material's reversible softening closely matched adipose tissue over a wide range of strains, producing an injectable with unprecedented in vivo 3D lifting^[^ ^16^ ^]^ and shaping capacity. Potential soft tissue engineering applications could for instance include large volume reconstruction of cheeks, nose, chin, or breast.

More fundamentally, our work provides empirical proof to the conjecture of elastic softening in microgel suspensions.^[^ ^29^, ^30^ ^]^ The key is the rational metamaterial design^[^ ^1^ ^]^ of the EPI microgeometry with its high porosity and particle irregularity. We thus reconcile large‐scale injectability with in vivo stability and stiffness otherwise requiring preformed scaffolds.^[^ ^31^ ^]^ Particle fine‐tuning with precisely determined shapes and structures could open up further avenues such as physically cohesive or even mechanically responsive and bistable injectable metamaterials.

The induction of a native fibrovascular tissue with a low inflammatory response is remarkable in an injectable with dense particles, where predominance of macrophages and giant foreign body cells would be expected.^[^ ^26^, ^50^ ^]^ The exquisite tissue matching^[^ ^14^ ^]^ offered by the engineered softening transition is likely an important element. As substantial biodegradation occurs toward the end of the follow‐up, we ascribe the favorable outcome also to the biocompatibility of both the CMC backbone^[^ ^38^ ^]^ and cross‐linker^[^ ^51^ ^]^ used here. Finally, colonization by a vascularized stroma is a powerful asset for cell therapy.^[^ ^52^, ^53^ ^]^ The metamaterial strategy enables wide customization, including cell adhesion motives. Functionalization for in vivo neural cell delivery^[^ ^54^ ^]^ and bone marrow engineering^[^ ^52^ ^]^ is available, and the low injection force provides room to target firmer glandular or muscular tissues.^[^ ^55^ ^]^ Overall, we believe our approach is well‐suited for a wide range of customized tissue engineering applications in 3D soft tissue reconstruction and regenerative medicine.

## 4 Experimental Section

Full details on the experimental methods are available in the electronic Supporting Information (Methods section). This includes chemicals, detailed procedures, and equipment used. Here, a short summary is provided.

### Simulation

Numerical simulations included elastically and frictionally interacting particles based on the model by Otsuki et al.,^[^ ^29^ ^]^ with rigorous implementation of symmetric stress tensor evaluation.^[^ ^35^, ^56^ ^]^ The possibility of cross‐linking neighboring elements into distinct particles was additionally added. The simulations were written as custom Python code^[^ ^57^ ^]^ and run on the Baobab Cluster at the University of Geneva.

### Statistics

Depending on normality of the data (Shapiro‐Wilks, Royston^[^ ^58^ ^]^ in the bivariate case), parametric or non‐parametric tests were used: for data compatible with normality, *t*‐test, linear regression, and Hotelling test in the bivariate case^[^ ^59^ ^]^ were used; if normality was violated at *p* < 0.05, the corresponding non‐parametric tests were used (Wilcoxon signed rank or Mann–Whitney U, spatial rank testing^[^ ^60^ ^]^ in the bivariate case). Locations were reported as means and errors as single standard deviations. Clustering analysis was performed based on the bivariate Mahalanobis distance between the materials.^[^ ^61^ ^]^

### Biomaterial Synthesis

Cryogel scaffolds were synthesized as reported,^[^ ^31^ ^]^ with minor modifications, including various backbone polysaccharides. Briefly, a CMC/adipic dihydrazide reactive mix was prepared and precooled at 4 °C. Upon addition of the watersoluble carbodiimide 1‐ethyl‐3‐(3‐dimethylaminopropyl)carbodiimide (EDC), the initiated reactive mix was loaded in syringes and allowed to freeze and then polymerize at −20 °C for 24 h. The EPI biomaterial was obtained from bulk scaffolds by controlled forceful extrusion through a 22‐gauge catheter. Collagen‐modified EPI biomaterial was obtained by adapting established procedures.^[^ ^49^ ^]^ Non‐porous control biomaterials were obtained by adapting the synthesis to room temperature conditions, with fragmentation to produce irregular particles and emulsion polymerization to produce spherical ones.

### Particle Morphology and Porosity

Particle morphology and porosity were evaluated for various materials after confocal imaging and thresholding in ImageJ (Li algorithm^[^ ^62^ ^]^ or manually). For the Sephacryl S200 material, autofluorescence was used for imaging; for the other materials, staining with rhodamine 6G was performed. Particle shape was evaluated according to ImageJ's built‐in shape descriptors “roundness” and “solidity,” pore size distribution by fitting maximal circles,^[^ ^63^ ^]^ and particle size from the particle area.

### Cell Adhesion

Cell adhesion was quantified by culturing OP9 cells^[^ ^52^ ^]^ on unmodified and variously collagen‐modified EPI biomaterial (0%, 1%, 3%, and 10% collagen by dry weight, 600 000 cells per mg of dry material). Short‐term (1 h) adhesion was quantified by metabolic activity (Alamar Blue) of the cells remaining with the scaffolds following transfer to a fresh well, long‐term adhesion (5 days) by confocal microscopy after fixation, and staining for actin.

### Rheology, Uniaxial Compression, and Ejection Force

Rheological characterization was performed in oscillatory shear mode (0.2 Hz), on a Haake Rheostress RS100 5 Ncm apparatus, with custom holders (cup, plate‐plate) equipped with roughened surfaces to avoid surface slipping. Rheological master curves were obtained by normalization of the moduli to the storage modulus value *G*′ at the low stress limit.^[^ ^43^ ^]^ Uniaxial compression was performed on Texture Analyzer TA.XT machine, generally at 0.01 mm s^−1^. Ejection force was quantified using a custom syringe holder for the Texture Analyzer TA.XT, or a Mecmesin MultiTest 2.5 dV mechanical test bench for higher forces (the Texture Analyzer TA.XT being limited at about 12 N).

### Animal Experiments

Animal experiments were approved by the Animal Care and Use Committee of the Canton of Vaud, Switzerland (Authorization VD 3063 and VD 3629), and performed on female, adult CD1 (NSG for cell transplantation experiments) mice between 12 and 20 weeks of age. Biomaterial injection into the subcutaneous space was performed on anesthetized animals through a 20G needle; shaping was manual within 20 min of injection. Shape maintenance was assessed externally by caliper measurements and magnetic resonance imaging (MRI) was performed in spin echo mode at 14.1 T with respiratory gating. Histological processing followed standard paraffine embedding procedures after immersion fixation of the implants.

### In Vivo Biodegradability

Biodegradation of EPI was evaluated from the decrease in local CMC concentration in visually identifiable EPI fragments on deparaffinized histological cuts, based on confocal imaging of the uptake of the Rhodamine 6G cation, assuming a Donnan equilibrium.^[^ ^64^ ^]^

### Replication

Unless explicitly stated otherwise, replicated experiments were performed on distinctly prepared biomaterial samples. Concerning numerical reproducibility, the authors found numerical differences on the order of 10^−14^ or less for successive evaluations, in most cases, with the exception of least squares fitting algorithms with more major updates, producing errors on the order of 10^−6^. Hence, all numerical replication errors were small compared to experimental errors, which were more typically in the 10^−2^ to 10^−1^ range.

## Conflict of Interest

A.B. and T.B. declare financial interest in Volumina‐Medical SA, Switzerland. P.B., M.M., and A.B. are currently or were employees of Volumina‐Medical SA. The other authors declare no conflict of interest.

## Author Contributions

A.B., F.B., and C.V. contributed equally to this work. A.B., F.B., C.V., M.G., and T.B. contributed to the design of this study. All authors contributed to the writing and proofing of the manuscript. A.B., M.G., and M.M. carried out the in vivo study, F.B., M.G., A.F., T.B., and A.B. analyzed the in vivo data. J.T. and J.B.‐G. performed the in vitro and in vivo cell‐delivery studies and analyzed the data with the help of F.B., T.B., and O.N. J.B.‐G. developed strategies for improved injectability as well as biomaterial coating. F.B. provided the morphological biomaterial characterization and the design of the figures in the manuscript. C.V., F.B., J.B.‐G., and T.B. performed the mechanical characterization; T.B. wrote the software, ran and analyzed the simulations, F.B. and J.B.‐G. drafted software usage instructions, and P.B. acquired software test data. A.F ensured planning and collaboration for the manuscript. P.B. developed and optimized biomaterial fabrication.

## Supporting information

Supporting Information

Supplemental Video 1

Supplemental Video 2

Supplemental Video 3

Supplemental Video 4

Supplemental Video 5

## Data Availability

Figures  1, 2, 3, 4 and 5, and the figures in the Supporting Information have associated quantitative raw data. The raw data and the scripts to produce the quantitative figures are provided at CodeOcean (Capsule 6934377, https://doi.org/10.24433/CO.6934377.v1 ^[^ ^34^ ^]^); for appropriate versioning the capsule is also included into the main raw data repository https://doi.org/10.5281/zenodo.4936976.^[^ ^65^ ^]^ The CodeOcean capsule^[^ ^34^ ^]^ automatically installs external custom dependencies in the form of Python and R libraries during the build phase. These libraries (Python: particleShear;^[^ ^57^ ^]^ R: textureAnalyzerGels,^[^ ^66^ ^]^ rheologyEvaluation,^[^ ^67^ ^]^ plot.counts,^[^ ^68^ ^]^ and reproducibleCalculationTools^[^ ^69^ ^]^) are available separately for installation and use.^[^ ^57^, ^67^, ^68^, ^69^, ^70^ ^]^ For automation of image treatment in ImageJ, we used a custom plugin for execution of macros tabulated in Excel sheets, also available for download.^[^ ^71^ ^]^ The ImageJ plugin used to evaluate intra‐ and interparticle pore space contribution is also available.^[^ ^72^ ^]^
