# Supplementary material for: An Injectable Meta‐Biomaterial: From Design and Simulation to In Vivo Shaping and Tissue Induction
Source: Adv Mater. 2021 Aug 27;33(41):2102350. doi: 10.1002/adma.202102350 (PMC11469256; doi:10.1002/adma.202102350)
Supplement: Supplementary file 1 — Supporting Information [file ADMA-33-2102350-s005.pdf]

© 2021 Wiley-VCH GmbH

ADVANCED MATERIALS

Supporting Information

for *Adv. Mater.,* DOI: 10.1002/adma.202102350

An Injectable Meta-Biomaterial: From Design and Simulation to In Vivo Shaping and Tissue induction

*Amélie Béduer, Fabien Bonini, Connor A. Verheyen, Martina Genta, Mariana Martins, Joé Brefie-Guth, Josefine Tratwal, Aleksandra Filippova, Patrick Burch, Olaia Naveiras, and Thomas Braschler**

Supporting Information

An Injectable Meta-biomaterial: From Design and Simulation to In-vivo Shaping and Tissue induction

*Amélie Béduer[§], Fabien Bonini[§], Connor Verheyen[§], Martina Genta, Mariana Martins, Joé Bréfie-Guth, Josefine Tratwal, Aleksandra Filippova, Patrick Burch, Olaia Naveiras and Thomas Braschler**

[§] equal contribution
* corresponding author

[1] University of Geneva, Faculty of Medicine, Department of Pathology and Immunology. Rue Michel-Servet 1, CH-1211 Geneva, Switzerland.

[2] Ecole polytechnique fédérale de Lausanne, EPFL, School of Engineering, LMIS4. BM, Station 17, CH-1015 Lausanne.

[3] Volumina-Medical SA, Route de la Corniche 5, CH-1066 Epalinges, Switzerland.

[4] University of Lausanne, Laboratory of regenerative Hematopoiesis, Department of Biomedical Sciences, Rue du Bugnon 27, CH-1011 Lausanne, Switzerland.

[5] CHUV, Hematology Service, Department of Oncology, Rue du Bugnon 46, CH-1011 Lausanne, Switzerland.

[§] These authors contributed equally
[*] Corresponding author: thomas.braschler@unige.ch

Keywords: injectable metamaterial, elastic softening, tissue reconstruction, shaping, vascularization

1. Additional simulation results

1.1. Controls without friction

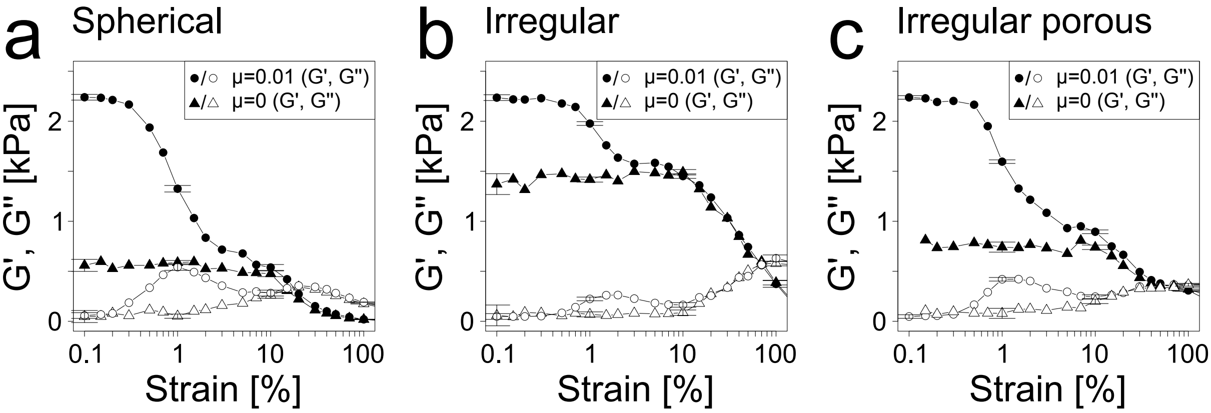

Figure S1. Non-frictional control ensemblies. The figure provides the non-frictional controls for the strain sweeps for spherical, irregular, and irregular porous particles. The frictional ensemblies were all simulated with a material Young modulus E=10kPa, a packing density of $\phi_0 = 1.5$ *, and a friction coefficient of* μ*=0.01. For the spherical particle suspension (a), no permanent crosslinks were present ; for the irregular particles (b), all neighboring spheres within the particles were crosslinked permanently to each other, while for the irregular porous particles (c), a fraction of* χ*=40% of the permanent crosslinks were removed within the particles to emulate porosity. The non-frictional ensemblies use* μ*=0 but otherwise the same parameters as their frictional homologs. The strain sweeps for the frictional case (*μ*=0.01) are also given in the main text, as Figure 2b, d and e.*

Figure S1 compares frictional and non-frictional particle assemblies. The frictional scenarios correspond to the particle suspensions described in the main text (Figure 2b for Figure S1a, Figure 2d for Figure S1b and Figure 2e for Figure S1c). For the non-frictional controls, the strain sweeps were simulated with the same underlying physical parameters, but with a friction coefficient μ=0 instead of μ=0.01 for the frictional case.

Comparison between the oscillatory shear strain sweeps on the frictional and non-frictional ensemblies given in Figure S1 shows that friction has an important contribution to the G' values at low shear strain (<1% strain). At moderate strains (around 1%), we find a softening transition, with a decrease of the G' values to approximately the non-frictional plateau level. At higher strain still (10% and more), yielding takes place and the G' curves drop below the G'' curves. At such high strains, friction is of no importance, at least for the value of μ=0.01 chosen for the frictional ensemblies.

Figure S1a confirms the theoretically known presence of a softening transition in frictional spherical microgel suspensions[1]. Figure S1b and S1c extend the result to irregular, compact and irregular, porous particles. Figure S1 also indicates that the softening transition is accompanied by energy dissipation linked to friction, as there are local maxima in the G'' associated with the softening transition that disappear upon removal of friction.

1.2. Influence of physical model parameters on the oscillatory stress sweep

1.2.1. G' and G'' curves associated with model parameter variation

In the main text (Figure 2g), the influence of 5 physical model parameters on the G' and G'' response in a simulated oscillatory strain sweeps is summarized. The 5 physical parameters are: Young modulus E of the constituent material; packing density of the microspheres ϕ_0; Friction coefficient μ; presence of porosity (modelled by the removal of a fraction χ of permanent crosslinks); and irregular vs. spherical particle shape (modelled by the presence of permanent defining irregular particles vs. total absence of permanent crosslinks).

G' and G'' curves

Figure 2g in the main text succinctly summarizes the observed effects of these 5 factors on features of the stress sweep diagram, defined in Figure 2f. Here, we provide direct visualization of the effect of each parameter. We do so by varying each time a single model parameter, keeping all the others constant.

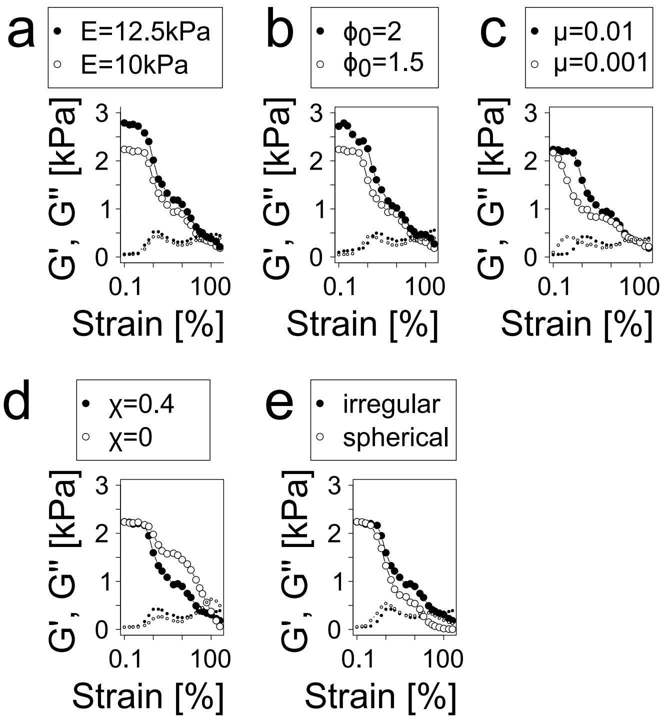

Figure S2. Influence of the model parameters on the elastic storage modulus G' and viscous loss modulus G'' curves in simulated oscillatory strain sweeps. A) Change of Young modulus E B) Change of packing density as expressed by the nominal solid phase volume ϕ_0 . C) Change of friction coefficient μ. D) Change of permanent crosslinking density emulating porosity by removal of a fraction of χ of the permanent crosslinks. E) Spherical or irregular geometry emulated by total removal of the permanent crosslinks for the spherical geometry. In all subfigures of the panel, the reference curve obtained with E=10kPa, $\phi_0 = 1.5$, μ=0.01, χ=0.4 and presence of permanent crosslinks is given, in addition to an additional curve as denoted by the legend.

Figure S2 shows the results. Keeping all other parameters constant, increasing the Young modulus E of the constituent material leads to a proportional increase in the G' value, without change of the characteristic strain values as there is no horizontal shift of the curves (Figure

S2a). Increasing the packing density affects the G' response curve in several ways (Figure S2b). The low-strain plateau G' increases and the softening transition is shifted to higher strains. There is also a minor increase in yield strength, statistically significant as indicated in Figure 2g in the main text. The friction coefficient specifically affects the strain at which the softening transition occurs (Figure S2c); Introducing porosity as emulated by the removal or not of 40% of the permanent crosslinks has a specific effect on the soft G' plateau at strains above the softening transition (Figure S2d). Total removal of the crosslink to obtain the known condition of spherical microspheres[1] lowers the soft G' plateau even further, but it also greatly reduces yield strain.

Statistical evaluation

As outlined in the online methods section, we used a bootstrapping method to evaluate the influence of each of the parameters on the different characteristics of the G' and G" curves (main text, scheme in Figure 2f, significance results in Figure 2g). The P values underpinning Figure 2g are given in Table S7, items 5-25.

1.2.2. The particular case of friction

The tendencies visible in Figure S2 correspond to, and confirm, the statistical overview given in Figure 2g in the main text. The case of the friction coefficient μ merits however additional discussion. For the relatively low values shown in Figure S2c (comparison of μ=0.001 to μ=0.01), the main effect of friction is to shift the softening transition. The softening strain is indeed on the same order of magnitude as the friction coefficient. In this scenario, there is no statically significant effect of the friction coefficient on the yielding transition (Figure 2g in the main text).

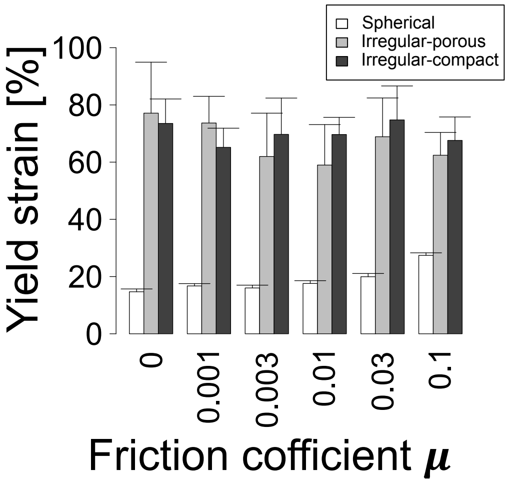

Figure S3. Influence of the friction coefficient μ on the yield strain. Other than the particular values indicated in the figure, the common general model parameters were: Young modulus of the constituent material E=10kPa, packing density $\phi_0 = 1.5$ *, and removal of a fraction of* χ=0.4 *of the permanent crosslinks to emulate the porous particles.*

In the case of large friction coefficients μ, the distinction between softening and yielding is known to disappear for spherical microgel suspensions [1]. One may therefore expect that

sufficiently important friction would help stabilize the ensemble by displacing to higher strain values the merged softening-yield transition. Thus for values of μ approaching the geometrical yield strain in the absence of friction, one would expect an increasing influence of the friction coefficient on the yield strain. Figure S3 shows this behaviour for the simulated spherical microgel suspension: the geometric yield strain in the absence of friction (μ=0) for the spherical microgel suspension was here evaluated to 0.15+/-0.01; the yield strain increases visibly with increasing friction coefficient (Figure S3). Linear regression confirms a dependency of the yield strain on the friction coefficient with a coefficient near 1 (yield strain = 0.16+1.17*μ by linear regression on the average values in Figure S3, $P=3.5*10^{-17}$ by bootstrapping, item 17 in Table S7).

For the irregular particle suspensions, whether compact or heterogeneously crosslinked to emulate porosity, the yield strain is found to be much higher (on the order of about 0.7) and no significant dependency on the friction coefficient could be found.

In Figure 2g in the main text, we therefore indicate that in some situations, such as shown in Figure S2c above, there is little dependency of the yield strain on friction, whereas in others, such as the spherical microgel suspensions simulated in S3 at higher friction coefficients, a significant dependency does arise.

1.2.3. Packing density

Additional detailed information on the influence of packing density on the model output is provided in the CodeOcean capsule[2] (https://doi.org/10.24433/CO.6934377.v1, folder "Figures_CodeOcean_only" in the Results section, Figures C1, C2, description in CodeOceanOnlyResults.pdf).

2. Oscillatory shear rheology of bulk scaffold samples

A major design criterion for the EPI biomaterial is that the elastic storage modulus G', as measured by oscillatory shear rheology at low strain, should match the one of the tissue to be replaced or augmented. Based on literature data on the rheological response of adipose tissue,[3] we set a desirable target range of 2-3kPa for the low strain elastic storage modulus. As the elastic properties of cryogels are primarily a strong function of the polymer content,[4] we used adjustment of the total carboxymethylcellulose content in the reaction mixture to achieve the target G'. Here, we provide the data confirming that indeed, the final chosen recipe has a mean low-strain G' in the 2-3kPa range.

The elastic storage modulus G' and the viscous loss modulus G'' characterize the linear viscoelastic response of a material under a given sinusoidal shear stimulation.[5] They are obtained via demodulation of the time-variable force with regard to the sinusoidal deformation imposed on a sample;[5] force in phase with deformation characterizes the elastic response and gives rise to the G' component; force in phase with the deformation rate (and thus 90° out of phase with the deformation) is linked to viscous deformation and therefore gives rise to the G'' component in demodulation.[5]

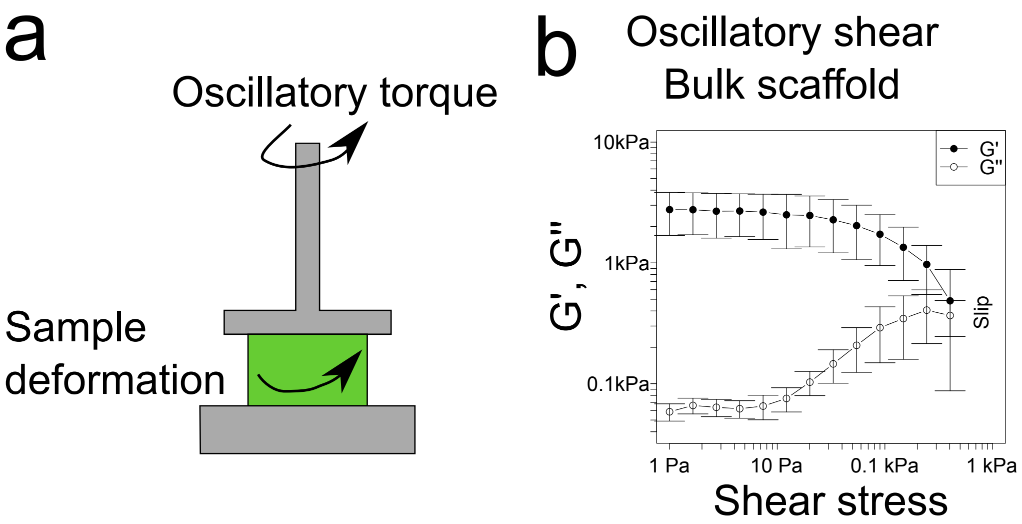

Figure S4. Oscillatory rheometry of bulk scaffolds. a) Bulk scaffold samples (cylinders of 2cm diameter and ca. 6mm height) were clamped between roughened chuck surfaces of a Haake Rheostress 100 rheometer and exposed to ramps of increasing oscillatory shear stress at a constant frequency of 0.2Hz. At each imposed shear stress value, the elastic storage modulus G' and the elastic loss modulus G'' were obtained (the normalization and demodulation is performed automatically by the controller software Rheowin) b) Elastic storage modulus G' and viscous loss modulus G'' as a function of increasing applied shear stress (torque). At high shear loads (>400Pa), slippage at the interface between the chucks and scaffolds occurs, with local scaffold destruction.

We carry out oscillatory shear rheology of the scaffold samples on a Haake Rheostress 100 instrument, using a 20mm diameter plate-plate geometry (schematically shown in Figure S4). To rule out slipping effects on the chuck surfaces, we fixed a cleaning cloth that has small protruding metal filaments to the chuck and plate surfaces. The filaments become anchored in the pore structure of the scaffold, and manual movement of scaffolds thus hooked to the chuck and plate results in local scaffold destruction rather than in slipping movement.

The instrument offers controlled deformation and controlled stress modes; we find it generally to be both more precise and flexible in controlled stress mode. Demodulation of the mechanical response is carried out automatically by the Rheowin software supplied with the Rheometer. The scaffold samples pose some difficulties in rheological characterization. For a measurement apparatus primarily designed to characterize flow behavior of viscous or viscoelastic fluids, the cryogel scaffolds are in fact extremely elastic (viscous loss modulus G'' only a few percent of the elastic modulus G' at the lowest strains, see Figure S4b). As a result, the composite system of elastic sample and nearly friction-less, but massive chuck has a pronounced rotary oscillation peak with a sharply defined eigen frequency. With the chuck and sample dimensions we use, the resonance frequency is typically around a few Hz. In order to avoid undue excitation of such resonances, we set the excitation frequency much lower, at 0.2Hz.

Figure S4b shows the averaged G' and G'' response curve in oscillatory shear rheology with increasing applied shear forces. On the low shear plateau (shear force between 1Pa and 5Pa), we find G'=2.4kPa +/- 0.9kPa and G''=0.064 +/- 0.013 kPa. As the shear force increases, G' starts to decrease and G'' starts to increase; in analogy to numerical and experimental results on the porous particles, this is probably attributable to progressive overcoming of internal

friction within the porous bulk scaffolds. At shear forces exceeding 400Pa, we observe onset of progressively more rapid chuck rotation and sample destruction within a few seconds. Interestingly, the initial sample destruction plane is located not at one of the chuck surfaces as one would expect for slippage, but above the metal filaments, indicating that the weakest point is not at the actual chuck surfaces nor the chuck-cryogel interaction zone, but within the cryogel itself. This in turn suggests that the G' and G'' curves shown in Figure S4 do indeed reflect scaffold properties up to the point of sample destruction by the imposed shear.

In any case, the mean low-strain G' value of 2.4kPa indicates that on average, the bulk scaffolds meet the design criteria for a G' value between 2-3kPa. As we generally pool at least 10 bulk scaffold samples when producing the EPI biomaterial scaffold by fragmentation, we can indeed state the average bulk material from which the EPI biomaterial is produced matches the 2-3kPa G' range set forth.

3. Particle size and geometry

3.1. Particle size

3.1.1. Image acquisition

We analyzed the particle size distribution by confocal imaging. For this, we stained the particles in Rhodamine 6G hydrochloride solution (5 µm/mL in deionized water), followed by thorough rinsing in deionized water to remove free dye. Particle images on a large number of particles were acquired by placing a dilute particle suspension into an observation chamber (defined by spacers between coverslides). After sedimentation of the particles to the floor of the observation chambers, a confocal mosaic was acquired at about 50 µm above the lower coverslide (LSM800, 5x objective).

3.1.2. Image treatment

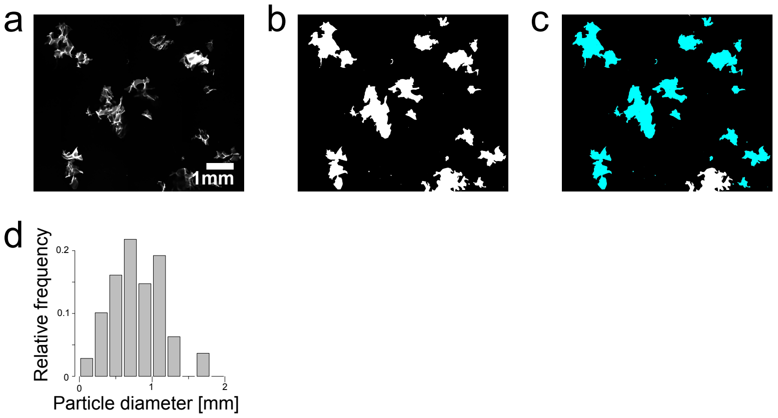

Figure S5. Evaluation of particle size by confocal imaging. a) Mosaics of confocal images at low resolution (5x objective, total area typically about 2.5cm x 1cm) were acquired on a Zeiss LSM800 and assembled. For better visibility, only a region of interest is shown here. b) Manual thresholding followed by hole-filling is used to define the particle mask. c) Particles

are identified and measured, with exclusion of particles touching the image boundaries. d) Distribution of the particle diameter. The histogram is area-weighted ; each category represents an interval of 0.2mm of particle diameter.

All image treatment was carried out in Fiji (ImageJ).[6] The mosaics were assembled into a single large image with the known image positions. In some cases, the mosaics were cropped to avoid interference with fluorescence from the spacers. A limited area of such a mosaic is displayed in Figure S5a.

The assembled mosaic images were thresholded manually to distinguish particles from background. Holes in the resulting binary were filled using the "Fill Holes" functionality implemented in Fiji[6] (i.e. Process -> Binary -> Fill Holes); Figure S5b shows the resulting particle mask, for the same area as Figure S5a. Particles were then analyzed using the built-in particle analysis functionality of Fiji[6] (i.e. Analyze -> Analyze Particles …). A minimal circularity of 0.1 was required, along with a minimal area of 10μm^2; particles on the image boundaries were excluded from the analysis. Figure S5c shows the particle analysis for the region defined by Figure S5a. For the 4 mosaics analyzed, a total of 663 particles were detected, and for each particle the area was measured. From the areas, an equivalent diameter was calculated according to

$$d = 2\sqrt{\frac{A}{\pi}} \qquad \text{eq. S1}$$

As there are numerous very small particles, we evaluate the particle size distributions, mean and standard deviation by using particle area as the weighting function. This accounts for the fact that larger particles encompass a larger mass and thus better reflects the average particle size associated with most of the mass of the EPI biomaterial.

3.1.3. Particle size distribution

The particle size distribution was evaluated from confocal images by thresholding, particle identification and evaluation of the equivalent diameter (Figure S5, eq. S1). The distribution of the particle diameters is relatively broad (Figure S5d, area-weighted). For a mean particle diameter of 805 µm, we indeed find a standard deviation of 363 µm (area-weighted evaluation).

3.2. Particle geometry of control particles compared to EPI

In order to quantitatively characterize the difference in particle shape in microgel suspensions, we analyzed a series of commercial products (Cytodex 1, Cultisphere S, Juvéderm Voluma "HA control", Sephacryl S200) along with EPI biomaterial (CMC and aginate-based), as well as irregular and spherical control microgels synthesized from CMC and alginate based on the same crosslinking chemistry as the EPI biomaterial. We also included analysis of literature-based images of Cultisphere S as an example of porous, but roughly spherical particles[7].

3.2.1. Confocal imaging

Particles were imaged using a confocal microscope (Zeiss LSM700 or LSM800, details given in the main text and the extended methods given in the extended methods at the end of this document). Alginate-based particles and Cytodex 1 were labelled with 6-aminofluorescence. The CMC-based particles and HA irregular were labelled with a rhodamine 6G solution as outlined in the text and the extended methods.

3.2.2. Image treatment

Image treatment for shape analysis was performed as outlined in the main text and the extended methods section (chapter 13 of this document). Briefly, after automated (and occasionally manually re-adjusted thresholding for obvious errors), the “Shape descriptor” ImageJ plugin[8] was used to obtain “roundness” and “solidity”. Roundness was expected to be associated with the distinction between spherical and irregular particles, whereas solidity was expected to be a measure for porosity.

3.2.3. Results

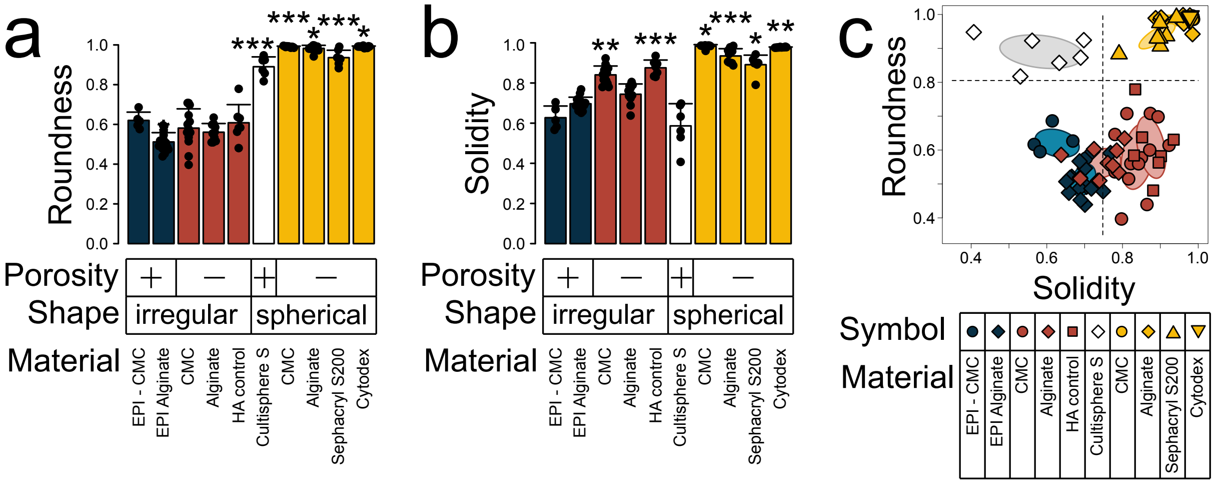

Figure S6. Quantification of “Roundness” and “Solidity” parameters[8] to characterize particle shape irregularity and porosity. a) A high roundness value is associated with known round particle shape. b)Solidity is highest for non-porous spherical particles, intermediate for irregular particles and low for porous particles. Statistical testing against the EPI base material (EPI – CMC), items 84 and 85 in Table S7. c) 2D scatterplot with threshold values. The threshold values (dotted) are estimated by equal false-identification rates for known spherical vs. irregular particles (parameter roundness) respectively know porous vs. non-porous particles (parameter solidity), items 87 and 88 in Table S7.

Figure S6 shows the results of the analysis of roundness and solidity for particles from the 10 chosen commercial and specifically synthesized materials in comparison. Fig. S6a indicates that roundness is robust measure of particle irregularity[8] regardless of particle porosity. The best estimate for the threshold between known irregular and spherical particles is situated at a roundness value 0.81 (item 84 in Table S7, with equal estimated false identification rates for the two populations). Solidity is low for porous particles, but is partially affected also by particle irregularity as shown in Figure S6b. It is nevertheless possible to identify a threshold with equal estimated false identification rate, at a solidity value of 0.75 (item 85 in Table S7). These threshold values are reported as dotted lines in Figure S6c, and also in Figures 3d and 3e in the main text.

4. Porosity and pore size

4.1. Pore fraction and pore size

4.1.1. Hydration state

Poro-elastic media are defined by the simultaneous presence of a mobile pore fluid phase and an elastically deformable wall phase.[9] By this definition, the elastic porous injectable (EPI) biomaterial, the spherical reference microgel suspension Sephacryl S200 are both examples of poro-elastic media. Poro-elastic media are able to accommodate different amounts of fluid due to elastic deformation of the surrounding wall phase. In the EPI biomaterial and the Sephacryl S200 reference material, the pore fluid is aqueous and so the pore space characteristics need to be related to the hydration state.

In the porous hydrogel media considered here, it is convenient to express the hydration state by the total concentration *c* of polymeric material; drier formulations have a higher content of polymeric material. The total polymer concentration is easily determined experimentally by extensive washing with deionized water to remove mobile solutes, followed by drying to constant weight:

$$c = \frac{m_{\mathrm{dry}}}{V} \qquad \text{eq. S2}$$

where m_{dry} is the mass present after drying to constant weight, and V is the original volume. For practical purposes, we generally obtain the hydrated volume V by weighing, assuming a density of $1000 kg/m^3$. In the following analysis, we report pore space characteristics such as total pore fraction or mean pore size as a function of dry weight concentration *c*.

4.1.2. Pore fraction

The remaining pore fraction in microgel suspensions is complicated by the fact that with increasing dehydration, not only the pore space, but also the gel material itself is compressed.[10] This double compression mechanism has been evaluated theoretically based on the movement of counter ions[10]. The following expression was obtained (eq. 5 in [10]):

$$\left(\frac{\phi}{\phi_0}\right)^{2/3} = 1 - \frac{\Gamma}{1-\Gamma}\frac{\phi}{1-\phi} \qquad \text{eq. S3}$$

where Γ is an osmotic compression coefficient. ϕ is the volume fraction occupied by the wall material, and ϕ_0 the wall fraction that would be occupied if there were no wall material compression. In the limit of very high hydration (low polymer content),$\phi \approx \phi_0$, so that ϕ_0 can also be interpreted as the volume fraction occupied by the wall material in dilute suspensions.

In both the EPI biomaterial and the Sephacryl S200 reference microgel suspension, the wall material is itself a hydrogel, characterized by a local polymer concentration c_{wall}. In dilute suspensions, c_{wall} is largely superior to the overall polymer concentration *c*, and tends to its limiting value $c_{wall,0}$. Hence, using the limiting wall concentration $c_{wall,0}$, eq. S3 becomes:

$$\left(\frac{\phi}{c/c_{\text{wall,0}}}\right)^{2/3} = 1 - \frac{\Gamma}{1-\Gamma}\frac{\phi}{1-\phi} \qquad \text{eq. S4}$$

Further, since the microgel suspensions are composed of wall and pore space (i.e. $\phi + \phi_{\text{pore}} = 1$), we obtain an expression for the pore fraction:

$$\left(\frac{1-\phi_{\text{pore}}}{c/c_{\text{wall,0}}}\right)^{2/3} = 1 - \frac{\Gamma}{1-\Gamma} \cdot \frac{1-\phi_{\text{pore}}}{\phi_{\text{pore}}} \qquad \text{eq. S5}$$

In the low concentration limit, the right-hand side of eq. S5 approaches 1, and we have the linear relation $\phi_{\text{pore}} = 1 - c/c_{\text{wall,0}}$. Generally, however, eq. S5 needs to be solved numerically for ϕ_{pore} as a function of the polymer concentration c. To obtain the values of $c_{\text{wall,0}}$ and Γ, we perform least squares fitting of the model on the experimentally acquired ϕ_{pore} data.

4.1.3. Pore diameter

For modeling the pore diameter, we consider an elementary spherical pore of diameter d_{pore} lined by the wall material of characteristic thickness D. In this case, the pore fraction is given by:

$$\phi_{\text{pore}} = \left(\frac{d_{\text{pore}}}{d_{\text{pore}}+D}\right)^{n} \qquad \text{eq. S6}$$

where n is a dimensionality parameter depending on the pore geometry. By solving for d_{pore}:

$$d_{\text{pore}} = D\,\frac{{\phi_{\text{pore}}}^{1/n}}{1-{\phi_{\text{pore}}}^{1/n}} \qquad \text{eq. S7}$$

The parameter *n* and *D* can be obtained by least squares fitting from empirical data.

4.1.4. Image acquisition

We analyze the geometrical pore space characteristics by confocal imaging on samples with known dry polymer weight concentration. For obtaining sufficient fluorescence from the EPI biomaterial, we stained it by using Rhodamine 6G hydrochloride solution (5 microgram/mL in deionized water), followed by thorough rinsing in deionized water to remove free dye, and readjustment to known dry weight. The Sephacryl S200 reference material is sufficiently autofluorescent by excitation in the UV and emission in the blue to obviate the need for staining.

We acquired images at moderately high resolution (20x) for this analysis, using a Zeiss LSM800 confocal microscope.

4.1.5. Image treatment

From confocal imaging, the pore fraction is obtained by manual thresholding to outline the walls (bright) as compared to the pore space (dark). The pore fraction is then estimated by quantifying the number of bright pixels as compared to the total number of pixels.

The pore diameter is evaluated based on the maximal sphere fitting algorithm implemented by Münch et al.[11] Details on the usage of this plugin can be found on CodeOcean[2] (https://doi.org/10.24433/CO.6934377.v1, folder "Figures_CodeOcean_only" in the Results section, Figure C3, CodeOceanOnlyResults.pdf).

4.2. Results

4.2.1. Pore fraction

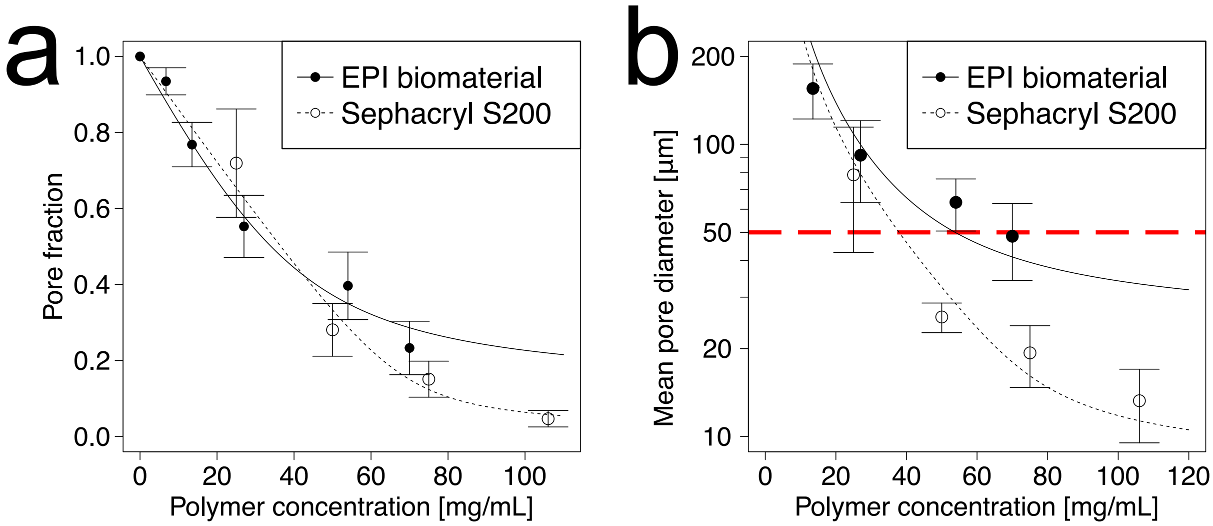

Figure S7. Pore fraction and pore diameter as a function of polymer concentration. EPI biomaterial (stained with 5 microgram/mL rhodamine 6G) and Sephacryl S200 (unstained) were suspended to known concentrations of polymer (assessed by drying to constant weight) in deionized water. The resulting suspensions or pastes were analyzed by confocal microscopy. a) From this the pore fraction was determined as the relative number of dark pixels compared to the total number of pixels after manual thresholding to distinguish hydrogel material from pore space. The theoretical lines correspond to best fits of the pore fraction as evaluated numerically from eq. S5. The best fit values for the osmotic compression coefficient Γ and the relaxed wall polymer concentration $c_{wall,0}$ are given in Table S1. b) Mean pore diameter for the EPI biomaterial in comparison with Sephacryl S200. The theoretical lines are obtained from eq. S7 with best fit parameters D and n for each material (see Table S2), using the theoretical porosity curves shown in Figure S7a. The red line indicates the 50µm minimum pore size limit generally accepted as a requirement for implant vascularization.[12, 13]

Figure S7a shows the pore fraction as evaluated from confocal imaging. The EPI biomaterial samples were stained with rhodamine 6G, whereas the Sephacryl S200 reference material was sufficiently autofluorescent to avoid the need for staining. By thresholding, we determined the fraction of pixels belonging to the solid hydrogel phase as compared to the free pore space.

We performed the measurements at various hydration states as quantified by the polymer concentration. From Figure S7a, we conclude that regarding the relation between total pore fraction and hydration level, the EPI biomaterial and the Sephacryl S200 reference material are qualitatively similar. Both show first a linear decrease of pore fraction with increasing polymer concentration, followed by leveling off with higher polymer concentrations.

Material	Γ	$c_{wall,0}$
EPI biomaterial	0.11	56mg/mL
Sephacryl S200	0.016	72mg/mL

Table S1. Best fit constants for the pore fraction model. Eq. S5 was used to determine the theoretical pore fraction at various polymer concentration. By least squares fitting against the data shown in Figure S7a, the osmotic compression constant Γ and the relaxed wall polymer concentration coefficient $c_{wall,0}$ were determined. The values shown are the best fit values, and correspond to the theoretical curves in Figure S7a.

We fitted the pore fraction model given by eq. S5 (from [10]) to the curves in Figure S7a, for the both the EPI biomaterial and the Sephacryl S200 reference material. In both cases, the theoretical curves capture the overall relations rather well. The fitting constants (Table S1) suggests similar intrinsic polymer concentrations of the hydrogel phase ($c_{wall,0}$), but a more pronounced wall hydrogel phase compressibility Γ for the EPI biomaterial than the Sephacryl reference material. As can be seen from Figure S7a, this leads to a better maintenance of residual pore fraction at higher polymer concentration. This could be due to the spongy structure of the EPI biomaterial, preventing completion of pore closure due to the irregular structure.

In any case, the fitting constants in Table S1 along with numerical evaluation of eq. S5 for the pore fraction allow a theoretical estimate of the pore fraction at a wide range of polymer concentrations for both materials.

4.2.2. Pore diameter

Next, we analyzed the pore diameter from the set of confocal images underpinning the pore fraction evaluation, according to the image analysis method outlined in Figure 1. The results are shown in Figure S7b. This analysis shows a clear difference between the two materials: particularly at large polymer concentrations, the pores in the EPI biomaterial are larger than in the Sephacryl S200 reference material. The theoretical lines were obtained by estimating the porosity from eq. S5 with coefficients given in Table S1, and then using eq. S7 to obtain an estimate of the pore size. Best fit parameters were determined by least squares fitting with the logarithmic y-scale shown in Figure S7b, and are given in Table S2.

Material	n	D
EPI biomaterial	6.56	8.65 µm
Sephacryl S200	9.01	4.21 µm

Table S2. Best fit parameters for the pore size model. The parameters were obtained by least-squares fitting of the mean measured pore sizes (Figure S7b) by eq. S7, where the pore fraction was in turn obtained by eq. S5 with the coefficients provided in Table S1.

For both formulations, the dimensionality parameter *n* is found to be substantial above what would be expected from isometric compression of spherical (n=3), cylindrical (n=2) or slit-like pores (n=1). This indicates that larger pores subsist at high polymer concentration than if the structures were merely isometrically compressed, meaning that privileged open sites remain. For the Sephacryl S200 material, which is a suspension of spherical microgel particles, these privileged sites are easily identified: they are the interstitial spaces between adjacent spheres, theses pores can only be completely closed with substantial compression of the spheres themselves. For the EPI biomaterial, closer inspection of the confocal images reveals two types of protected sites: near joints of branches of the cryogel, and, similarly to the Sephacryl S200 material, the interstices near contact points due to the mismatch in local shape.

For implant vascularization in-vivo, the pore size should be larger than about 50μm.[12, 13] Using the parameters given in Table S1 and Table S2, we can estimate a maximum allowable polymer concentration compatible with vascularization. For this, we note that the minimal pore fraction can be directly estimated from eq. S6 with the parameters given in Table S2. From the minimal pore fraction, the maximal polymer concentration can then be estimated by solving eq. S5 for the associated concentration :

$$c = c_{\mathrm{wall},0} \cdot \left(1 - \phi_{\mathrm{pore}}\right) \cdot \left(1 - \frac{\Gamma}{1-\Gamma} \frac{1-\phi_{\mathrm{pore}}}{\phi_{\mathrm{pore}}}\right)^{-3/2} \qquad \text{eq. S8}$$

The resulting minimal pore fraction and associated maximal polymer concentrations are shown for the two materials in Table S3.

Material	*Minimal pore size*	*Minimal pore fraction (eq. S6)*	*Maximal polymer concentration*
EPI biomaterial	50 μm	35%	54mg/mL
Sephacryl S200		48%	38mg/mL

Table S3. Estimation of minimal pore fraction and maximal polymer concentration for a minimal pore size of 50μm minimum pore for implant vascularization.[12, 13]

4.3. Porosity internal to particles and between particles

In a system of porous particles, the pore space will be formed both by internal pore space within particles and by pores arising at the interstitial space between two or several particles. To distinguish the types of pore spaces is not trivial; in a system with interconnected pores as found in the EPI biomaterial, this means attributing distinct regions of a largely connected overall pore space to inter- and intra-particle fractions.

Even if conceptually non-trivial, it is nevertheless of major interest to distinguish and characterize inter- and intraparticle porosity within the multiparticle system making up the EPI biomaterial. Indeed, if the overall pore characteristics and namely pore size is similar in the intra- and inter-particle space, one can expect EPI biomaterial implants to induce colonization similar to an implanted intact scaffold. On the contrary, if one of the two pore space were to offer only pores of a size below the colonization and vascularization limit[12, 13], then we would expect colonization to follow the larger pore space type, akin to the very

limited colonization occurring in the HA control material (Figure 4l in the main text). The relative fraction of inter- and intra-particle space may also be of interest in some applications, for instance, in cell transplantation, as intra-particle pore space would probably better be protected from mechanical stress than interparticle pore space.

4.3.1. Methods

Intraparticle pore space fraction

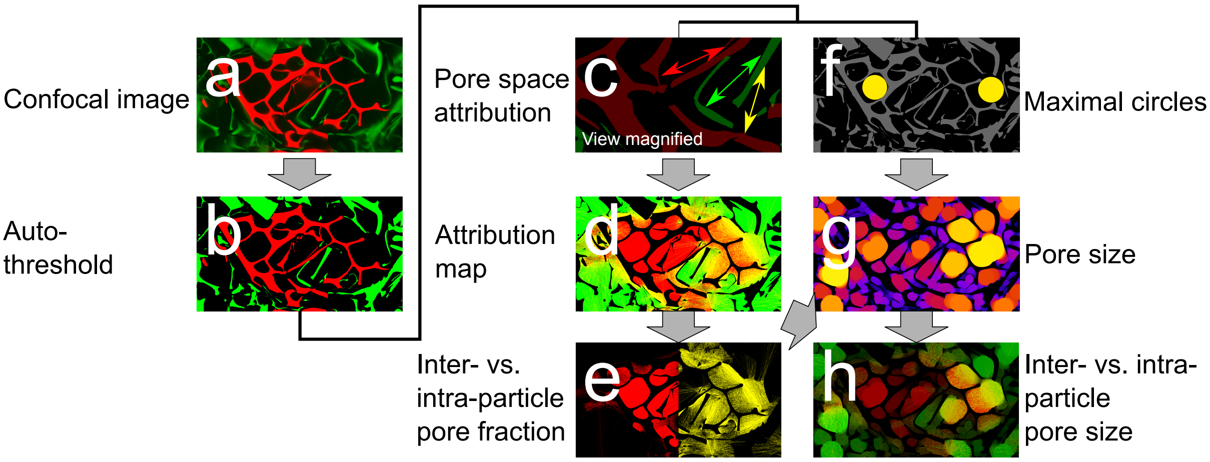

Figure S8. Pore space attribution to the intra- and inter-particle pore space and pore size evaluation per pore space compartment. a) confocal image of minority (here, red) particles embedded in majority particles (here, green). b) automated thresholding in each of the two color channels. c) pore space attribution by random segments. This gives two types of intra-color segments (red and green only segments where both end touch the same color wall) and one mixed type (different color walls at the end of the pore segment). d) Averaging the information from many (typically, 10'000) intersection lines yields a pore space attribution map. e) This indicates an attribution probability for each channel (red, green, yellow, here only red and yellow of interest since red is the minority color). f) Pore size evaluation by fitting maximal local circles (xlib plugin[11]) yield g) a local pore size map. h) For evaluation of the pore size in the different channels, the pore size attribution (color-channels) are multiplied pixel-per-pixel with the pore size image. From the channels of such mixed images, the pore size specific to each compartment is evaluated by eq. S10 and eq. S11.

Figure S8 shows the method used for assigning pore space to intra-particle and inter-particle fractions in confocal pictures. We acquired confocal pictures at moderately high resolution (20x objective) in a mixture of red and green fluorescent particles (red: rhodamine-B-isothiocyanate RITC labelling by pre-reaction of the adipic dihydrazide with a small amount of RITC prior to the cryogel synthesis; green: inclusion of a small amount of aminofluorescein into the cryogel reaction mixture for covalent incorporation[4].) Useful mixture imply an about 10:1 ratio of either green:red or red:green particles, judged by mass; all mixtures were adjusted to 27mg/mL final polymer concentration. We acquired confocal pictures of minority particles in these mixtures, along with some of their surrounding (Figure S8a). The rationale behind this approach is that for minority particles, pore space bounded by the minority color only is most likely intra-particle pore space due to the low chance of spatial contact between the minority particles, whereas mixed pore space is characterized by bounding by the two colors. From the confocal pictures, we defined the colored region by

autothresholding (Figure S8b). We implemented a pore space attribution algorithm[14] as an ImageJ plugin considering pore space segments of random straight lines laid across the image (Figure S8c), with color attribution depending on the bounding wall segments (homogeneous red or green, or mixed if the bounding walls of the segment are of the two colors). By evaluating many (typically, 10'000 or more) lines, one obtains an overall attribution map of the pore space (Figure S8d). From this general attribution map, the intra-particle space can be identified approximately as the minority pore space, while the interparticle pore surrounding the minority particle is defined by the mixed pore space (Figure S8e). For evaluation of the intra-particle pore space fraction, we further note that the mixed space is divided homogeneously between the two colors and so ultimately, the intra-particle pore space fraction can be defined as:

$$\text{Intra-particle pore fraction} = \frac{\langle \text{Intensity minority attribution} \rangle}{\langle \text{Intensity minority attribution} \rangle + \langle \text{Intensity mixed attribution} \rangle / 2} \quad \text{eq. S9}$$

Of note, we expect the intra-particle pore space fraction to be a strong function of particle size. In the extreme, particles smaller than the pore size will typically not display any sizable intra-particle pore space, while larger particles are expected to have a higher volume:surface ratio and so display more intra-particle pore space.

Pore diameter in the intra-particle and inter-particle pore space

To assess the pore diameter in the inter- and intra-particle pore space, we combined the local pore-size determination by B. Münch et al.[11] with the attribution methods outlined above (Figure S8a to Figure S8e). Indeed, by fitting maximal spheres[11] (here, in 2D pictures, this implies maximal circles, Figure S8f) one obtains a local pore size map [11] (Figure S8g). By using the pore space attribution intensities (Figure S8e) as weights for weighted averaging, one can the obtain an estimate of the average pore diameter in the intra- and inter-particle space:

$$d_{\text{pore, intra-particle}} = \frac{\langle \langle \text{Intensity minority attribution} \rangle \cdot (d_{\text{pore}}) \rangle}{\langle \text{Intensity minority attribution} \rangle} \quad \text{eq. S10}$$

respectively

$$d_{\text{pore, inter-particle}} = \frac{\langle \langle \text{Intensity mixed attribution} \rangle \cdot (d_{\text{pore}}) \rangle}{\langle \text{Intensity mixed attribution} \rangle} \quad \text{eq. S11}$$

In both eq. S10 and eq. S11, the multiplication in the numerator is understood to be pixel-wise; a colored illustration of this principle is provided in Figure S8-h, where the differently colored channels portray the pixel-wise product of attribution intensity (color) and pore size-intensity product.

Pore diameter and particle size

One can anticipate that the particularly the intra-particle pore fraction would depend greatly on the particle size. For this reason, we complemented our pore space analysis with information on the size of the minority particles at hand, determined as before (Figure S5, eq. S1).

To a first approximation, one would indeed expect the intra-particle fraction to depend on the volume-to-surface ratio. Expressed for a core-shell model, with a core radius r and a constant shell thickness s, this would be in 2D:

$$\text{Intra-particle pore fraction} = \frac{r^2}{(r+s)^2} \qquad \text{eq. S12}$$

Probably due to the extrusion procedure used in fabricating the particles, we notice that large particles typically appear quite irregular in confocal images and seem to consist of irregularly connected "domains" with a maximum size substantially below the equivalent radius calculated from the area (eq. S1).

To account for this irregularity, one can refine eq. S13:

$$\text{Intra-particle pore fraction} = \frac{r_*^2}{(r_*+s)^2}, \text{ with } \frac{1}{r_*} = \frac{1}{r} + \frac{1}{r_f} \qquad \text{eq. S13}$$

where r_* is the effective "domain" radius, and r_f is an upper limit imposed by the fracture process. In curve fitting, eq. S12 has the inter-particle shell thickness s as its sole free parameter, whereas eq. S12 is a two-parameter model with both the shell thickness s and the maximal domain radius r_f as free parameters to be adjusted. There is also a qualititave difference at the limit of very large particles: for very large r, the simple core-shell model (eq. S12) indicates that intra-particle space should become fully dominant, since $\lim_{r\to\infty} \frac{r^2}{(r+s)^2} = 1$. On the contrary, the domain model predicts convergence of the intra-particle pore fraction to a finite limit, possibly substantially below 1, since $\lim_{r\to\infty} \frac{r_*^2}{(r_*+s)^2} = \left(\frac{r_f}{r_f+s}\right)^2 < 1$.

4.3.2. Results

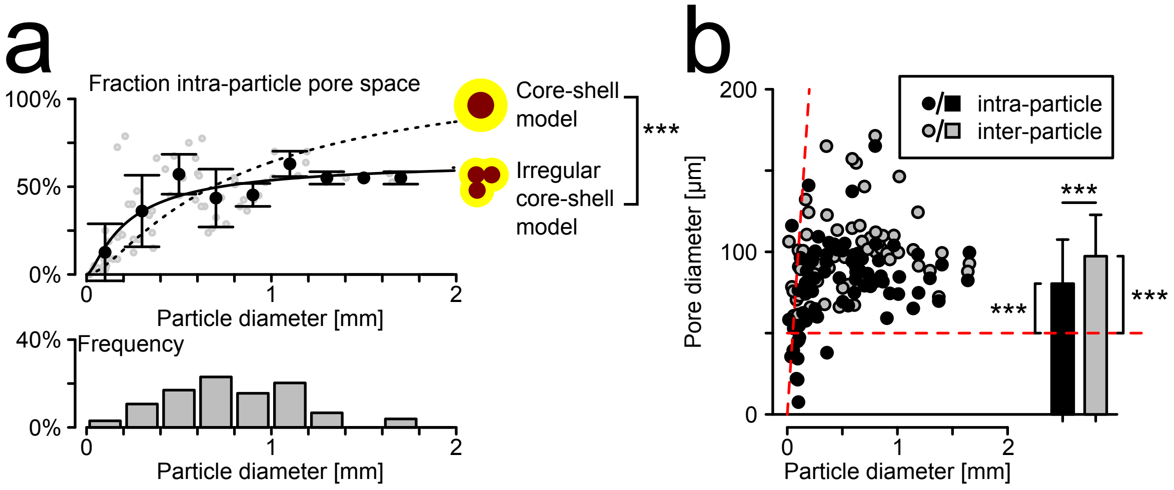

Figure S9. Intra- and inter-particle pore space characteristics. a) Estimated fraction of intra-particle pore space as a function of particle size. The grey dots represent individually assessed particles, the black dots are averages per particle diameter category. The core-shell model is given by eq. S12 with least squares fitting for the value of s, whereas the irregular

core-shell model corresponds to eq. S13 with both s and the limiting domain radius r_f being fitted. The particle size distribution shown is from Figure S5d. b) Pore size in the intra- and inter-particle pore space as a function of particle size. Red horizontal line: 50µm threshold for efficient vascularization and colonization.[12, 13] Red ascending line : Equality line for pore size and particle size. The histogram shows the number averages for the mean intra- and inter-particle pore size associated with the particles evaluated. Statistical tests against theoretical limit of 50µm[12, 13] (Wilcoxon, items 81 and 82 in Table S7 and paired t-test for comparison between intra- and interparticle pore size, item 83).

Figure S9 show the result of the analysis of the intra- and inter-particle pore space. Fig S9a shows that the fraction of intra-particle vs. total pore space increases with increasing particle size, before levelling off at about 50%. This behavior is best captured by the irregular "domain" model (eq. S13; fit significantly better than with the simple core-shell model given by eq. S12, $P=4.1*10^{-8}$, anova between models, item 78 in Table S7). The best fit parameters are: r_f=163µm for the limiting domain radius and s=66µm for the interaction shell width (item 79 in Table S7). These values are realistic considering the physics of the particle assemblies: the limiting domain radius r_f corresponds about to the radius of the catheter used for fragmentation during particle synthesis, while the particles themselves can be much larger due to their irregular shape. The interaction shell attributed to a given particle is a bit below the pore size, indicating formation of a shared particle-particle interaction layer of on the order of one pore diameter.

We use the area-weighted particle size distribution to estimate an overall intra-particle pore fraction for the EPI biomaterial. This gives an estimated intra-particle porosity fraction of 40% (confidence interval 27% to 53%), significantly greater than expected by mere random association of minority-colored particles (P=0.011 after Bonferroni correction for an overall of 3 tests for Fig. S9a, item 80 in Table S7).

In Fig S9b we analyzed the pore size associated with the intra- and interparticle space. We find that the pore size is significantly above the 50µm[12, 13] limit required for colonization and vascularization ($P=4.8*10^{-10}$ respectively $1.1*10^{-12}$, items 81 and 82 of Table S7), most likely explaining why the particles cannot easily be distinguished by the colonization patterns observed in histology (Figures 4k-4l in the main text). This being said, paired t-testing reveals a slightly lower pore size in the intra-particle pore space as compared to the intra-particle pore space (97+/-25 micrometers for the inter-particle pore size, vs. 80+/-27 micrometers in the intra-particle pore space, $P=2.4*10^{-6}$). This most like reflects constraints in arrangement of neighboring particles that lead to slightly larger space between the particles than within the particles, even though for cellular colonization, this seems relatively irrelevant.

5. Low-strain elastic storage modulus

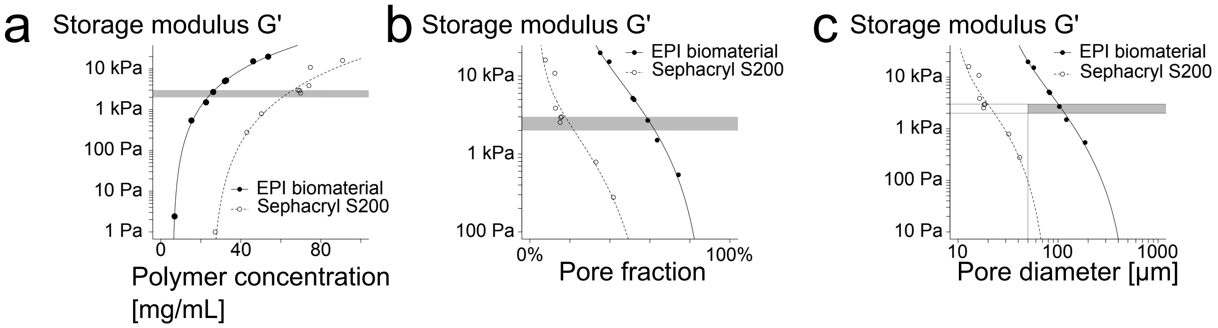

Figure S10: Storage modulus of the elastic porous injectable EPI biomaterial and Sephacryl S200 reference material. a) Low-strain G' (i.e. G'$_0$) as a function of polymer concentration b) Storage modulus as a function of pore fraction. b)The pore fraction was estimated by numerically solving eq. S5 (Chapter 4), with coefficients given in Table S1 (Chapter 4). For the theoretical, the theoretical G' values are additionally obtained from eq. S14 with coefficients given in Table S4. c) From the pore fraction, the pore diameter was estimated by eq. S7 with parameters from Table S2 (Chapter 4). The theoretical curves use eq. S14 with coefficients given in Table S4 for the G' values. The grey bar indicates the desired G' range of 2-3kPa for matching adipose tissue.[3, 15] In S5-1c, the restriction of a pore size remains larger than 50 micrometers to permit vascularization is also reported. With inverted axes, Figure S10c is also given in the main text (Figure 3i). Elastic storage moduli G' were measured by oscillatory shear rheology at a controlled stress of 1Pa applied at a frequency of 0.2Hz, at different polymer concentrations. If moduli below 100Pa were observed, the measurement was carried out at 0.1Pa stress instead. For all but very low G' values (<10Pa), the measurements in Figure S10 approach low-strain limits, as they were associated with shear deformations below 0.5%.

Figure S10 shows the dependency of the low-strain G' value G'$_0$ on the polymer concentration and pore space characteristics for the EPI biomaterial as well as the commercially available control material Sephacryl S200. Sephacryl S200 is a chromatography medium consisting of spherical, homogeneous microgels and thus serves as a theoretically well-understood reference.[16]

5.1. G'0 and polymer concentration

For both the EPI biomaterial and the Sephacryl S200 microgel suspension, we find a power-law to explain the concentration dependency seen in Fig. S11a:

$$G' = G'_0 \cdot \left(\frac{c-c_0}{c_0}\right)^m \qquad \text{eq. S14}$$

The theoretical lines in Figure S10a are best fit curves, with the parameters given in Table S4.

Material	G'_0	c0	*m*
EPI biomaterial	166.4Pa	5.95mg/mL	2.30
Sephacryl S200	738Pa	25.3mg/mL	2.94

Table S4. Power-law constants for the G' curves of the EPI biomaterial and the Sephacryl S200 reference material. The constants yield the curves shown in Figure S10a for the two

materials when used in eq. S14; they are least squares best fit values using a logarithmic scale as in Figure S10a for the G' values.

From the best-fit values given in Table S4, we conclude that two power laws are fundamentally similar since they have a similar exponent *m*. They are also close to the 3rd power law for the Young modulus of bulk cryogels observed previously.[4]

Of note, however, the Sephacryl S200 material has an over 4-fold higher critical onset concentration of c_0=25.3mg/mL as compared to 5.95mg/mL for the EPI biomaterial synthesized here to achieve finite G' values. This implies the EPI biomaterial achieves similar G' values with significantly lower polymer concentrations than the Sephacryl S200 reference material.

Material	G' range	Polymer concentration range
EPI biomaterial	2-3kPa	23-27mg/mL
Sephacryl S200		61-66mg/mL

Table S5. Polymer concentration required to achieve a low-strain G' in the range of 2-3kPa. For matching adipose tissue,[3, 15] we define a desired low-strain G' range of 2-3kPa. By solving eq. S14 for the polymer concentration, we estimate the required polymer concentration range in Table S5.

Figure S10a also shows the desired range of low-strain G' values of 2-3kPa for matching adipose tissue. From eq. S14, and the coefficients in Table S4, we calculate the associated range of polymer concentrations for two formulations. The results are given in Table S5. Given the steep dependency of the G' values on the polymer concentration, the concentration ranges required to meet the G' requirement are relatively narrow.

5.2. Elastic storage modulus and porosity

In Figure S10a, we have characterized the elastic storage modulus as a function of the polymer concentration. In chapter 4 of this document, we had analyzed total pore fraction and pore size, also in relation to polymer concentration. By combining the information, it is possible to directly relate the elastic storage modulus to the porosity characteristics.

5.2.1. Elastic storage modulus and pore fraction

First, we relate G'0 to the pore fraction (Figure S10b). From the known polymer concentration used for the rheological measurements of G' (Figure S10a), we can indeed estimate the available pore fraction in the samples by numerically solving eq. S5 (Chapter 4) for the pore fraction. To do so, we make use of the coefficients provided in Table S1 (Chapter 4).

Figure S10b shows the results: the G' values from the dataset of Figure S10a are re-plotted as a function of the estimated pore fraction associated with each polymer concentration. For both materials, higher pore fractions correspond to lower G' values, as expected from the fact that higher pore fractions mean higher hydration levels.

In addition, at similar storage moduli, the EPI biomaterial displays higher porosity than the Sephacryl reference material. For instance, for a low strain G' value in the 2-3kPa range, the EPI biomaterial displays a porosity of 58%-63%, whereas the Sephacryl reference material only displays a porosity of 18-22% (calculation from the theoretical curves). We attribute this difference primarily to the presence of internal porosity within the particles of the EPI biomaterial, compared to only interstitial porosity in the Sephacryl reference material.

5.2.2. Elastic storage modulus and pore size

Similarly to the approach taken in Figure S10b, we also plot the G' data as a function of the pore size rather than pore fraction (Figure S10c). For this, we use eq. S7 (Chapter 4) along with the coefficients in Table S2 (Chapter 4) to convert the pore fraction values to estimated pore size values. The G' values as a function of the estimated associated pore diameters are shown in Figure S10c, which with inverted axes is reported as Figure 3i in the main text.

Figure S10c shows the relation between pore size and G' values. Larger pore size can be achieved by lowering the polymer concentration; this correspondingly lowers the material's stiffness so that the G' vs. pore size curves have a negative slope. While a very wide range of stiffness G' can be achieved simply by adjusting the polymer content, it is more difficult to meet both the 2-3kPa[3, 15] stiffness requirement and the requirement of 50 micrometers or more to permit vascularization.[12] Whether or not this is possible can be determined by checking whether the G' vs. pore diameter curve crosses the shaded target area in Figure S10c. Here, one sees that it is the case for the EPI meta-biomaterial, but not the Sephacryl S200 reference material. We therefore conclude that with the EPI meta-biomaterial, it is possible to meet both the stiffness and pore size requirements, whereas with the Sephacryl S200, either one, but not both simultaneously can be met.

6. Additional rheological data

6.1. Viscous loss modulus G’’

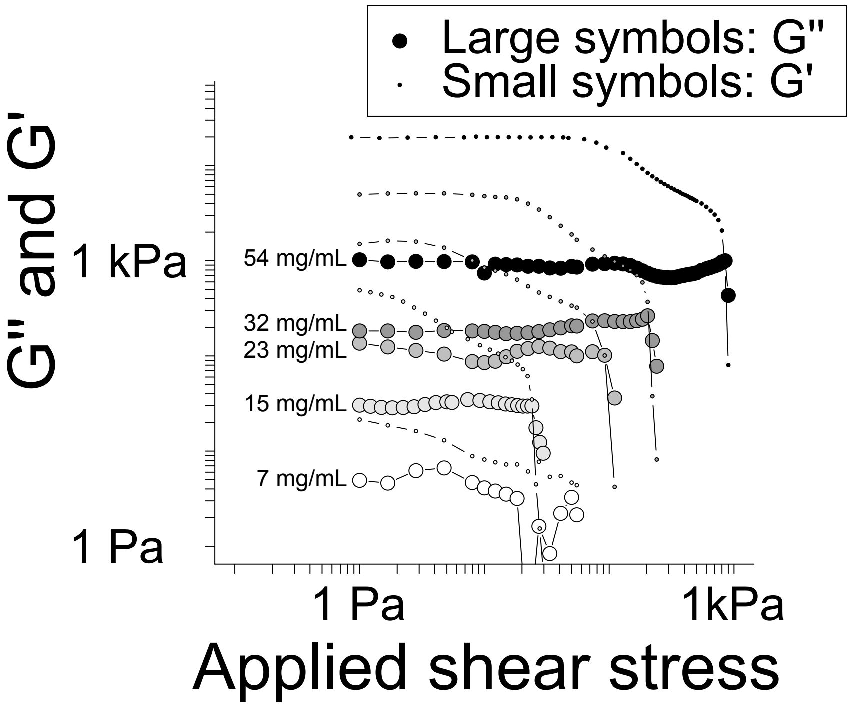

Figure S11: Viscous loss modulus G’’ associated with the elastic storage modulus G’ values presented in the main text, Figure 3f. Measurements at 0.2Hz.

Figure S11 presents the viscous loss modulus G’’ associated with the G’ curves shown in the main text (Figure 3f). For reference, the G’ curves given in Figure 3f in the main text are also provided, using small symbols. Figure S11 indicates that for most of the shear stress range examined, the EPI biomaterial behaves as an elastic solid. Indeed, on the low-strain plateau (not reached for the 7mg/mL concentration, barely reached at 1Pa shear stress for the 15mg/mL formulation, and reaching up to 100 Pa shear stress for the 54mg/mL formulation), the G’’ value is only a few percent of the G’; even after the softening transition, on the second plateau of the G’ curves, the G’’ remains substantially below the G’. Only at the yield transition we find a dramatic drop of G’ values giving rise to crossing between the G’ and G’’ curves.

6.2. Yield strain

6.2.1. Yield strain from G’ and G’’ curve intersection

Yielding is usually analyzed from flow-curves.[5] Flow curves report the steady-state shear stress level at a given deformation rate,[5] and so for fluids with a defined yield point, the yield stress should be reached directly at even the lowest non-zero deformation rates. This steady state approach precludes the possibility to estimate the associated yield strain (see Figure 6, p. 21 in [5]). In practice, deformation up to the yield point will take some time at low deformation rates, and so in principle, one could analyze the deformation during the rise of the shear stress to obtain an estimate of the yield strain. Since rheometers are typically built to apply large deformation to viscous fluids, it is however difficult to apply deformation rates low enough to accurately infer the yield strains in this way. We use the intersection point of

the G' and G'' curves when plotted against the oscillatory shear strain should circumvent this technical problem.

The CodeOcean capsule[2] associated with this manuscript provides technical details on the rheology setup, as well validation data on the G'/G'' intersection method (https://doi.org/10.24433/CO.6934377.v1, folder "Figures_CodeOcean_only" in the Results section, Figures C4-C7, description in CodeOceanOnlyResults.pdf).

6.2.2. Ensuring data quality

It is necessary to take some precautions to ensure sufficient data quality for accurate yield strain estimation from G' and G'' curves. Trivially, we cannot estimate a yield strain from curves where the G' and G'' do not cross, or where they cross multiple times due to the presence of excessive noise at very low polymer concentrations.

In addition, there is a particular issue with the preferred stress- rather than strain-controlled operation of the Rheostress RS60 rheometer. Stress indeed continues to be applied even when the samples yields. Crossing the yield point therefore results in sudden, possibly very rapid chuck rotation. This poses a safety hazard due to possible sample dispersal, which will be addressed by suitable shielding. But also, this results in potentially very large deformation values beyond the yield point.

This problem is much more pronounced with the brittle Sephacryl S200 reference material than with the more ductile EPI or HA ctl samples. It leaves an identifiable signature in the data evaluation: we estimate the yield strain by linear interpolation of the G'/G'' crossing point between the last solid-type (G'>G'') and the first liquid type (G'<G'') measurement (interpolation in log scale). If the yield strain estimated in this way is much larger than the strain at the last solid-type measurement, brittle failure has probably taken place and the yield strain is exaggerated.

From numerical simulation, we expect a factor of 3-4x of difference between the yield strain of irregular vs. spherical particles (Figure 3b-3e in the main text). As the incertitude arising from rapid spinning after brittle failure should not be larger than this difference, we should treat samples with more than a 3x difference between the last precise strain estimation in the solid-state regime and the final estimation of the yield strain with caution.

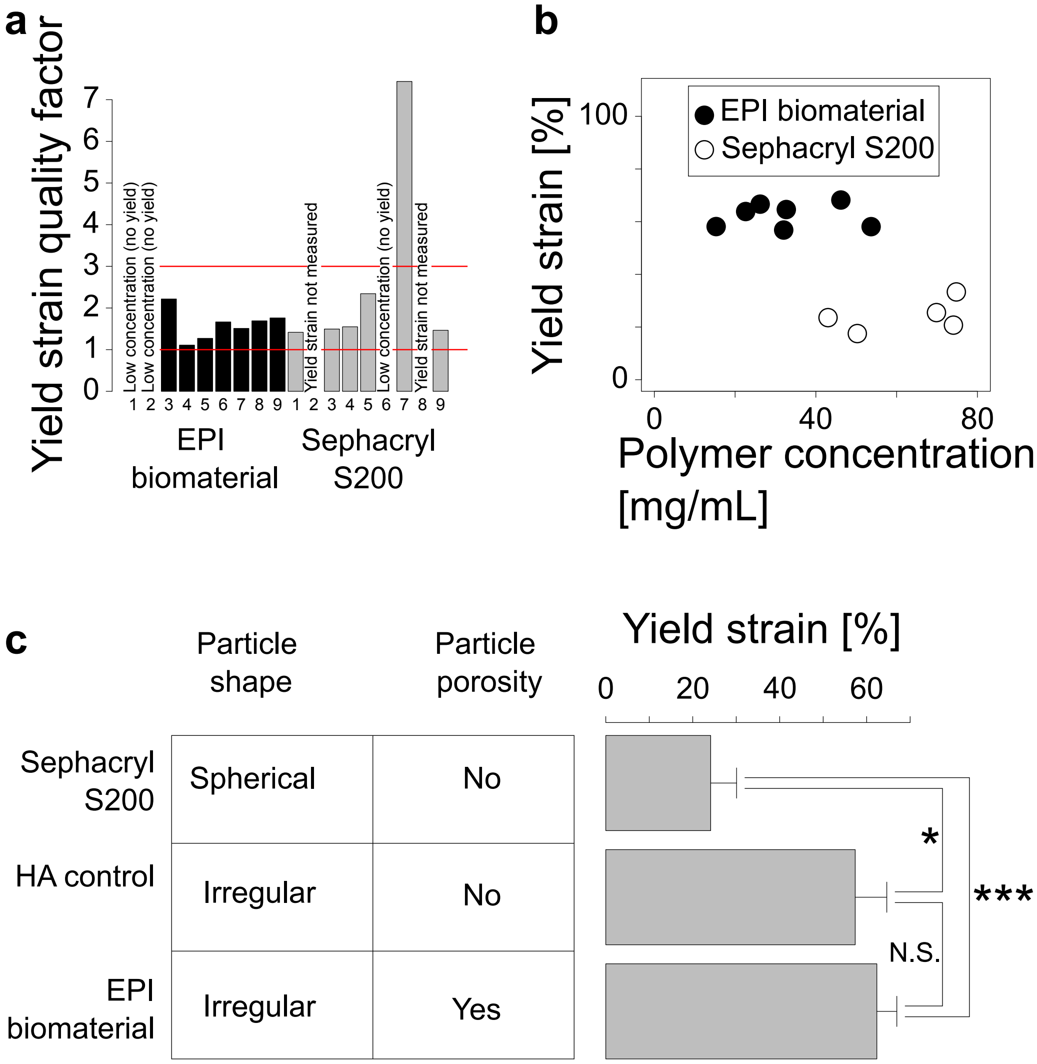

Figure S12. Yield strain of EPI biomaterial and Sephacryl S200 samples at various polymer concentrations. a) Preliminary data quality analysis. In some cases, yield strain could not be determined for technical reasons, this is indicated textually. As a measure of the quality of yield strain estimation, we used the ratio of estimated yield strain to highest strain measured in the solid-state regime. An upper bound of 3 was imposed on this ratio, as higher values signify an extreme difference between the support points for the linear regression and the evaluation point. b) Relation between yield strain and polymer concentration for the retained stress sweeps. c) Comparison with commercial hyaluronic acid HA control (same method of G' / G'' crossing, but in plate-plate geometry). Errorbars are one standard deviation. Statistical details in Table S7, item 27.

6.2.3. Results

Data quality and selection

Figure S12a and S13b show the analysis of the yield strain for the 9 stress sweeps acquired for the EPI biomaterial, respectively the Sephacryl S200 reference material in the custom cup geometry. Figure S12a gives an overview over the dataset used. Out of originally 9 sweeps at different concentrations for each material, for three samples (two EPI biomaterial samples,

one Sephacryl S200 sample), the polymer concentration was too low to properly evaluate yielding. For the Sephacryl S200 material, there were also two stress sweeps where the stress applied was too low to reach yielding, and no yield strain could be determined for these samples.

Within the stress sweeps amenable to proper analysis, we evaluated the crossing point of the G' and G'' curves by linear interpolation in log-log plots of G' and G'' against shear strain (analogously to Figure S6-1 with the x-axis being shear strain rather than shear stress). As a measure of the quality of this evaluation method, we determined the ratio between the yield strain estimated by this linear interpolation method, and the highest observed strain in the solid-like regime with G'>G''.

Figure S12b shows this quality factor for the sweeps that we could evaluate. As discussed above, we set an upper limit of a factor of 3x difference between the interpolated yield strain and the highest measured actual strain before liquefaction. Indeed, if there is more than a factor of 3 of difference between the two strains, the yield strain estimation by linear interpolation should be considered very unreliable as there is no close measured strain supporting it. For the EPI biomaterial, we find a mean ratio between estimated yield strain and highest measured pre-yielding strain of 1.6, fully acceptable in light of the anticipated differences between spherical and irregular microgel suspensions. For the Sephacryl S200, the ratio of yield strain to highest measure pre-yielding strain is higher (mean value of 2.6), primarily to the presence of one extremely high value (7.4). As linear interpolation with support points distant by nearly an order of magnitude is unacceptable, we excluded this particular measurement from our analysis. Physically, this corresponds to a measurement where a cleft was created in the Sephacryl material with onset of rapid spinning of the chuck, and the very large apparent strain value estimated by free rotation of the chuck can hardly be considered a reasonable estimate of the yield strain.

The analysis in Figure S12a illustrates the difficulty of measuring the yield strain of a brittle material such as Sephacryl S200 with a predetermined stress ramp: at the yield point, unpredictable displacement or fragmentation of the material sometimes, but not always, allows sudden very large chuck rotations, precluding proper yield strain determination. This problem was specific to the Sephacryl S200 reference material.

We finally dispose of 5 reliable measurements of yield stress at polymer concentrations of interest for the Sephacryl S200, vs. 7 measurements for the EPI biomaterial. For these samples, Figure S12b shows the yield strain as a function of polymer concentration.

6.2.4. Influence of material type and polymer concentration

Statistical analysis by linear regression using both the polymer concentration and the sample type as explanatory variables indicates a significantly higher yield strain for the EPI biomaterial as compared to the Sephacryl S200 reference material (Figure S12c, $P=1.4*10^{-5}$, Item 27 in Table S7), as suggested by the simulations for irregular vs. spherical particle shape. No influence of polymer concentration on the yield strain can be detected (Figure S12b, P=0.41, Item 73 in Table S7). The limited influence of packing density on yield strain (as opposed to the strong influence on yield stress, see Figure S6-2), is known in the literature for spherical microgel suspensions and has been attributed to geometrical slippage relatively independent from the compressive state of the suspension[17]. Our data confirms a similar

relative independence of yield strain on polymer concentration in suspensions of irregular particles, albeit at higher absolute values.

6.2.5. Estimation of yield strain: Sephacryl S200, Hyaluronic acid HA control, and EPI biomaterial

Since no influence of polymer concentration can be detected, Figure S12b can directly be used to estimate the intrinsic yield strain of the two materials: 62 +/- 5% for the EPI biomaterial, vs. 24 +/- 6% for the Sephacryl S200 reference.

For Figure S12c, we additionally performed a similar yield strain analysis on the three stress sweeps of Hyaluronic acid control (HA ctl) samples used for Figure 3g in the main text (Juvéderm Voluma®). The HA ctl material is a smoothly behaving viscoelastic gel, and we encountered no particular problems at yielding, all three estimations satisfied the quality criteria set forth above. The yield strain was evaluated to 57 +/- 7%.

Figure S12c then compares the yielding behavior of the Sephacryl S200 spherical microgel suspension, the HA ctl injectable, and the EPI scaffold. The Sephacryl S200 material shows a significantly lower yield strain than the two other materials (P=0.011 for Sephacryl S200 vs. HA ctl, $P=1.4*10^{-5}$ for Sephacryl S200 vs. EPI), whereas we do not detect a significant difference between the HA ctl and the EPI biomaterial samples (P=1.0, details of the statistical analysis in item 27, Table S7).

6.3. Comparison between EPI biomaterials and control materials

Figure 5b and 5c in the main text report an overview over the rheological characteristics of a set of materials, both with regular and irregular, and porous and non-porous particles.

Figure S13 shows the detailed rheological measurements underlying Figure 5b and Figure 5c in the main test. By comparison, Figure S13a and Figure as S13b indicate that particle porosity greatly amplifies strain softening as the G’ values show a much more important decrease before the yield point. Also, as expected, there is nearly ubiquituously a clear plateau separating the strain softening transition from the actual yielding transition (at very low polymer concentrations, corresponding to exceedingly soft suspensions difficult to measure, the plateau tends to smear out somewhat). Strain softening is quantitatively much less important in non-porous particle suspension (Figure S13b), as anticipated.

There is a significant shift to the top left of the yield region for the spherical particles compared to the irregular ones (for both Figure S13a and S13b, items 89 and 90 in Table S7), reflecting systematic differences in yielding behaviour between spherical and irregular particles, again as anticipated. In Figure S13b, for some of the non-porous irregular particles (to be precise, the ones synthesized by ourselves by extrusion analogously to the EPI, but not the commercial HA control) some strain softening also occurs, although the effect is much less prominent than with porous particles and it would be difficult to ascertain the existence of a distinct softening transition with these materials. Yet, some minor strain softening even in the absence of porosity is in agreement with our simulations. The reason why strain softening seems to completely absent in classical non-porous spherical microgel suspensions and also in the dermal filler HA control are not entirely clear; they may in part be rooted in the early yielding (spherical microgel suspensions), but also tight assembly of nearly polygonal

particles (Figure 3b in the main text) with difficulties in translocating pore fluid in the case of the HA control material, removing the necessary material flexibility for strain softening despite a high yield strain.

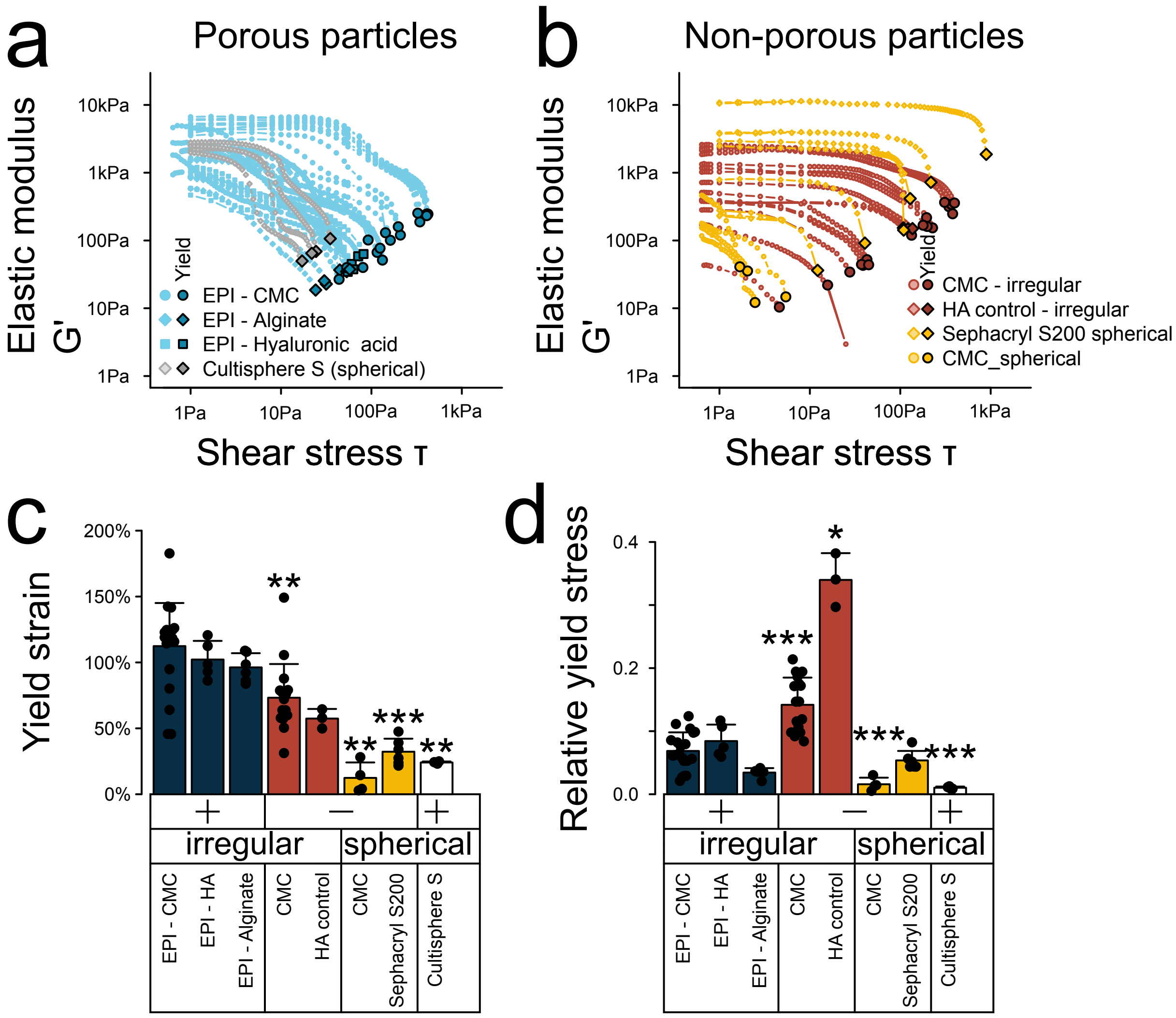

Figure S13. Detailed rheological characteristics of the EPI biomaterial (EPI-CMC), the various analogs (EPI – Alginate and EPI-Hyaluronic acid HA), as well as the various control materials (synthesized and commercial). A summary of this data is reported in Fig. 5b and 5c in the main text. a) Oscillatory rheological sweeps of a collection of porous materials (EPI realized in CMC, alginate and HA) and Cultisphere S (spherical, but porous microcarrier particles). The difference in location of the yield region (darker symbols) between the EPI materials and Cultisphere S is significant (item 89 in Table S7). b) Identical analysis as in Figure S13a but for non-porous particles (crosslinked CMC, both spherical and irregular, as well as commercial HA control and Sephacryl S200). Location shift between spherical and irregular particles significant (item 90 in Table S7). c) Yield strain for the particle suspensions. d) Relative yield stress for the different particle suspension. This is the yield stress as measured by oscillatory rheology relative to the plateau stiffness value G'0 measured on the same curve at very low deformation.

Figure S13c shows the yield strains observed on average for the different materials. These data are tabulated over a range of different polymer concentrations. Indeed, as shown in Figure S12, the polymer concentration has a relatively minor impact on yield strain. In this

dataset, the various EPI biomaterials have the highest yield strain, followed by irregular non-porous particles, whereas spherical particle suspensions have low yield strains regardless of porosity.

Figure S13d tabulates the relative yield stress (defined as yield stress / stiffness G0' at low deformation). Figure S13d indicates that the EPI meta-biomaterial design indeed generally provides favorability injectability (low relative yield stress) with high yield strain (Figure S13c). Irregular particles without porosity and thus compressibility may also achieve high yield strain, but at the cost of difficulties in injection due to high relative yield stress. Spherical suspensions, regardless of porosity, have favorable low relative yield stress but lack the desired high yield strain.

7. Additional injectability data

7.1. EPI injectability force through a larger cannula

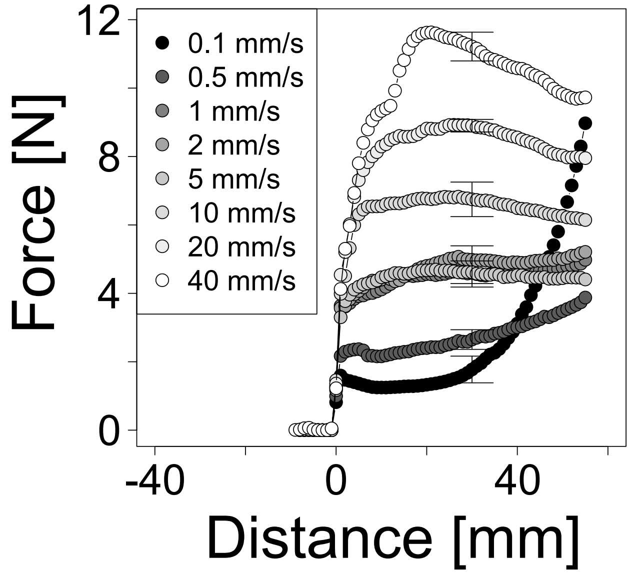

Figure S14: Injectability assessment of the EPI biomaterial. Testing was done by using a large-volume delivery cannula (2mm outer diameter, 10cm length, Thiebaud Biomedical devices, ref. F9020100)

Figure S14 shows our set of force-distance curves that we acquired with the EPI biomaterial to assess injectability through a large-volume delivery cannula for different injection rates[18] (2mm outer diameter, 10cm length, Thiebaud Biomedical devices, ref. F9020100).

7.2. Maximum force and controls

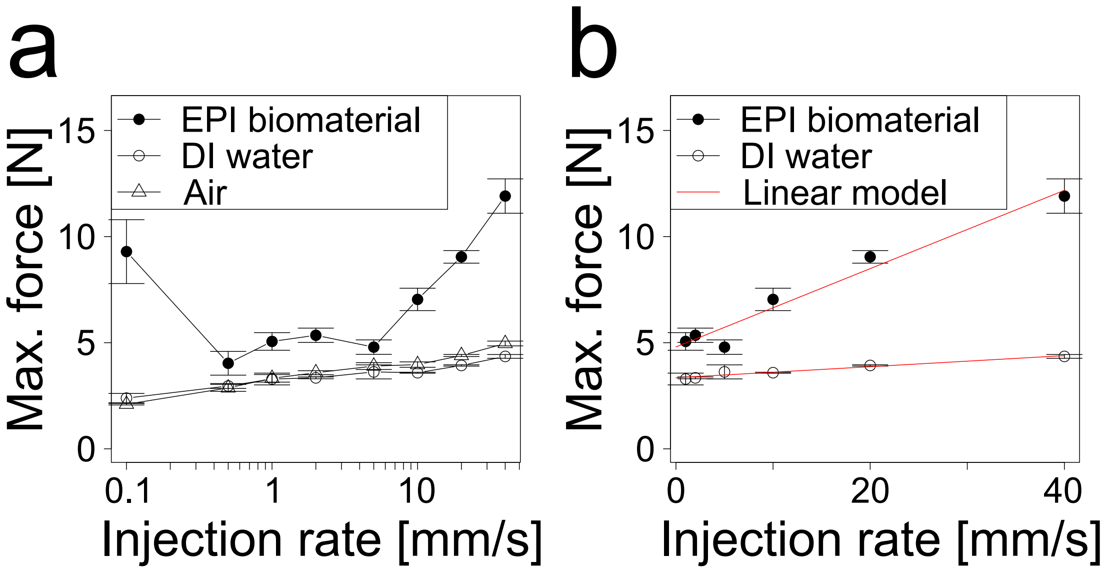

Figure S15: Maximum force (large-volume delivery cannula as in Figure S14). a) Maximum force required during injection of EPI biomaterial, DI water and air from 1mL syringes into a Falcon tube at various injection rates. b) EPI scaffold and DI water, at higher speeds (1mm/s and above) and analysis by two factor linear regression with interaction. Dataset: same as in Figure S14, additionally with DI water and air control.

We also carried out injection force measurements using syringes filled with deionized water and air. To better understand the origin of the measured forces, we plotted the maximum injection force required against the injection rate. Figure S15a shows the results. As expected, the ejection of the EPI biomaterial requires more force than merely moving the piston of an air- or water-filled syringe. Whether the syringe is air- or water-filled has little influence on the force required to move the plunger, indicating that friction between the plunger and the syringe barrel rather than movement of the control media dictates the necessary force. The EPI biomaterial significantly contributes to ejection force. Interestingly, the force required to eject the biomaterial is minimal for intermediate, rather than very low injection rates. At very low rates, rather than being ejected homogeneously, pore fluid is squeezed out first, resulting in a very dense material that finally opposes very high forces to ejection. Techniques to address this issue include addition of a viscosing agent to the pore fluid and adjustment of the particle geometry. With such approaches, injectability through 27G needles even at low speeds can be achieved; the details will be published elsewhere.

Comparison with the force required for ejection of DI water indicates that for moderate ejection rates (0.5mm/s to 10mm/s), the EPI biomaterial contributes less force than the plunger movement and thus should be considered perfectly injectable. At the highest ejection rates (20 and 40mm/s), more force is again required, presumably to overcome the viscous contribution of the EPI biomaterial.

Figure S15b explicitly analyzes the higher injection rates (1mm/s and more) where the EPI material is expected to move without loss of water. To better assess the force generated specifically by the EPI biomaterial, we use joint linear regression of this data and the corresponding maximum force data from the deionized water control. We include an interaction term between the injection rate and the sample type (EPI vs. DI) to allow for a different slope in the two groups (red lines in Figure S15b). Table S6 shows the results of the analysis of variance ANOVA on this model. All terms are significant: there is a non-zero global offset (stick and slip of the syringe Piston), an additional additive contribution of the EPI material vs. DI (we interpret this as being the result of a finite elastic yield stress required to initiate flow in the EPI biomaterial), a positive contribution of injection rate (viscosity effects) and also a significant interaction term, which we interpret to reflect the higher viscosity of the EPI biomaterial compared to the water control. Details of the statistical evaluation are given in Table S7, item 33.

Term	P-value (Bonferroni for 3 tests)	Fraction of variance	Interpretation
Injection rate	$9.8*10^{-6}$	31.6%	General viscosity effect, faster rate leads to higher force
Sample type	$1.9*10^{-6}$	48.6%	Higher force required to inject EPI scaffold as compared to DI water
Interaction	$8.3*10^{-5}$	17.8%	Force increases faster with higher injection rate for EPI than for DI water
Residuals	N.A.	2%	Unexplained variance

Table S6. ANOVA for linear model for maximum injection force. Data restricted to higher injection rates (1mm/s and more) to avoid drying of EPI scaffold. Comparison between DI water and EPI scaffold over the different injection rates. Details of the statistical evaluation in Table S7, item 33.

Of note, the drying effect visible at very low injection rates in Figure S15a becomes more prominent with thinner needles; with very thin needles (20G or thinner) and very low speeds (0.1mm/s), it is possible to essentially separate pore fluid from solid material. This is highly non-desirable. However, by control of particle size and pore fluid viscosity, it is possible to prevent this defavorable effect and enable successful injection up to 27G at reasonable rates. A manuscript presenting this in deeper detail is in preparation.

8. Complete dataset on self-healing assessed under oscillatory shear rheological testing

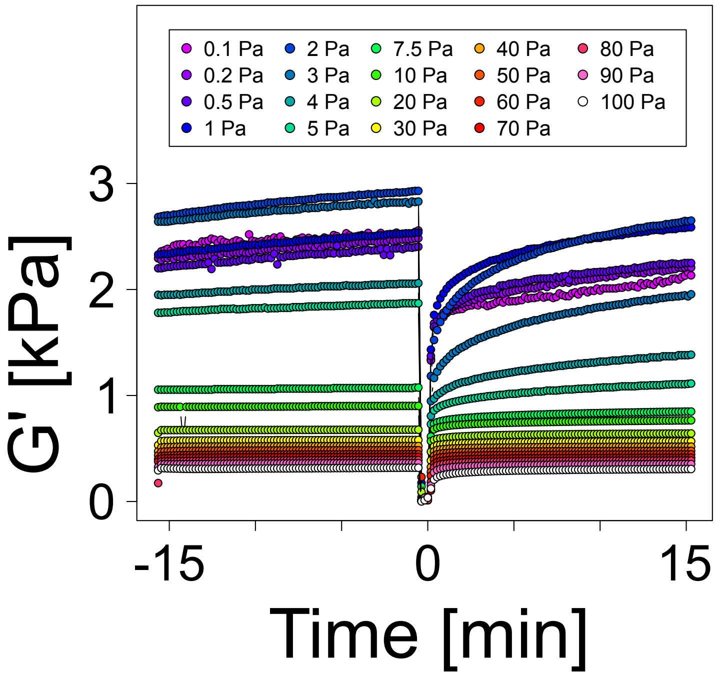

Figure S16: Average G' recovery for the different conditions. The curves shown in Figure S7-1 were aligned in time (see text), followed by averaging for curves corresponding to the same predefined stress level (see legend in both Figure S16 and here for the applied stress values).

Figure S16 shows the complete self-healing dataset after alignment in time and averaging for the different experimental runs with the same predefined shear stress level. Selected aspects are shown in the main text (Figure 3l and Figure 3m). It can be seen that generally, higher stress levels are recovered with lower applied shear stress; in detail, recovery is however fastest at intermediate rather than the lowest shear stress levels.

For increased readability, Figure 3l in the main text shows a subset of the data presented in Figure S16 (0.1Pa, 1Pa, 10Pa and 50Pa of continuous oscillatory shear stress). This minimal subset illustrates the fundamental information contained in Figure S16: general decrease of recoverable G' with increasing stress, but optimal recovery kinetics at intermediate rather than lowest shear stress values.

Figure 3m in the main text is generated from the data presented in Figure S16 by plotting the G' values achieved at the end of the strong shear period ("0s" in Figure 3m), as well as after 15s, 30s, and 15 minutes of recovery. The baseline value in Figure 3m is taken as the last value under constant applied shear just prior to application of the strong shear period.

On CodeOcean[2], the dataset shown in Figure S16 before average per oscillatory monitoring shear stress as well as deeper analysis of the dataset can be found by interested readers (https://doi.org/10.24433/CO.6934377.v1, folder "Figures_CodeOcean_only" in the Results section, Figures C8-C11, description in CodeOceanOnlyResults.pdf).

9. Rheological comparison of the EPI biomaterial to adipose tissue

In the main text in Figure 3g, we compare the EPI biomaterial and the hyaluronic acid filler control material to literature data on adipose tissue (subcutaneous, human, from [15]). Here, in Figure S17, we provide further comparison to additional adipose tissue sources.

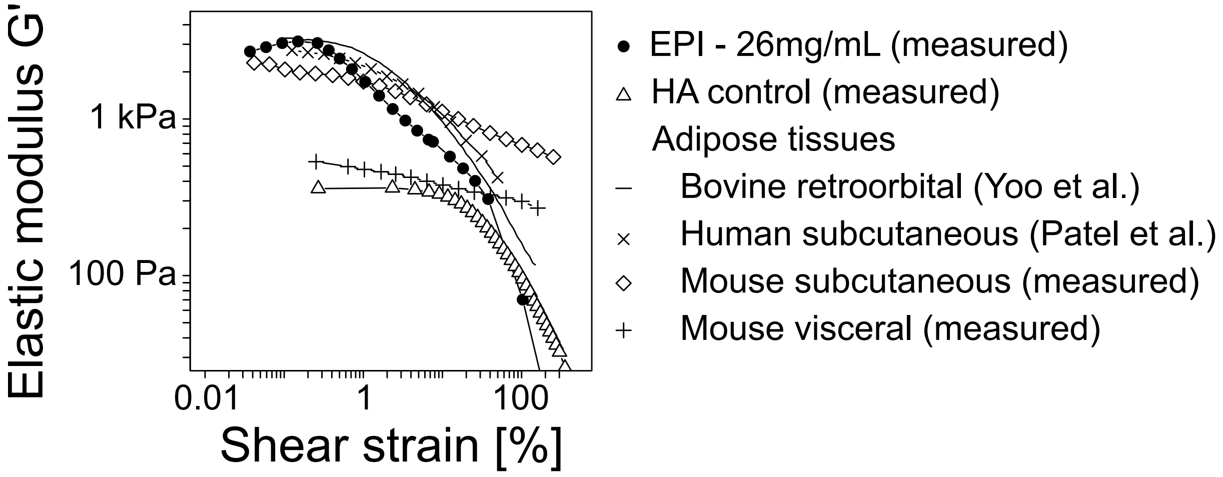

Figure S17: Rheological comparison to adipose tissues. The EPI biomaterial and the hyaluronic acid filler control material are compared to various adipose tissues by means of rheological oscillatory stress sweeps. The elastic storage moduli curves for the EPI and HA control as well as the literature data from human subcutaneous adipose tissue (Patel et al.[15]) are the same as in Figure 3g, the others are additional. For the human adipose tissue, the data provided by Patel et al.[15] represents the total modulus $G = \sqrt{G'^2 + G''^2}$ and therefore overestimates G', although for the strains applied, we would expect $G'' < G'$. The bovine retroorbital fat tissue is from Yoo et al.[3]

Human subcutaneous adipose tissue is of primary interest, and has been characterized by Patel et al.[15] As shown in Figure S17, the human subcutaneous adipose tissue has rheological properties very similar to bovine retroorbital adipose tissue,[3] the latter being possibly easier to source for non-clinical research groups.

This being said, there are some methodological discrepancies that need to be discussed in the comparison of the various datasets. First, the dataset by Patel et al.[15] on human subcutaneous fat tissue provides the total modulus $G = \sqrt{G'^2 + G''^2}$ rather than the elastic storage modulus G'. However, both literature data[3] and our own measurements on subcutaneous and visceral adipose tissue from mice indicate that for the range of applied strains, the viscous loss modulus G'' is substantially smaller (a factor of at least 3) than the elastic storage modulus G', and so $G \approx G'$ should hold approximately.

A further difference between the various datasets is the exact frequency applied during oscillatory rheology. We use 0.2Hz, while the literature datasets apply frequencies in the range of 0.05Hz to 5Hz.[3, 15] For primarily elastic materials, the frequency used for oscillatory shear rheology has only a minor influence on the G' values, both on theoretical[5] and empirical grounds (literature data[3, 15], preliminary frequency sweeps not shown). Together with other variations in setup details, we anticipate that the comparison between literature data and the empirical measurements of the EPI biomaterial and HA control, drawn in Figure

S17, has semi-quantitative value: the tendencies are fully applicable, but experimental details may induce quantitative incertitude on the order of about a factor of 2 in linear scale.

We therefore also proceeded with empirical characterization of adipose tissue available to us from mice in rheological stress sweeps (also shown in Figure S17). These measurements confirm the low-strain limit of about 2kPa for subcutaneous tissue, and indicate lower values (about 0.5kPa) for the visibly softer visceral adipose tissue.

We obtain somewhat higher G' values at high strains than in the literature datasets. This is most likely related to the difficulty of appropriately separating all connective tissue from the adipose tissue for the rather small mouse tissue samples. Indeed, at high deformation, even small amounts of collagen retain the tissue and avoid rupture, leading to larger G' values and retardation of the liquefaction point.

Taken together, the results provided indicate that the EPI biomaterial has rheological properties approaching the ones of a possibly relevant clinical target tissue, namely human subcutaneous adipose tissue.[15] Judging by the low-strain G' limit, the origin (visceral or subcutaneous) of the adipose tissue is more important than the species origin (human, bovine or murine) regarding the mechanical properties. There are however methodological and experimental limitations that imply that these comparisons should be taken as indicative, rather than fully quantitative.

10. Quantitative simulation and the frictional effect of collagen

When coating EPI meta-biomaterial with collagen I to enable adhesion of cells for cell transplantation applications, we noticed that at the highest collagen fractions (10% collagen I with respect to dry mass), the EPI material becomes extremely sticky and difficult to inject. Indeed By rheological stress sweeps, we quantified the changes occurring in the 10% collagen-modified EPI material. The results, shown in Figure S18 (and, among others, Figure 5e-5g in the main text), confirm the macroscopic impression: The 10% collagen coating completely abolishes the elastic softening transition, and shifts the relative yield stress towards values that are no more compatible with combining high stiffness and facile injectability.

Such change in macroscopic behavior could not be observed at lower amounts of collagen I, where cell adhesion was still possible (main text). This demonstrated on the one hand that cell adhesion is compatible with the desired meta-material behavior for high injectability, stiffness and shaping (see main text). On the other hand, this shows nevertheless that there are limits to chemical modification compatible with the desired meta-material behavior and provided an occasion to investigate the model parameter changes associated with this apparent loss of elastic strain softening.

In order to understand how such limitations arise, we fine-tuned our simulation such as to be able to not only qualitatively, but quantitatively explain the EPI biomaterial rheological behavior.

To achieve a quantitative match between simulation and rheological behavior of actual EPI biomaterial, we note the known extreme compressibility of cryogels[4]. As outlined in the CodeOcean capsule[2], it is possible to incorporate such extreme compressibility also into the simulation to achieve quantitative matching with experimental data by the use of a plateau compression law[19] instead of simple linear particle compressibility (https://doi.org/10.24433/CO.6934377.v1, folder "Figures_CodeOcean_only" in the Results section, Figures C12, C13, description in CodeOceanOnlyResults.pdf).

In Figure S18, we first achieved a quantitative match between the rheological master curve of the normalized G' curves of a given set of EPI biomaterial samples at different polymer concentrations. We then varied different parameters of the model to match the rheological master curve also for a 10%-collagen coated variant, which has lost the strain softening phenomenon. We find that the single most relevant parameter to explain the observed shift in the rheological behaviour is the friction coefficient. To put it simply, if the material making up the porous particles is too sticky and provides too much friction, the flexibility necessary for a softening transition prior to yielding is lost.

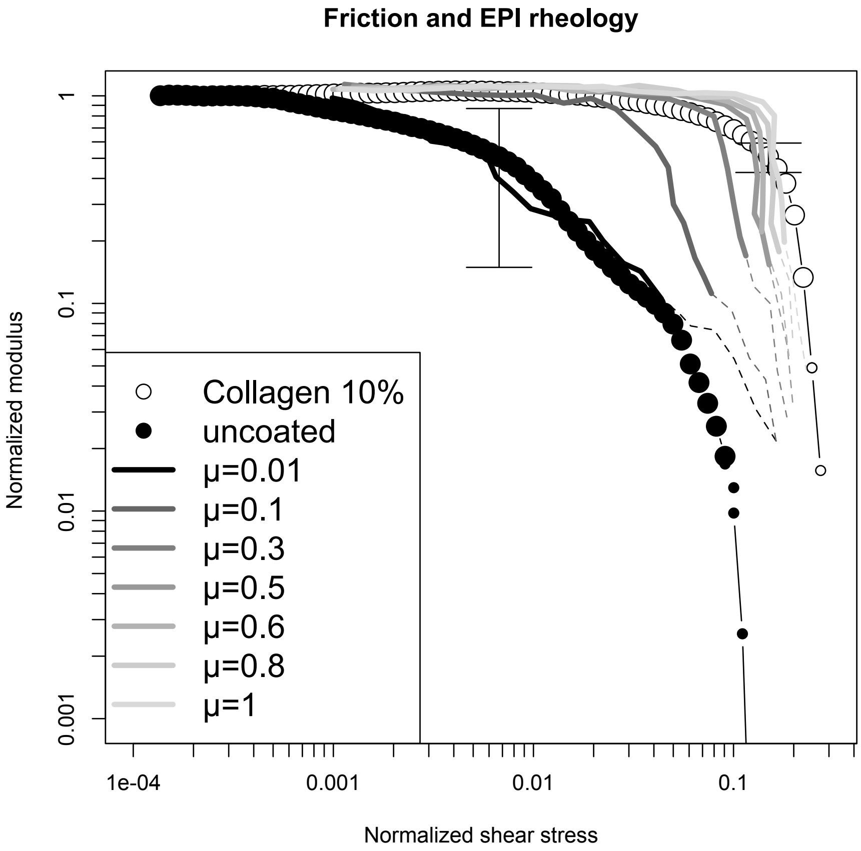

Figure S18. Rheological master curves, measured on uncoated EPI meta-biomaterial, and also on EPI meta-biomaterial functionalized with 10% dry mass content collagen. Comparison with numerical simulation, using a plateau force contact law as in Figure S10-2, but with various settings for the friction coefficient μ. Thick main lines respectively large symbols indicate the regions with solid-like behavior (G'>=G''), while thin lines respectively small symbols indicate liquid-like behavior (G''>G'). The μ=0.01 and μ=0.8 simulations are also reported in the main text, Figure 5e.

The observations made here with strong collagen modification shed a particular light on the role of the friction coefficient in elastic strain softening: To obtain an elastic strain softening transition, the friction coefficient should be neither too high nor too low. If it is too low, for practical purposes, one faces a non-frictional material without strain softening transition.[19] If it is too high, the strain softening transition is masked since it merges with the yielding transition. This constraint is probably even stronger in spherical microgel suspensions, since they yield at lower stress and thus make the window even narrower. It is tempting to speculate that this is one of the main reasons why strong elastic strain softening has proven difficult to observe in microgel suspensions that are not specifically engineered for that purpose.

11. Long-term outcome with a more dilute formulation

A final question that we had was what could possibly happen beyond scaffold degradation. This answer is difficult to obtain from injections of the EPI biomaterial as formulated for shaping. Indeed, in Fig. 4l we reach only about the maximum degradation rate of the scaffold (see also Fig. 4m), with maximum loading of the cells with scaffold fragments, but not yet clearance of these products. While some fibrovascular meshwork subsists even in these conditions, it is not clear how the tissue would evolve after the degradation peak.

Therefore, in order to obtain a possible answer to the evolution beyond scaffold degradation, we diluted the reaction mix by adding 1 part of deionized water to 2 parts of reaction mix before cryogelation. We processed the resulting, substantially weaker, scaffold similarly to the EPI biomaterial and injected this into mice. Due to the weaker mechanical properties, this type of scaffold does not provide for permanent shaping capacity, but otherwise provides similar cellular ingrowth response.

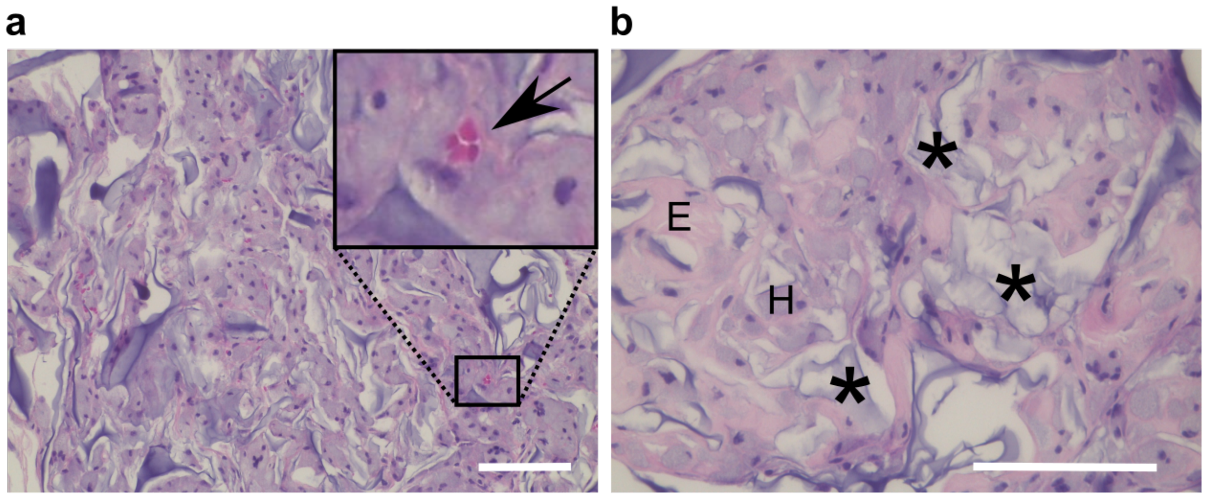

Figure S19. Long-term follow-up of EPI biomaterial at decreased premix concentration (2:1 part deionized water) to accelerate degradation. 1 year follow-up. a) Histology of the EPI at decreased synthesis concentration to accelerate degradation, at 1 year. Example of a small

vessel shown in magnified inset. Scale bar=100µm. Not shaped, 200µL bolus. b) Same condition as a), higher magnification image. Degrading scaffold denoted by stars, E=example of eosinophilic region, H=example of region stained by hematoxylin. Images adjusted regarding white balance and luminosity.

Fig. S19 shows the results of a one-year study with the EPI biomaterial at decreased synthesis concentration. Globally similar to the results shown in Fig. 4l in the main text, one can note that the load of scaffold material is smaller, but most importantly also that relatively little intact scaffold fibers persist, indicated more advanced degradation as desired. In Fig. S19b one can notice that there are relatively large areas of eosinophilic, connective tissue areas recovering from the degradation load. We interpret this as an indication that following scaffold degradation, the heavily material-loaded cells can recover to reconstitute a connective tissue. For how long and to which extent this tissue is maintained is however beyond the reach of a mouse model.

12. Statistical reporting

Table S7 below reports items pertaining to descriptive or hypothesis testing statistics relevant to the figures of the main text and the supplementary figures provided here. Table S7 is a summary table, the complete table including detailed explanation, normality testing, confidence intervals, detailed mitigation strategy of non-normality, indication of central tendency and variability measures, as well as additional tests regarding the figures provided only on CodeOcean is available on CodeOcean[2] (at /code/Documentation/Statistical Reporting.xlsx).

In statistical hypothesis testing, we applied Bonferroni[20] multiple testing correction per subfigure or number of regressors (relevant number of tests indicated in Table S7). Regarding mitigation of non-normality, we generally attempted parametric testing in all cases, and then analyzed the resulting residuals for non-normality using the Shapiro-Wilks[21] respectively Royston[22] test in the multidimensional case. If the P-value of these normality tests (provided on CodeOcean[2]) was 0.05 or less, we instead report here the corresponding non-parametric test. In the case of linear regression, the issue of non-normality of the residuals could be addressed by using a general linear model permitting a better adapted error structure.[23] Among the CodeOcean only figures, we also had to resort to non-parametric Siegel regression[24] (see CodeOcean capsule[2], document /code/Documentation/CodeOceanOnlyResults/CodeOceanOnlyResults.pdf, figure C5 and additional item 69 in /code/Documentation/Statistical Reporting.xlsx). Of note, resorting to non-parametric tests in the case of a significant normality test had little influence on our statistical conclusions. There was indeed no case where rejection of a null hypothesis under the parametric test was reversed by the corresponding non-parametric test following a significant non-normality pre-test in our dataset.

In Table S7, items with tests that had both a significant P-value after Bonferroni multiple testing correction[20 (at α=0.05) and a large effect size[25, 26] are highlighted with a grey background (details on CodeOcean[2], /code/Documentation/Statistical Reporting.xlsx). The statistical significance ensures a low probability of false positive results (aka, α), while the large effect size indicates practically noticeable effects even on a few samples.

Item	Figure n°	n	Statistical test used	n Bonferroni	P-value (after multiple correction)	Effect size classification
1	2b	382 total: 8-24 per strain	Simulated sweep (spherical): Descriptive only	N.A.	N.A.	N.A.
2	2c	296 total: 4-24 per strain	Simulated sweep (bulk): Descriptive only	N.A.	N.A.	N.A.
3	2d	342 total: 6-24 per strain	Simulated sweep (compact): Descriptive only	N.A.	N.A.	N.A.
4	2e	377 total: 8-24 per strain	Simulated sweep (porous): Descriptive only	N.A.	N.A.	N.A.
5	2g E1	34 total; 4-15 per E value	GLM: G'_0 vs. E	20	**1.31×10^{-27}**	**Large**
6	2g E2	1665; 5 bootstrap evaluations per E	LR + bootstrapping: softening strain vs. E	20	1	(Negligeable)
7	2g E3		GLM + bootstrapping: plateau G' vs. E	20	**2.02×10^{-22}**	**Large**
8	2g E4		GLM + bootstrapping: yield strain vs. E	20	1	(Negligeable)
9	2g Packing 1	23 total; 4-15 per ϕ	LR: G'_0 vs. ϕ	20	**2.91×10^{-10}**	**Large**
10	2g Packing 2	1016; 5 bootstrap evaluations per ϕ	LR + bootstrapping: softening strain vs. ϕ	20	**2.51×10^{-4}**	**Large**
11	2g Packing 3		LR + bootstrapping: plateau G' vs. ϕ	20	0.319	(Large)
12	2g Packing 4		LR + bootstrapping yield strain vs. ϕ	20	**0.0322**	**Large**
13	2g Friction1	22 total: 7-15 per μ	LR: G'_0 vs. μ	20	0.969	(Medium)
14	2g Friction2	743 ;	LR + bootstrapping: softening strain vs. μ	20	**3.46×10^{-4}**	**Large**
15	2g Friction3		LR + bootstrapping: plateau G' vs. μ	20	0.380	(Large)

16	2g Friction4	5 bootstrap evaluations per μ	LR + bootstrapping: yield strain vs. μ	20	1	(Negligeable)
17	2G double dagger (Fig. S3)	6342; Bootstr. 5 per geometry and μ	LR + bootstrapping: Within each particle geometry, yield strain vs. μ	3	**Spherical : 3.51x10^{-17}**<br>Porous : 0.67<br>Non porous :1.0	**Spherical: Large**<br>Porous irregular: (Small)<br>Non-porous irregular: (Negligeable)
18	2g Porosity 1	22 total: 7-15 per porosity (present/absent)	LR: G'_0 vs. porosity	20	1	(Negligeable)
19	2g Porosity 2	719; 5 bootstrap evaluations per porosity	LR + bootstrapping: softening strain vs porosity	20	0.56	(Large)
20	2g Porosity 3		LR + bootstrapping: plateau G' vs. porosity	20	**4.66x10^{-6}**	**Large**
21	2g Porosity 4		LR + bootstrapping: yield strain vs. porosity	20	1	(Medium)
22	2g shape1	23 total: 8-15 per shape (spherical /irregular)	LR: G'_0 vs. shape	20	1	(Negligeable)
23	2g shape2	759: 5 bootstrap evaluations per shape	LR + bootstrapping: softening strain vs shape	20	1	(Large)
24	2g shape3		LR + bootstrapping: plateau G' vs. shape	20	**1.07x10^{-3}**	**Large**
25	2g shape4		LR + bootstrapping: yield strain vs. shape	20	**6.91x10^{-5}**	**Large**
26	2h	65 total: 4-15 per condition	GLM, Low strain G' (i.e. G'_0) as a function of 3 explanatory variables	3 (posthoc tests)	**Overall: 2.68x10^{-88}**<br>**Young modulus: 8.04x10^{-88}**<br>Particle irregularity: 0.288<br>Porosity: 0.502	**Overall: Large**<br>**Young modulus: Large**<br>Particle irregularity: (Negligeable)<br>Particle porosity: (Negligeable)
27	S12	7 (EPI), 5 (S200), 3 (Juv)	t-test: yield strain in different materials	3	**Juv vs. S200: 0.011**<br>**EPI vs. S200: 1.4x10^{-5}**<br>EPI vs. Juv: 1	**Juv vs S200: Large**<br>**EPI vs S200: Large**<br>Juv vs EPI: (Large)
28	3f	5 individual curves shown	Illustrative only: rheology EPI	N.A.	N.A.	N.A.

29	3h	5 vs. 5 @Relative stress of $5x10^{-4}$, 6 vs. 5 @Relative stress of $1x10^{-3}$, 7 vs. 7 @Relative stress of $1x10^{-2}$ 3 vs.5 @Relative stress of $5x10^{-2}$	t-test: EPI vs. Sephacryl S200: relative modulus (G'/G_0') at given relative stress	4	rel. stress $5x10^{-4}$: 0.92, rel. stress $1x10^{-3}$: 0.78, **rel. stress $1x10^{-2}$: $1.7x10^{-8}$** rel. stress $5x10^{-2}$: 0.52	relative stress $5x10^{-4}$:(medium) relative stress $1x10^{-3}$: (medium) **relative stress $1x10^{-2}$: Large** relative stress $5x10^{-2}$: (large)
30	3i (Rheology)	18 rheology curves (9 per material)	(Least squares fitting for theory lines, see Figure S10)	N.A.	N.A.	N.A.
31	3i (Pore size)	S200: 4 samples (7 images), EPI: 5 samples (34 images)	(Least squares fitting for theory lines, see Figure S10)	N.A.	N.A.	N.A.
32	3g	3 (Juv), 1 (EPI)	Illustration only: comparative rheology	N.A.	N.A.	N.A.
33	S14, S15, Table S6	Regression on 12 averaged points (S15), obtained from a total of 116 measurements	LR: max force as a function of 2 explanatory variables + interaction	3 (posthoc tests)	**Overall: $3.6x10^{-7}$ injection rate: $9.79x10^{-6}$ EPI vs. DI: $1.89x10^{-6}$ Interaction: $8.26x10^{-5}$**	**Overall: Large Injection rate: Large EPI vs. DI: Large Interaction: Large**
35	3k	Total 15: 5 samples, each ejected 3x	LR: Young Modulus against injection number	1	0.96	(Negligeable)
37	3l	3 per curve shown (2 independent samples + repeatability)	Illustration only: self-healing	N.A.	N.A.	
38	3m	Same dataset as 3l	Illustration only: self-healing	N.A.	N.A.	
43	4d initial EPI vs Juv	5 per group	Wilcoxon comparing aspect ratio	4	1.0	(Negligeable)
44	4d (EPI shaping)	5 per goup	t-test, paired, comparing aspect ratio, after vs. before shaping	4	**0.014**	**Large**
45	4d (3 days)	5 per group	t-test, unpaired, comparing aspect ratio Juv vs. EPI	4	**0.029**	**Large**
46	4d (21 days)	5 per group	t-test, unpaired, comparing aspect ratio Juv. Vs. EPI	4	**0.011**	**Large**
47	4e EPI vs. Juv	20 vs. 16	t-test, unpaired on inflammation score	3	0.33	(Medium)

			(L+M+2*G+P): weights from [27]			
48	4e shaped vs. unshaped	16 vs. 20	t-test, unpaired on inflammation score (L+M+2*G+P) [27]	3	0.74	(Small)
49	4e, 3 vs. 12 weeks	18 vs. 18	t-test, unpaired on inflammation score (L+M+2*G+P) [27]	3	**3.0×10^{-8}**	**Large**
51	4f	Total 36 independent samples, 3-5 per experimental condition. 5 fields of view averaged for each sample	LR, capsule thickness as a function of 3 explanatory variables	1	**Overall: P=3.0×10^{-10}**<br>Time-point: 1.8×10^{-3}<br>Shaping: 0.11<br>**Material 9.6×10^{-10}**	**Overall: Large**<br>in individual, single regressions:<br>Time-point: (Medium)<br>Shaped: (Small)<br>**Material: Large**
52	4g	30 independent samples, 5 per experimental condition. 5 fields of view averaged for each sample	LR, Colonization % as a function of 2 explanatory variables	1	**Overall: 2.0×10^{-12}**<br>**Time-point: 4.4×10^{-9}**<br>**Material 6.5×10^{-5}**	**Overall: Large**<br>in individual, single regressions:<br>**Time-point: Large**<br>**Material: Large**
53	4h	20 independent samples, 5 per experimental condition. 5 fields of view averaged for each sample	Chi-squared: Presence/absence vascularization, depending on material (EPI/Juv) at 3 weeks and 3 months	2	**0.0033**	**Large**
54	4h	20 independent samples, 5 per experimental condition. 5 fields of view averaged for each sample	LR: vessel count vs. Time	2	**5.2×10^{-7}**	**Large**
59	5c	65 total; 3-20 per material	Hotelling test[28] or spatial rank sum[29] for pairwise distance	30	Pairwise test for difference in location in bivariate plot, using the Mahalanobis distance[30], details and test results on CodeOcean[2]	

60	5c	65, assigned to 4 clusters (unsupervised)	ANOVA (variance explained by Ward clustering[31] in the bivariate diagram)	1 (global test)	**8×10^{-26}**	**Large**
61	5d	34 total; 4-10 per neutralization level	Descriptive only: Change in rheology sweeps upon charge neutralization	N.A.	N.A.	N.A.
62	5e	Measurements: 29 total, 4-16 per coating, Simulations: 325 total, 4-13 per condition (strain and μ)	Descriptive only: Change in rheology sweeps upon collagen coating, compared with simulated change due to friction coefficient μ	N.A.	N.A.	N.A.
63	4m	13	LR: CMC vs. Time	N.A.	**3.5×10^{-4}**	**Large**
64	5f	23 total, 4-10 per coating condition	t-tests relative yield stress, different coatings vs. uncoated	3	1% collagen: 0.19<br>3% collagen:0.22<br>**10% collagen: 1.9×10^{-6}**	1% collagen: (medium)<br>3% collagen:(large)<br>**10% collagen: large**
65	5g		t-tests yield strain, different coatings vs. uncoated	3	1% collagen: 0.14<br>3% collagen: 0.76<br>10% collagen: 0.16	1% collagen: (large)<br>3% collagen:(medium)<br>10% collagen: (large)
66	5h	39 total, 9-10 per coating condition	t-test metabolic activity, different coatings vs. uncoated	3	1% collagen: 0.076<br>**3% collagen: 0.0058**<br>**10% collagen: 0.046**	1% collagen: (large)<br>**3% collagen: large**<br>**10% collagen: large**
73	S12b	7 (EPI), 5 (S200)	LR, yield strain as a function of 2 explanatory variables	2	Polymer concentration: 0.82<br>**Material identity: 2.1×10^{-5}**	**Large**
74	3j	11 (EPI), 4 (bulk)	t-test (maximum force, bulk vs. EPI)	3	**0.013**	**Large**
75	3j	11 (EPI), 4 (bulk)	Tolerance analysis[32] for max. force exceeding 20N[18] for bulk and EPI		**EPI: 3.7×10^{-4}**<br>Bulk: 1	**EPI: Large (by applying Cohen's criteria for d[25] to the K value)**<br>Bulk: (medium by applying Cohen's criteria to the K value)
77	S9a	70 particles from 3 independent experiments	Least squares fitting of core-shell model to intra-particle pore fraction, with profiling t-test[33]	3	**shell thickness: 2.2×10^{-26}**	**Large**

78	S9a		Model comparison for intra-particle pore fraction via ANOVA, AIC[34, 35]	3	**ANOVA: Model comparison:6.8×10^{-9}; AIC in favor of irregular core-shell over simple core-shell (ΔAIC=-35)**	**Large**
79	S9a		Profiling t-test[33] after non-linear regression in irregular domain core-shell model	N.A. (relative within model)	**shell thickness: best fit= 66μm, $P_{profiling}$=0.0045**<br><br>**domain limit: best fit=163μm, $P_{profiling}$=0.031**	**Overall: large**
80	S9a		Paired t-test against associated minority-minority contacts due to random interaction	3	**0.011**	**Large**
81	S9b		Wilcoxon test (Pore size, intra vs. 50 microns)	3	**4.80×10^{-10}**	**Large**
82	S9b		Wilcoxon test (inter vs. 50 microns)	3	**1.10×10^{-12}**	**Large**
83	S9b		Paired t-test (inter- vs. intra-particle pore size)	3	2.40×10^{-6}	Medium
84	S6a	97 in total, from 10 materials	t-test, unpaired or Wilcoxon. Comparison of roundness to EPI	9	**Alg. EPI: 0.012**<br>CMC Irr. NP: 1<br>Alg. Irr. NP: 0.29<br>Juv.: 1<br>**Cultisphere S: 3.7×10^{-5}**<br>**CMC Sph. NP: 3.3×10^{-4}**<br>**Alg. Sph. NP: 2.2×10^{-3}**<br>**S200: 6.6×10^{-6}**<br>**Cytodex: 0.016**	**Alg. EPI: Large**<br>CMC Irr. NP: Small<br>Alg. Irr. NP: Large<br>Juv.: Negligeable<br>**Cultisphere S: Large**<br>**CMC Sph. NP: Large**<br>**Alg. Sph. NP: Large**<br>**S200: Large**<br>**Cytodex: Large**
85	S6b	97 in total, from 10 materials	t-test, unpaired or Wilcoxon. Comparison of solidity to EPI	9	Alg. EPI: 0.48<br>**CMC Irr. NP: 0.003**<br>Alg. Irr. NP: 0.056<br>Juv.: 9.6×10^{-4}<br>Cultisphere S: 1<br>**CMC Sph. NP: 0.020**<br>**Alg. Sph. NP: 5.9×10^{-4}**<br>**S200: 0.023**<br>**Cytodex: 0.0016**	Alg. EPI: Large<br>**CMC Irr. NP: Large**<br>Alg. Irr. NP: Large<br>**Juv.: Large**<br>Cultisphere S: Small<br>**CMC Sph. NP: Large**<br>**Alg. Sph. NP: Large**<br>**S200: Large**<br>**Cytodex: Large**

87	S6c (Roundness)	97 from 10 materials	Threshold determination for roundness, Wilcoxon rank sum for classification.	2	**2.40x10^{-22}**	**Large**
88	S6c (solidity)	97 from 10 materials	Threshold determination for solidity, Wilcoxon rank sum for classification	2	**1.30x10^{-15}**	**Large**
89	S13a	4 vs. 31	Location difference of the yield region via t-test of log(G' at yield)-log(yield stress)	1	**2.8x10^{-28}**	**Large**
90	S13b	10 vs. 20	Location difference of the yield region via t-test of log(G' at yield)-log(yield stress)	1	**1.40x10^{-3}**	**Large**
91	S13c	65 in total, from 8 materials	T-test, unpaired or Wilcoxon rank sum<br>on yield strain, tests against regular EPI	7	HA. EPI: 1<br>Alg. EPI: 0.32<br>**CMC Irr. NP: 0.0036**<br>Juv.: 0.25<br>**CMC Sph. NP: 0.0013**<br>**S200: 0.00024**<br>**Cultisphere S: 0.0013**	HA EPI: medium<br>Alg. EPI: medium<br>**CMC Irr. NP: large**<br>Juv.: (large)<br>**CMC Sph. NP: large**<br>**S200: large**<br>**Cultisphere S: large**
92	S13d		T-test, unpaired or Wilcoxon rank sum on relative yield stress, tests against regular EPI	7	HA. EPI: 1<br>3 Alg. EPI: 0.092<br>**CMC Irr. NP: 1.7x10^{-5}**<br>**Juv.: 0.037**<br>**CMC Sph. NP: 1.2x10^{-4}**<br>S200: 1.0<br>**Cultisphere S: 2.2x10^{-7}**	HA. EPI: medium<br>**Alg. EPI: large**<br>**CMC Irr. NP: large**<br>**Juv.: large**<br>**CMC Sph. NP: large**<br>S200: medium<br>**Cultisphere S: large**
93	3d	19 samples from 3 materials (5-7 per material). This represents analysis of 484 individual particles	Unpaired t-tests roundness, testing vs. EPI	2	Juv. vs. EPI: 1;<br>**S200 vs EPI: 1.48x10^{-6}**	Juv. vs EPI: negligible;<br>**S200 vs EPI: large**
94	3e		Unpaired t-test or Wilcoxon rank sum, on solidity against EPI	2	**Juv. vs EPI: 0.00021;**<br>**S200 vs EPI: 0.005**	**Juvederm vs EPI: large**<br>**S200 vs EPI: large**

Table S7. Overview statistical evaluation. The data in Table S7 summarizes the statistical evaluation for the test and descriptive statistics used. The tests are organized per figure, details for both main and supporting figures being reported. Each test or group of associated tests is assigned a unique Item id, permitting referral from main text, this supporting information and also CodeOcean scripts[2]. Table S7 reports sample size,

test method, Bonferroni multiple correction factor, P-value after Bonferroni multiple testing correction[20], and effect size[25, 26]. Additional information on the items contained in Table S7 as well as CodeOcean only figures can be found on CodeOcean[2] (at /code/Documentation/Statistical Reporting.xlsx). The statistical reporting document on CodeOcean[2] " Statistical Reporting.xlsx" referes to the same Item id nomenclature, but provides further details (methodological notes, confidence intervals, normality testing and mitigation strategies, degrees of freedom where applicable, notes on hierarchical testing, and, for full reproducibility, the path to the scripts used to perform figure plotting and statistical evaluation). The following abbreviations apply in Table S7: N.A.: Not applicable; LR: Linear regression; GLM: General linear model; Irr.: Irregular; NP: Non-porous; Sph.: Spherical; S200: Sephacryl S200; Juv.: Juvéderm Voluma (HA. Control), CMC: Carboxymethylcellulose, Alg.: Alginate, ANOVA: Analysis of Variance, AIC: Akaike Information Criterion[35]

P-value < 0.05 (after Bonferroni correction[20]) and large effect size[25, 26] (for at least one of the tests if there are several).

13. Methods

A methods summary is provided in the main text of the manuscript. Here, further experimental and methodological details required for detailed reproduction are given.

13.1. Simulation

The simulation was implemented as a custom Python library "particleShear"[36], installable by standard Python installation from the Python package distribution server PyPI. Runnable examples are configured in the CodeOcean capsule "An injectable meta-biomaterial" accompanying this manuscript[2], including extensive documentation (the capsule is also archived with the main raw data set associated with this publication[37]). The simulation implements the physical interaction framework defined by Otsuki at al.,[1] corresponding to central viscoelastic interaction and tangential friction between spherical particles.

A main difficulty with discrete particle simulations is the numerical evaluation of stress tensors[38, 39]. For both mathematical (eigenvalues) and fundamental reasons, stress tensor evaluation needs to be symmetric[38-40], ideally to within numerical precision[40]. Inappropriate approximations, particularly regarding conservation of angular momentum[40], but also implementation errors can cause asymmetry. Here, we used the stress tensor evaluation framework provided by Nicot et al,[38] along with unit testing (Part 4 of the manual at /code/Documentation/Simulation particleShear/Manual particleShear.pdf on CodeOcean[2]) to rigorously ensure symmetric stress tensor evaluation. Indeed, after correction of a series of errors detected by this method (minor integration mistake in eq. 29 in ref. ,[38], corrected as eq. S1-24 in the CodeOcean[2] manual; angular momentum imbalance due to known boundary effects[40], but also due to radius change in dynamic elastic compression and removal of the asymmetric inertia correction term by Otsuki et al, [1]), we obtained symmetric stress tensors including at very high deformation amplitudes, as desired, to within numerical precision. Further, to avoid complete interpenetration of neighboring particles at large shear, we added a non-linear term to the repulsive elastic interaction. We ensured that in the small compression limit, we recover the linear repulsion law used by Otsuki et al.[1] (eq. S1-5b of the manual on CodeOcean[2]).

Most importantly for the meta-material design, we also implement the possibility of permanent crosslinks between neighboring spheres. The permanent crosslinks remain intact regardless of the geometrical separation of the spheres, generating attractive forces if the spheres are separated beyond touching distance. They also do not allow for frictional slippage. Example simulation videos are provided as Supplemental videos S1-S4, which aside from random initialization can be reproduced on CodeOcean[2].

To quantitatively match simulations and rheological stress sweeps (Figure 5e), we further introduced a plateau contact law to better match the plateau response in uniaxial testing of bulk precursor. This approach described in further detailed in Figure S18, and the CodeOceanOnly results section (CodeOcean capsule[2], folder /results/Figures_Codeocean_only, figures C12 and C13, description in CodeOceanOnlyResults.pdf in the same folder) .
The simulations were run on the Baobab cluster of the University of Geneva (total ca. 30'000 CPU hours). The raw output data, an overview database, and data analysis routines are installed in replicable form on CodeOcean[2].

13.2. Statistics

Statistical evaluation and graphing were done using the R-Cran free software, version 3.2.3 to 4.0.2.[41] All statistical analysis is further available in reproducibly runnable format on CodeOcean[2]. This includes automated install of a series of custom R libraries written for the purpose of compression[42], rheology[43] and Python simulation data analysis[44], as well as plotting[45] and validation of reproducible computing[46]. Central tendency was reported by arithmetic mean values, and variability by indication of single standard deviations. For comparisons with 5 or less measurements per group, or where graphically feasible, individual values were overlayed onto barplots (main Figures 3d, 3e, 4e-4h, 5f-5h, and supporting Figures S6a, S6b, S9a, S13c and S13d). Linear regression was used to assess continuous effects, and paired or unpaired, Student (t) tests were used to compare individual conditions, with P-values reported after Bonferroni multiple testing correction[20] for all tests performed per subfigure. For all parametric tests, normality of the residuals was assessed with the Shapiro-Wilks test[21] (and Royston test[22] for multivariate data, see below). If significant deviation was found, an alternative test was evaluated: generalized linear models with identity link but expectation-value dependent variance (quasi-likelihood estimators[47]) to accommodate heteroscedasticity in linear regression, and Wilcoxon signed rank (paired) or Mann-Whitney U (unpaired) for binary comparison, or multivariate equivalents (see below). The tests performed are reported in Table S7, including sample sizes and effect sizes[25, 26]; additional details (among others, confidence intervals, degrees of freedom, details of normality testing, and associated CodeOcean scripts and output files) are available on CodeOcean[2] (path: /code/Documentation/Statistical Reporting.xlsx).

For the statistical evaluation of the characteristic strains and stresses of the simulated elastic storage modulus and viscous loss modulus curves (G' and G'') as a function of the model parameters (Figure 2g) a bootstrapping approach was taken.[48] For a given set of model parameters, 5 subsets among the simulations carried out for the various strain amplitudes are drawn randomly with a 20% probability for a given simulation to be included. For each subset, slightly different estimations for characteristic stresses and strains as defined in Figure 2f are thus obtained. By linear regression of these values against the physical parameter being varied, the P-values depicted in Figure 2g are obtained after Bonferroni correction.[20] To reduce dependence of the P-value on the random draw, we averaged the underlying F-statistics over 500 bootstrapping runs.[48]

13.2.1. Statistical analysis of inflammatory cell counts for Fig. 4e

In Fig. 4e in the main text, the number of lymphocytes L, macrophages M, giant foreign body cells G and polymorphonuclear cells P counted per field of view (0.36mm^2) are reported for Juvéderm Voluma (HA control) and EPI biomaterial implants at 3 and 12 weeks.

For Fig. 4e, we combined the L (lymphocytes), M (Macrophages), G (Giant foreign body cells) and P (Polymorphonuclear cells) counts into a single inflammation score. For this, we make use of a scoring scheme proposed in ISO-10993-6[27]. In Table E.1 of the norm[27], we notice that the boundaries for increasing severity scores are identical for the L, M and P counts, but are about twice as low for the giant foreign body cells G. Based on this, we define a weighted score as $S=L+M+P+2\cdot G$. Statistical testing for Fig. 4e is therefore done based on the global S score.

13.2.2. Bivariate clustering

For the analysis of the bivariate yield point characteristics (strain and relative yield stresss) of various microhydrogel suspensions (Figure 5c), we used appropriate multivariate analogs to the Student t, Shapiro-Wilks and Wilicoxon tests. Parametric testing was done using the Hotelling test.[28] To assess the underlying multivariate normality assumption, we used the Royston test.[22] Finally, if non-normality was detected, we used non-parametric spatial rank testing[29] instead of the Hotelling test. For clustering analysis of this data (Figure 5c), we estimated Mahalanobis distances between materials; this is an effect size measure as the Mahalanobis distance indicates distance relative to variability.[30] While using a common intra-material variance-covariance matrix for all points to maximize precision, we compensated for the larger absolute spread in materials with higher yield strain or relative yield stress values by using the log of the Mahalanobis distance[30] for the construction of the dendrogram by the Ward[31] algorithm. Distinct groups in the clustering dendrogram were defined by an effect size equivalent to at least 4.4 standard deviations. This guarantees less than 5% overlap between individual measures with Bonferroni correction.

13.3. Reagents

1,4-Piperazinediethanesulfonic acid (PIPES, ref P6757-500G), adipic acid dihydrazide (ADH, ref A0638-100G), 1-ethyl-3-(-(3-dimethylaminopropyl)carbodiimide (EDC, ref E1769-25G), rhodamine 6G hydrochloride, 4′,6-diamidino-2-phenylindole (DAPI), 6-aminofluorescein, 37 % HCl solution, collagen I from bovine skin (C4243-20ML), and Sephacryl S200 HR were all purchased from Sigma-Aldrich. NaOH pellets were from Huberlab (Applichem Panreac, ref A3910, 1000). Carboxymethyl cellulose (AQUALON CMC 7LF PH, 90.5 KDa, DS: 0.84, ref 891158) was purchased from Ashland. EDTA Solution (0.5 M, pH 8.0) was obtained from ThermoFisher Scientific. Saline solution (0.9% NaCl) was purchased from Bichsel. Disposable sterile vacuum filtration systems (pore size 0.2 um, filter capacity 1000 mL) were purchased from Sigma-Aldrich, whereas cell strainers (40 µm) were obtained from VWR and cell strainers (100 µm) were obtained from Greiner Bio-one (ref 342000). Juvéderm Voluma® (HA control) was purchased from Direct Derma supplies.

13.4. Biomaterial synthesis

Cryogel scaffolds were synthesized as reported,[4] with minor modifications. Briefly, carboxymethyl cellulose (13.56 g), PIPES Buffer (6.30 g), adipic acid dihydrazide (486 mg) and NaOH pellets (1.20 g) were dissolved to a final volume of 1000 mL in deionized water and the solution filtered at 0.2 µm. This solution could be stored in a 4°C for up to a month prior to use in a fridge. Then EDC (2.70 g) was added. After mixing, the solution was filled into 35 mL plastic syringes. The syringes were closed with a syringe cap and then placed into a freezer set to -20°C.

After 2 days the syringes were removed from the freezer and allowed to reach room temperature. The EPI biomaterial was obtained by forceful extrusion through a 22G catheter. On a filter system, the biomaterial was washed with 10mM EDTA, followed by incubation with 2 M NaOH solution (2h), washing with physiological saline (3x). For *in-vivo* experiments the biomaterial was sterilized for 20 min at 121°C.

13.5. Control biomaterial synthesis

Alginate (Sigma, W201502-1KG) and hyaluronic acid (HTL, 0.83m^3/kg intrinsic viscosity) variants of the EPI biomaterial were synthesized using the cryogelation and extrusion-

fragmentation route as for the carboxymethylcellulose EPI material, with an increase of all the reactant concentrations by one third to counter-balance the softer nature of these materials found in preliminary trials.
Preliminary attempts at synthesis compact (non-porous) carboxymethylcellulose particles failed, since activation of the premix as described above at room temperature without freezing did not yield a firm gel. To mitigate this, we changed for carboxymethylcellulose of higher molecular weight (Haenseler Carmellosum sodicum 500) and increased the concentration to 50mg/mL. The reactant concentrations were finally: 50g/L CMC, 50g/L PIPES, 2.3g/L adipic dihydrazide, 10g/L NaOH, and 10g/L of EDC for activation. This reaction mix forms firm gels at room temperature.

To synthesize irregular compact particles, the reaction mix was let to polymerize in a flask overnight, collected in a syringe and then extruded using a 22G catheter as for the EPI biomaterial.
Spherical CMC particles were produced by emulsion polymerization in sunflower seed oil (Coop Switzerland). For this, 50mL of activated reaction mix (50mg/mL mix as described above, including EDC) was added to 200mL of oil. Polymerization was let to occur under constant, strong agitation (magnetic stir bar) in a 250mL Erlenmeyer recipient overnight. The resulting beads were retrieved with a tea sieve (0.7mm opening), rinsed with ethanol 70%, followed by abundant washing in deionized water. This was followed by size selection by sieving (retention of beads with diameters larger than 0.7mm, removal of beads with diameters larger than 2mm). The resulting beads had a mean diameter of 1.47+/-0.35mm (quantification from fluorescence images after Rhodamine 6G hydrochloride staining, 5 µg/mL in deionized water).

13.6. Microscopic imaging

Confocal images were captured on a confocal Zeiss LSM 700 driven by Zen 2010b version service pack 1 (Zeiss), using a 10x Plan Neofluar lens with a numerical aperture (NA) of 0.3 or a 20x PlanApochromat objective with NA=0.8. Alternatively, we also used a Zeiss LSM 800 driven by ZEN 2.3 with ZEN module Tiles (Zeiss), also using either 10x PlanApochromat lens with NA=0.45 or a 20x PlanApochromat with NA=0.8. Images of histological slides were acquired with a Leica DM750, with achromat lenses of 10x (HI PLAN, NA=0.25), 20x (HI PLAN, NA=0.4) or an Axio Scan.Z1 with a 20x PlanApochromat lens (NA=0.8) driven by Zen 2.0 (Blue edition) software suite. Also for slide scanning, we alternatively used an Olympus VS120-L100 slide scanner, with a 40x UPLSAPO lens (NA=0.9) driven by Olympus VS-ASW 2.9 software. Table S8 below details the microscope setup used for each experiment or figure.

Figure	Microscope / Objective	Adjustments
1f	Zeiss LSM 700, 10x	No adjustment
3a	Zeiss LSM 700, 10x	No adjustment
3b	Zeiss LSM 700, 20x	Contrast enhanced (weak contrast between crosslinked and liquid hyaluronic acid)
3c	Zeiss LSM 800, 20x	No adjustment
4i, 4j, 4k	Olympus Slide scanner VS120-L100, 40x	No adjustment<br>Rotation: 4i, 4j by 180°
4l	Axio Scan.Z1, 20x	No adjustment
5i	Zeiss LSM 700, 10x	No adjustment
5j	Axio Scan.Z1, 20x	Contrast enhanced

5k	Axio Scan.Z1, 20x	Contrast enhanced, 90° rotation

Table S8. *Microscopic imaging. Summary list of the microscopes and objectives used for the main figures. Further details on the microscopes and objectives in the text.*

For visualization and morphological quantification, the EPI biomaterial was stained with 5microgram/mL Rhodamine 6G hydrochloride (Figure 1 and 3a) or 4′,6-diamidino-2-phenylindole (DAPI, by affinity to the final material) or 6-aminofluorescein (by inclusion of 10 micromolar aminofluorescein into the synthesis mixture[4]), or DAPI and aminofluorescein (Figure 1). For the reference material, Sephacryl S200, auto-fluorescence images (excitation 353nm, emission 405nm and above) was used for visualization and quantification. Where possible, quantitative image evaluation was performed in an automated fashion using preconfigured macros tabulated in Excel files with the aid of a custom ImageJ plugin dedicated to this purpose[49].

For confocal imaging of suspensions, observation chambers avoiding both evaporation and mechanical compression were used. These chambers consisted of a 1.5mm high Perspex sheet (Evonik) cut to the shape of a microscope slide (75mm x 25mm) using a laser cutter (HobbyLaser, FullSpectrum Engineering, commandeered by FullSpectrum Laser RetinaEngrave3D, Version 4.423). A rectangular sample reservoir (30mm x 12.5mm) was further cut into these Perspex microscope slides. A microscope coverslide was glued permanently to the lower side of the Perspex microscope slide using bathroom silicone glue (Coop BricoLoisir, Switzerland). After filling with the sample to be observed by confocal microscopy, the observation chamber was closed by capillarity using a second microscope coverslide.

Scanning electron microscope SEM images were finally acquired with a Zeiss Merlin SEM, equipped with a Gemini II column and the ZeissSmartSEM acquisition software.

13.7. Pore size and pore fraction

After staining (for the EPI biomaterial) and confocal image acquisition, Fiji software[6] (ImageJ version 2.0.0-rc-44/1.50g) was used to visualize z-stack images, and to quantify pore size and fraction as well as particle size. For pore fraction quantification, images were acquired at a resolution so that several pores fit across the image, implying generally the use of a 10x objective for the EPI biomaterial vs. a 20x objective for the Sephacryl S200 control material. The EPI biomaterial was stained with rhodamine 6G (excited at 550 nm) whereas autofluorescence images (excited at 355nm,) were acquired for the Sephacryl S200 reference. The images were thresholded automatically using the Li algorithm,[50] built-in in Fiji. In case of evident gross misinterpretation of the images by the algorithm, thresholding was carried out manually instead. The wall and pore fraction could then be estimated from the fraction of white and black pixels; for pore size estimation, the maximal sphere fitting algorithm by Beat Münch et al.[11] was used (detailed method in the CodeOceanOnly results section, folder /results/Figures_Codeocean_only on CodeOcean[2], figure C3; ImageJ plugin used for automation available for download at [49]).

For assignment and analysis of intra-particle and inter-particle pore space, permanent green and red fluorescent labelling of particles were used. Confocal images of about 1:10 mixtures were analysed using a pore-space assignment algorithm developed as an ImageJ plugin for this purpose[14]. See Figure S8 for a detailed description of the method, and Figure S9 for the results.

13.8. Particle size, irregularity and porosity

Particle size distribution (Figure S5) was evaluated from large tile-stitched images acquired on dilute particle suspensions using a Zeiss LSM 800 with a 5x PlanApochromat objective, NA=0.16, after staining with rhodamine 6G as for pore size. Standard Fiji[6] routines were then used on the resulting large images: binarization by manual thresholding, digital filling of holes, and particle size analysis (minimal size 10 micrometers^2, circularity at least 0.1).

Irregularity and porosity were measured using confocal images (acquired on LSM 700 and 800) of EPI and HA control (both labelled with Rhodamine 6G as described above) as well as Sephadex 200 (autofluorescence). Using FIJI software, images were automatically thresholded with Li algorithm and particles were analyzed with the "Shape descriptor" ImageJ plugin[8]. Roundness and solidity were used to describe respectively the particle irregularity and porosity (Figure 3d and 3e). An extended set of materials was analyzed similarly, the analysis allowing to define thresholds for the roundness and solidity parameters to quantitatively distinguish spherical from irregular and porous from non-porous particles (Figure S6).

13.9. *In-vivo* biodegradability

Biodegradation of EPI was evaluated from the decrease in local carboxymethylcellulose concentration in visually identifiable EPI fragments on histological cuts. For this, histological sections where deparaffinized and rehydrated, prior to immersion of the cuts in a solution of Rhodamine 6G at 5 µg/ml into PBS for 5 min. Then, cuts were mounted using 5 µl of Rhodamine 6G solution in PBS and coverslips were sealed using nail polish. Cuts were then imaged in fluorescence mode, acquiring confocal images on a Zeiss LSM 700 in such a way that the background intensity (free Rhodamine 6G solution) ranged between about 10% and 20% of the full intensity range, while avoiding saturation on visually identified biomaterial fragments. From the confocal images, the fluorescence intensity in identified EPI regions was measured and compared to the one of the background (neither tissue material nor gel). This yielded an estimation of the partition coefficient K of rhodamine 6G between free PBS solution and the EPI biomaterial. Under the assumption of a Donnan equilibrium for a monovalent cation,[51] the fixed negative charge concentration can be from the partition coefficient K calculated as:

$$c_{COO^-}=(K-1/K)*s_0 \qquad \text{eq. S15}$$

where s_0=0.15 mol/L is used for the ionic concentration of PBS. With a degree of substation of 0.84 for the sodium carboxymethylcellulose according to the supplier's certificate of analysis, one calculates a mean molecular weight per COO^- group of 273 g/mol. This allows estimation of the mass concentration (in g/L) from the molar concentration c_{COO^-}.

13.10. *In vitro* culture and collagen type I modifications

To ensure proper cell adhesion, EPI biomaterial was covalently modified using collagen type I from bovine skin (Sigma C4243) under sterile conditions, as previously described with minor modifications[52]. Briefly, EPI particles were dehydrated and washed several times using PBS 1X, followed by a rinse in Na_2HPO_4, 100mM, ca. pH 4. Then, EPI particles were incubated 5 min in a solution of collagen type I diluted in acetic acid buffer (pH 4, 100mM, diluted such as to obtain dry weight ratios of 0%, 1%, 3% and 10% collagen I relative to

crosslinked CMC). After dehydration, particles were immerged in a solution of EDC (1 mg/ml) in MES (0.5M, pH 5.5) water for 10 min. After sequential washing with DI water and PBS, EPI particles were then either stored in PBS 1X at 4°C for rheology or seeded with cells for particle confluence measurement and imaging.

For cell seeding, biomaterials were first dehydrated and washed with PBS 1X and transferred into cell culture dishes. Mesenchymal cells (OP9[53]) were seeded at approximately 600.000 cells/mg dry mass respectively onto unmodified or collagen type I (1%, 3%, 10%) modified EPI biomaterials. Long-term adhesion (5 days) was evaluated by confocal microscopy after fixation and staining for actin.

Short-term adhesion was assessed Alamar Blue quantifiaction after separation of adherent and non-adherent cells after 1h of incubation at 37°C. For this, the cell seeding was carried out on a cell strainer (100 µm) suspended above a cell culture well. After the incubation period, the adherent cells were transferred with the biomaterial to a new well, while recovering the non-adherent fraction by gentle washing. The number of viable adherent and non-adherent cells was then quantified using the Alamar Blue assay (1962643, Invitrogen, USA) according to the manufacturer's instructions in kinetic mode. For this, fluorescence was monitored at 550 nm excitation and 590 nm emission on a Spectramax Paradigm plate reader (Molecular Devices, Firmware V1.2 b103 04.03.2015; driven by Softmax Pro 7.1 build 246936), the final volume for each assay was 1.2mL in a 24-well plate. A standard with known numbers of OP-9 cells was run for each plate, allowing to relate the rate of fluorescence increase to the cell number. The fraction of adherent cells was evaluated relative to total of viable cells detected in each assay.

13.11. Rheology

Rheological measurements were carried out on a Haake Rheostress RS100 5Ncm apparatus, using the RheoWin software (RheoWin Job Manager: version 3.61.0005) to control the apparatus and acquire data. To avoid wall slipping, roughened surfaces were obtained by gluing a rough cleaning cloth (Miobrill, Migros Switzerland, ref. 7065.206 / 15.02.2330) with hot glue (UHU, LT110, local hardware store) to the surfaces in contact with the material. Experiments were generally conducted in a custom cup geometry. A plate-plate geometry (Haake PP20, ref. 222-0586, also with roughened surfaces) was used where the sample volume was too low (HA control sample for Figure 3g ; parameter studies requiring many samples for Figure 5b-5g). Further details and comparison of the different geometries are provided on CodeOcean[2], CodeOceanOnly results section (folder /results/Figures_Codeocean_only), figures C4-C6, and table C1 in CodeOceanOnlyResults.pdf. A solvent trap (Haake ref. 222-0607) was used whenever possible to limit sample evaporation.

With the exception of the self-healing experiments, we generally preconditioned the material with an oscillatory stress sweep from 1-10 Pa and back at 0.2Hz before applying the desired shear protocols to minimize the impact of shear history. In rare cases of very dilute material, we found this to induce significant shear softening or even yielding, in which case we changed to a preconditioning sweep from 0.1 Pa to 1Pa and back, still at 0.2Hz. After data acquisition, we exported the data to text files (RheoWin Data Manger: version 3.61.0005) and completed data treatment and graphing in R-Cran, based in part on custom plugins available for download[43, 45].

Rheological master curves represent the elastic modulus G' normalized to the plateau value at low stress.[54] They are obtained by normalizing G' curves with respect to the low-strain G_0' plateau value.[54] We evaluate the low-strain limit G_0' as the average of the G' values for the measurement points with low applied shear stress (τ<2Pa). We further take care to exclude points showing already an onset of softening or liquefaction by also imposing $G''(\tau)<0.1*G'(\tau)$. We also normalize the applied stress τ to G_0'. After visual verification that indeed all the different G' curves obtained at different polymer concentrations collapse onto a single master curve after normalization of both the G' and τ, we obtain the main master curve by averaging of the normalized curves, along with evaluation of the standard deviation. For the more horizontal part of the master curve ($G'/G_0'>0.5$ for the Sephacryl S200 master curve, $G'/G_0'>0.1$ for the EPI biomaterial) we perform vertical averaging of the G'/G_0' associated with a given interval of applied stress τ, whereas for the steepest part of the master curves, we rather average the τ associated with a given interval of G'/G_0' values.

13.12. Uniaxial compression and injectability testing

We performed uniaxial compression testing on a TextureAnalyzer TA.XT plus machine by Stable Microsystems, using the TestMaker program (Version 4.0.6.0) supplied by the manufacturer to program the tests, and the TextureExponent 32 program (Version 4.0.13.0), also supplied by the manufacturer, to run them. After acquisition, the data was exported as text and analyzed using R-Cran, in part based on custom libraries also available for download[42, 45].

For compression analysis of the elastic porous injectable (EPI) biomaterial, a disk of 20mm diameter and approximately 6mm height was shaped under a 20mm diameter chuck. The chuck was then moved by feedback to the zero force condition. This defined the original sample height, usually in the range between 5 and 6mm depending on the actual amount of sample. From this position, compression by 50% of the height at a speed of 0.01mm/s was then carried out, acquiring the force and position data. The force was low-pass filtered to reduce noise, and was then converted to stress by dividing through the contact area (circle of 10mm radius), whereas deformation was expressed as deformation strain relative to original sample height.

For injectability analysis, a 1mL syringe (BD, ref 303172) was loaded with test material (EPI biomaterial, deionized water, or air) or synthesized directly in the syringe for testing bulk material. The syringes were equipped with a 20-Gauge needle (Terumo, ref NN2070S, outer diameter 0.9mm, length 70mm, Figure 3j) with a blunt delivery cannula (Thiebaud Biomedical devices, ref. F9020100, outer diameter 2mm, length 10cm, Figure 3k and supporting Figures S14 and S15). The syringe was then placed on custom holder. The piston was then moved by using the TextureAnalyzer XT Plus machine at the desired rate, while recording force and distance. For bulk ejection, higher forces were required and we used a Mecmesin MultiTest 2.5 dV testbench with a maximum force of 2.5kN, equipped with a Mecmesin AFG 100 force gauge (100N), driven by Vector Pro Lite v6.5.0.0.

13.13. Animal experiments

All *in-vivo* experiments were approved by the Animal Care and Use Committee of the Canton of Vaud, Switzerland (Authorization VD 3063 and VD 3629). Female, adult CD1 mice between 12 and 20 weeks of age were obtained from Charles River (Bar Harbor, Maine, USA) and allowed to acclimatize in the animal facility for at least 1 week prior to

implantation. Room temperature was kept at 22±2°C with 12 hours light/dark cycle and normal diet *ad libitum*.

Prior to injection, animals were anesthetized with 4% (2% for maintenance) isoflurane (Animalcare Ltd), using an ophthalmic gel (Viscotears, Alcon) for eye protection. The area for injection was shaved and disinfected with betadine (Mundipharma Medical Company). For injection, a small access was created in the skin with a 18G needle, followed by injection of biomaterial samples (2 injection sites per animal, or max. 400 µL into single site) through a 20G catheter (BD Biosciences). No sutures were required. Animals were monitored weekly throughout the study.
When shaping was first attempted with the HA control, the material was found to temporarily slide to the side, unless excessive force was applied, which would however unduly enlarge the injection pouch. As we also observed some swelling, we limited the maximum injection volume for HA control to 200 µL and refrained from shaping in subsequent studies. Indeed, forced shaping as attempted here is not the intended use of HA fillers.
At pre-defined time points the macroscopic dimensions of the implants were assessed with a calliper (day 0, after 3 days, after 3 weeks). For histological evaluation (at 3 weeks for shaped samples, at 6 months and 1 year for unshaped 200 µL samples), mice were sacrificed by intraperitoneal injection of sodium pentobarbital (150mg/kg). Harvested samples were further immersed for 24 hours in 4% paraformaldehyde at 4°C, washed 3x with PBS and embedded in paraffin following routine procedures. 4 µm slices were stained with hematoxylin/eosin in an automated slide processor (Prisma special stainer, Tissue-Tek; Glas G2 coverslipper from Sakura). For cell transplantation, GFP^{+}-OP9[53] were seeded onto collagen-modified EPI (3% collagen per weight), cultured for 24h, followed by partial dehydration of the scaffold material and subcutaneous injection into adult NSG mice (Jackson laboratories, 5557).[53] GFP-positive cells were subsequently labelled by immunohistochemistry with an anti-GFP antibody (Abcam, ab6673, diluted 1:400, overnight, 4°C, 3,3'-Diaminobenzidine DAB revelation).

13.14. Magnetic resonance imaging

Magnetic resonance imaging (MRI) images were acquired at 3 days post-implantation on a Varian INOVA console with 14.1 T magnet of 26cm horizontal bore (Magnex Scientific, Abingdon, UK) and a gradient coil (maximum 400 mT/m) with a fix rise time of 120 ms. A home-built single loop surface coil of 24 mm of diameter was used as a transceiver positioned on top of the tissue graft.
During measurements, each adult mouse was anesthetized with 1.5–2% isoflurane in mixture of air and O_2 (50/50%) and subsequently placed supine within an adapted holder. Body temperature was maintained at 37°C using a thermoregulated water circuit. Monitoring respiration with a respiration cushion was used for triggering during all acquisitions.
Spin echo intensity images were acquired with a repetition time of 1.5s and echo times at 14ms and again at 25ms (the overall intensity of this second echo is used for the images shown). The acquisition matrix was 96×192 pixels for a field of view of 15×25 mm, with 46 slices spaced by 0.05 mm. The acquisition time was on average 12min, with minor differences due to respiratory gating.

13.15. Replication

Unless explicitly stated otherwise, replicated experiments were performed on distinctly prepared biomaterial samples. We further used 3 separate 1L synthesis batches and various smaller batches. Thus the samples contain variability associated with sample preparation and

chemical synthesis. However, we used the same chemical suppliers, so while some commercial lot-to-lot variation is reflected in the data, supplier change would not be. We also measured rheological properties in both plate-plate and cylinder geometries to ensure a certain independence on the measurement geometry.

Regarding numerical reproducibility, we systematically evaluated software updates by comparing novel evaluation runs of the raw data back to the initial reference evaluation on CodeOcean[2, 46]. Between different evaluations (local computers vs. CodeOcean, and also successive versions of R, Python and our own libraries in CodeOcean), the numerical changes were generally less than 10^{-14}, although particularly updates in least squares fitting routines in some instances lead to changes up to 10^{-6}. Regardless, these numerical errors remain small compared to empirical errors, more typically situated in the 10^{-2} to 10^{-1} range.

14. Bibliography

[1] M. Otsuki, H. Hayakawa, Phys Rev E 2017, 95, 062902.
[2] A. Beduer, F. Bonini, C. A. Verheyen, M. Genta, M. Martins, J. Brefie-Guth, A. Filippova, P. Burch, T. Braschler, CodeOcean Capsule 2021, https://doi.org/10.24433/CO.6934377.v1.
[3] L. Yoo, V. Gupta, C. Lee, P. Kavehpore, J. L. Demer, Biomech Model Mechanobiol 2011, 10, 901.
[4] A. Beduer, T. Braschler, O. Peric, G. E. Fantner, S. Mosser, P. C. Fraering, S. Bencherif, D. J. Mooney, P. Renaud, Adv Healthc Mater 2015, 4, 301.
[5] G. Schramm, *A Practical Approach to Rheology and Rheometry*, Gebrueder HAAKE GmbH, Karlsruhe, Germany 1994.
[6] J. Schindelin, I. Arganda-Carreras, E. Frise, V. Kaynig, M. Longair, T. Pietzsch, S. Preibisch, C. Rueden, S. Saalfeld, B. Schmid, J. Y. Tinevez, D. J. White, V. Hartenstein, K. Eliceiri, P. Tomancak, A. Cardona, Nat Methods 2012, 9, 676.
[7] S. de Bournonville, L. Geris, G. Kerckhofs, Sci Rep 2021, 11, 2819; R. Alfred, J. T. Taiani, R. J. Krawetz, A. Yamashita, D. E. Rancourt, M. S. Kallos, Biomaterials 2011, 32, 6006; C. E. Nweke, J. P. Stegemann, J Mater Chem B 2020, 8, 3972; A. A. Akasha, "Attachment of Embryonic Stem Cells-derived Cardiomyocytes in Cultispher-S Microcarriers by using Spinner flask", 2012.
[8] G. Chinga, https://imagej.nih.gov/ij/plugins/descriptors.html retrieved on 2020-06-24.
[9] M. Kadic, G. W. Milton, M. van Hecke, M. Wegener, Nature Reviews Physics 2019, 1, 198; A. H. D. Cheng, *Poroelasticity: Theory and Applications of Transport in Porous Media*, Vol. 27, Springer, ebook 2016.
[10] C. Pellet, M. Cloitre, Soft Matter 2016, 12, 3710.
[11] B. Münch, ftp://ftp.empa.ch/pub/empa/outgoing/BeatsRamsch/lib/, 2015; B. Münch, L. Holzer, Journal of the American Ceramic Society 2008, 91, 4059.
[12] A. Beduer, N. Piacentini, L. Aeberli, A. Da Silva, C. A. Verheyen, F. Bonini, A. Rochat, A. Filippova, L. Serex, P. Renaud, T. Braschler, Acta Biomater 2018, 76, 71; Y. C. Chiu, M. H. Cheng, H. Engel, S. W. Kao, J. C. Larson, S. Gupta, E. M. Brey, Biomaterials 2011, 32, 6045.
[13] A. Artel, H. Mehdizadeh, Y. C. Chiu, E. M. Brey, A. Cinar, Tissue Eng Part A 2011, 17, 2133.

[14] J. Brefie-Guth, T. Braschler, feretPore: Pore size and attribution analysis in multi-particle systems with different colors, Zenodo, https://doi.org/10.5281/zenodo.4945115, 2021.
[15] P. N. Patel, C. K. Smith, C. W. Patrick, J Biomed Mater Res A 2005, 73a, 313.
[16] I. D. Evans, A. Lips, J Chem Soc Faraday T 1990, 86, 3413.
[17] T. Bhattacharjee, C. P. Kabb, C. S. O'Bryan, J. M. Uruena, B. S. Sumerlin, W. G. Sawyer, T. E. Angelini, Soft Matter 2018, 14, 1559.
[18] M. H. Chen, L. L. Wang, J. J. Chung, Y. H. Kim, P. Atluri, J. A. Burdick, Acs Biomater Sci Eng 2017, 3, 3146.
[19] L. J. Gibson, M. F. Ashby, *Cellular Solids: Structure and properties*, Cambridge University Press, Cambridge, UK 1997.
[20] J. M. Bland, D. G. Altman, BMJ 1995, 310, 170.
[21] S. S. Shapiro, M. B. Wilk, Biometrika 1965, 52, 591.
[22] P. Royston, Statistics and Computing 1992, 2, 117.
[23] J. A. Nelder, R. W. M. Wedderburn, Journal of the Royal Statistical Society. Series A (General) 1972, 135, 370.
[24] A. F. Siegel, Biometrika 1982, 69, 242.
[25] J. Cohen, Psychological Bulletin 1992, 112, 155.
[26] J. Cohen, *Statistical power analysis for the behavioral sciences*, L. Erlbaum Associates, Hillsdale, N.J. 1988.
[27] ISO technical committee: ISO/TC 194: ISO 10993-6:2016 Biological evaluation of medical devices — Part 6: Tests for local effects after implantation
[28] H. Hotelling, Ann. Math. Statist. 1931, 2, 360.
[29] H. Oja, R. H. Randles, Statist. Sci. 2004, 19, 598.
[30] P. C. Mahalanobis, Proceedings of National Institute of Sciences (India) 1936, 2, 49.
[31] J. H. Ward, J Am Stat Assoc 1963, 58, 236; F. Murtagh, Journal of Classification 2014, 31, 274.
[32] K. Krishnamoorthy, T. Mathew, *Statistical Tolerance Regions: Theory, Applications, and Computation*, Wiley, Hoboken, New Jersey, USA 2009; D. S. Young, The R Journal 2016, 8, 200; W. G. Howe, J Am Stat Assoc 1969, 64, 610.
[33] D. M. Bates, D. G. Watts, *Nonlinear Regression Analysis and Its Applications (Chapter 6)*, John Wiley & Sons, United States 1988.
[34] J. N. Rouder, C. R. Engelhardt, S. McCabe, R. D. Morey, Psychonomic Bulletin & Review 2016, 23, 1779.
[35] Y. Sakamoto, M. Ishiguro, G. Kitagawa, *Akaike Information Criterion Statistics*, D. Reidel Publishing Company, KTK Scientific Publ., Dordrecht, Tokyo 1986.
[36] T. Braschler, particleShear: Discrete Python particle simulation with digital rheology and stress tensor evaluation (Version v1.0.2), Zenodo, https://doi.org/10.5281/zenodo.4589212, 2021.
[37] A. e. a. Beduer, Data: An injectable meta-biomaterial. Raw data set at Zenodo, Beduer et al., DOI 10.5281/zenodo.4600443, 2021.
[38] F. Nicot, N. Hadda, M. Guessasma, J. Fortin, O. Millet, Int J Solids Struct 2013, 50, 2508.
[39] J. Fortin, O. Millet, G. de Saxce, Eur J Mech a-Solid 2003, 22, 567.
[40] B. Yan, R. A. Regueiro, Int J Solids Struct 2019, 161, 243.
[41] R. C. D. Team, R Foundation for Statistical Computing, Vienna, Austria 2015.
[42] P. Burch, M. Braschler, T. Braschler, textureAnalyzerGels: R package for importing and analyzing hydrogel compression data (Version v1.0), Zenodo, https://doi.org/10.5281/zenodo.4589276, 2001.

[43] T. Braschler, rheologyEvaluation: R package to read and analyze rheowin text export files v1.1 (Version v1.1), Zenodo, https://doi.org/10.5281/zenodo.4594353, 2021.
[44] T. Braschler, particleShearEvaluation: Import and analysis of output files generated by the discrete particle simulation Python module particleShear (Version v1.0), Zenodo, https://doi.org/10.5281/zenodo.4594649, 2021.
[45] T. Braschler, plot.counts: R package with convenience functions for plotting count data (v1.0), Zenodo, https://doi.org/10.5281/zenodo.4589498
, 2021.
[46] T. Braschler, reproducibleCalculationTools: R package for comparing numeric output from successive evaluations, Zenodo, https://doi.org/10.5281/zenodo.4594515
, 2021.
[47] S. L. Zeger, K. Liang, in *Encyclopedia of Biostatistics*, (Eds: P. Armitage, T. Colton), John Wiley & Sons, 2005.
[48] R. Nisbet, G. Miner, K. Yale, in *Handbook of Statistical Analysis and Data Mining Applications (Second Edition)*, (Eds: R. Nisbet, G. Miner, K. Yale), Academic Press, Boston 2018, 215.
[49] T. Braschler, PoreSizeExcel: ImageJ plugin for automated pore size evaluation, Zenodo, https://doi.org/10.5281/zenodo.4589546, 2021.
[50] C. H. Li, P. K. S. Tam, Pattern Recognition Letters 1998, 19, 771.
[51] F. G. Donnan, Chemical Reviews 1924, 1, 73.
[52] L. Serex, T. Braschler, A. Filippova, A. Rochat, A. Beduer, A. Bertsch, P. Renaud, Adv Mater Technol-Us 2018, 3.
[53] D. N. Tavakol, J. Tratwal, F. Bonini, M. Genta, V. Campos, P. Burch, S. Hoehnel, A. Beduer, M. Alessandrini, O. Naveiras, T. Braschler, Biomaterials 2020, 232, 119665.
[54] P. Menut, S. Seiffert, J. Sprakel, D. A. Weitz, Soft Matter 2012, 8, 156.
